# Chromosome-level genome assembly of *Plazaster borealis* sheds light on the morphogenesis of multiarmed starfish and its regenerative capacity

**DOI:** 10.1093/gigascience/giac063

**Published:** 2022-07-09

**Authors:** Yujung Lee, Bongsang Kim, Jaehoon Jung, Bomin Koh, So Yun Jhang, Chaeyoung Ban, Won-Jae Chi, Soonok Kim, Jaewoong Yu

**Affiliations:** Department of Research, eGnome, Inc., 26 Beobwon-ro 9-gil, Sonpa-gu, Seoul 05836, Republic of Korea; Department of Research, eGnome, Inc., 26 Beobwon-ro 9-gil, Sonpa-gu, Seoul 05836, Republic of Korea; Department of Agricultural and Life Sciences and Research Institute of Population Genomics, Seoul National University, Seoul 08826, Republic of Korea; Department of Research, eGnome, Inc., 26 Beobwon-ro 9-gil, Sonpa-gu, Seoul 05836, Republic of Korea; Department of Agricultural and Life Sciences and Research Institute of Population Genomics, Seoul National University, Seoul 08826, Republic of Korea; Department of Research, eGnome, Inc., 26 Beobwon-ro 9-gil, Sonpa-gu, Seoul 05836, Republic of Korea; Department of Research, eGnome, Inc., 26 Beobwon-ro 9-gil, Sonpa-gu, Seoul 05836, Republic of Korea; Interdisciplinary Program in Bioinformatics, Seoul National University, Seoul 08826, Republic of Korea; Department of Research, eGnome, Inc., 26 Beobwon-ro 9-gil, Sonpa-gu, Seoul 05836, Republic of Korea; Department of Microorganism Resources Division, National Institute of Biological Resources, Incheon 22689, Republic of Korea; Department of Microorganism Resources Division, National Institute of Biological Resources, Incheon 22689, Republic of Korea; Department of Research, eGnome, Inc., 26 Beobwon-ro 9-gil, Sonpa-gu, Seoul 05836, Republic of Korea

**Keywords:** Plazaster borealis, genome assembly, Hi-C, Nanopore

## Abstract

**Background:**

*Plazaster borealis* has a unique morphology, displaying multiple arms with a clear distinction between disk and arms, rather than displaying pentaradial symmetry, a remarkable characteristic of echinoderms. Herein we report the first chromosome-level reference genome of *P. borealis* and an essential tool to further investigate the basis of the divergent morphology.

**Findings:**

In total, 57.76 Gb of a long read and 70.83 Gb of short-read data were generated to assemble a *de novo* 561-Mb reference genome of *P. borealis*, and Hi-C sequencing data (57.47 Gb) were used for scaffolding into 22 chromosomal scaffolds comprising 92.38% of the genome. The genome completeness estimated by BUSCO was 98.0% using the metazoan set, indicating a high-quality assembly. Through the comparative genome analysis, we identified evolutionary accelerated genes known to be involved in morphogenesis and regeneration, suggesting their potential role in shaping body pattern and capacity of regeneration.

**Conclusion:**

This first chromosome-level genome assembly of *P. borealis* provides fundamental insights into echinoderm biology, as well as the genomic mechanism underlying its unique morphology and regeneration.

## Data Description

### Context

Echinoderms are marine animals characterized by the following 3 remarkable characteristics: (i) extensive regenerative abilities in both adult and larval forms [1, 2]; (ii) the water vascular system used for gas, nutrient, and waste exchange [3]; and (iii) extraordinary morphological characteristics, including pentaradial symmetry [4, 5].

Pentaradial symmetry has been observed in all extant classes of echinoderms. Echinoids (sea urchin) and holothurians (sea cucumber) always have 5 ambulacral grooves, and crinoids have many arms in multiples of 5 that branch out from the 5 primary brachia [4, 5]. Most species of asteroids and ophiuroids are 5-armed, but many exceptions are scattered across the tree of Echinodermata. Extant asteroids are distinguished by 34 families, including 20 families of only 5-armed species, 9 families of both 5-armed and multiarmed species, and 5 families with exclusively multiarmed species [6]. However, most multiarmed forms have arm numbers that cannot be divided into 5, raising questions about the arm development mechanisms that do not follow the pentaradial symmetry.

**Figure 1:**
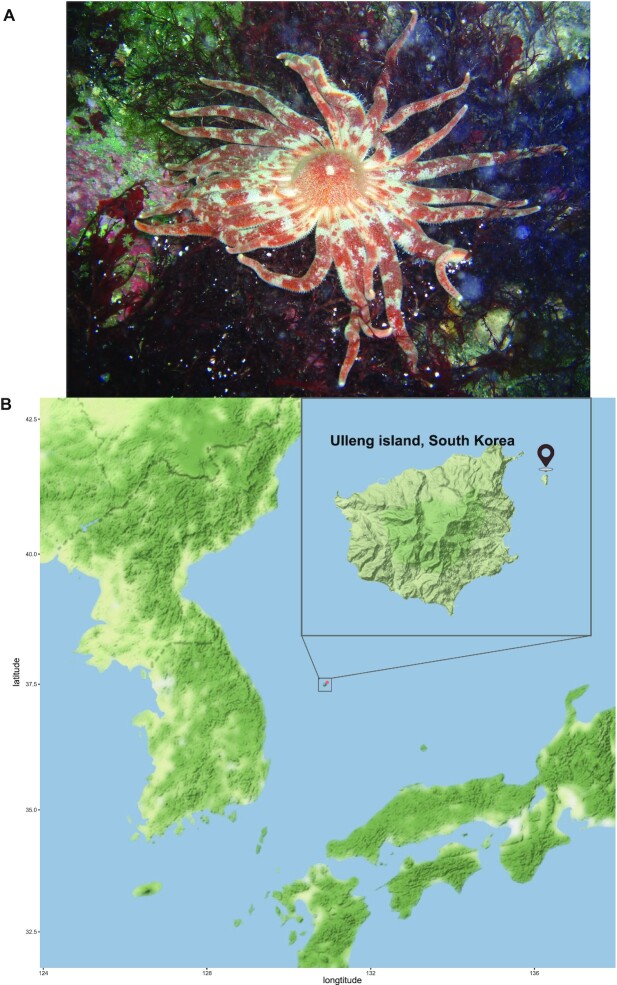
(A) Adult *Plazaster borealis*. Photograph by National Institute of Biological Resources [77]. (B) Sampling spot of *P. borealis* studied in this research.

The octopus starfish, *Plazaster borealis* (NCBI:txid466999; marinespecies.org:taxname:254846), is a starfish that inhabits the water that surrounds Korea and Japan [7, 8]. It belongs to the family Labidiasteridae, one of 5 exclusively multiarmed families [6]. Fig. 1A illustrates a unique morphology of *P. borealis* that the number of arms is around 31 to 40, which is a large number among multiarmed starfishes, and it shows a clear differentiation between arms and central disks [9].

In a previous study of *P. borealis*, Matsuoka et al. [10] investigated the molecular phylogenetic relationship of 5 species from the order Forcipulatida: *Asterias amurensis, Aphelasterias japonica, Distolasterias nipon, Coscinasterias acutispina*, and *P. borealis. P. borealis* was the most closely related to 5-armed *A. amurensis* and distantly related to multiarmed *C. acutispina*. The result suggested that the unique morphology of *P. borealis* might have descended from a 5-armed starfish, which possibly resulted from accelerated sequence evolution. However, the absence of a reference genome has limited in-depth research. To understand the genetic basis of the specialized morphology of the starfish, we sequenced the genome of *P. borealis* and performed comparative genomic analyses with the high quality of well-annotated genome sequences of 6 other echinoderms (*Asterias rubens, Acanthaster planci, Patiria miniata, Lytechinus variegatus, Parastichopus parvimensis*, and *Strongylocentrotus purpuratus*).

### Chromosome-level genome assembly of the octopus starfish

We estimated the genome size of *P. borealis* with GenomeScope [40] to be ∼497 Mb (Supplementary Fig. S1). A comprehensive sequencing data set was generated for the *P. borealis* genome assembly based on this estimation. From the Nanopore sequencing platform, a total of 57.76 Gb long read was yielded with 116× coverage. Using the Illumina sequencing platform, 142× coverage of Illumina short paired-end read sequencing data and 115× coverage of Hi-C paired-end reads were generated (Supplementary Table S1). Moreover, we sequenced 25.63 Gb of RNA Illumina short paired-end reads and 7.28 Gb of RNA Nanopore long reads to construct transcriptome assembly utilized for annotation.

A draft genome assembly was generated, consisting of 179 contigs totaling 561 Mb with an N50 of 11 Mb (Supplementary Table S2). We then scaffolded the contigs using Hi-C data with 3D-DNA to obtain chromosomal information [11]. The total size of the final assembly was 561 Mb, comprising 22 chromosome-level scaffolds with a contig N50 of 24 Mb. These 22 chromosome-level scaffolds comprised 92.48% of the assembly, although the remaining 42 Mb were unanchored and required further investigation (Table 1, Supplementary Fig. S2). This number is consistent with chromosome results of other species of the order Forcipulatida, supporting the accurate chromosome number acquired in the current study.

**Table 1:** *Plazaster borealis* assembly statistics

Assembly statistics	Value
Genome size (bp)	561,050,340
Number of scaffolds	801
Number of chromosome-scale scaffolds	22
N50 of scaffolds (bp)	24,975,817
L50 of scaffolds	10
Chromosome-scale scaffolds (bp)	518,884,334
GC content of the genome (%)	38.89
QV score	36.3457
Error rate	0.00023
BUSCO analysis	
Library	Metazoan_odb10
Complete	935 (98.0%)
Complete and single copy	925 (97.0%)
Complete and duplicated	10 (1.0%)
Fragmented	11 (1.2%)
Missing	8 (0.8%)

### Completeness of the assembled genome

The genome completeness was evaluated using BUSCO [12] with the metazoan data set called “metazoan_odb10.” As a result, a total of 935 (98.0%) core metazoan genes were successfully detected in the genome, consisting of 97.0% single-copy, 1.0% duplicated, 1.2% fragmental, and 0.8% missing genes from the metazoan data set. We also estimated the overall assembly quality by comparing the *k*-mer distribution of the assemblies and the Illumina short-read sets using Merqury [13]. The genome assembly of *P. borealis* showed high-quality values (QV > 36) with an error rate of 0.00023 (Table 1). Additionally, the GC content of *P. borealis* was 38.89%, which was very similar to that of *A. rubens* (38.76%) and *P. ochraceus* (39.01%), the species of the order Forcipulatida. The assessment results validated the high quality of our final genome assembly. To our knowledge, this is the first high-quality chromosome-level genome assembly for *P. borealis* and the first reference genome of the family Labidiasteridae.

### Annotation of repeats and genes

Repetitive elements accounted for 51.05% of the whole genome assembly, and detailed percentages of the predominant repetitive element families are summarized in Table 2. We annotated a total of 26,836 genes onto the assembled regions. Compared with other starfish, *P. borealis* has a similar average exon length (213 bp) and exon number per gene (7.19), but it has a shorter intron length (1,261 bp) than *A. rubens* (eAstRub1.3). BUSCO benchmarking value of this gene set was summarized as 92.6% of complete genes, including 90% single-copy, 2.6% duplicated, 4.6% fragmental, and 2.8% missing genes from the metazoan data set. Following a standard functional annotation, we observed that 24,248 (96.13%) genes were successfully annotated with at least one related functional assignment (Table 3).

**Table 2:** *Plazaster borealis* repetitive DNA elements

Type	Number of elements	Length occupied (bp)	Percentage of sequence (%)
DNA	10,734	3,597,965	0.64
LINE	42,851	3,472,043	0.62
SINE	60,394	13,931,402	2.48
LTR	8,277	5,145,127	0.92
Satellite	9	2,752	0
Small RNA	20,889	1,464,546	0.26
Simple repeat	162,149	8,016,020	1.43
Unclassified	1,294,477	249,314,223	44.44
Low complexity	25,170	1,365,485	0.24
Total			51.05

**Table 3:** *Plazaster borealis* genome annotation statistics

Statistic	Value
Number of predicted genes	26,836
Number of predicted protein-coding genes	25,224
Average gene length	8,948.89
Number of transcripts	26,737
Average transcript length (bp)	1,502.90
Number of exons	192,343
Average exon length (bp)	213.57
Average exon per transcript	7.19
Number of introns	165,606
Average intron length (bp)	1,261.88
Number of genes annotated to Swiss-Prot	18,451
Number of genes annotated to PFAM	18,541
Number of genes annotated to NR	24,229
BUSCO analysis	
Complete (%)	884 (92.6%)
Complete and single copy (%)	859 (90.0%)
Complete and duplicated (%)	25 (2.6%)
Fragmented (%)	44 (4.6%)
Missing (%)	26 (2.8%)

### Phylogenetic and syntenic relationship

**Figure 2:**
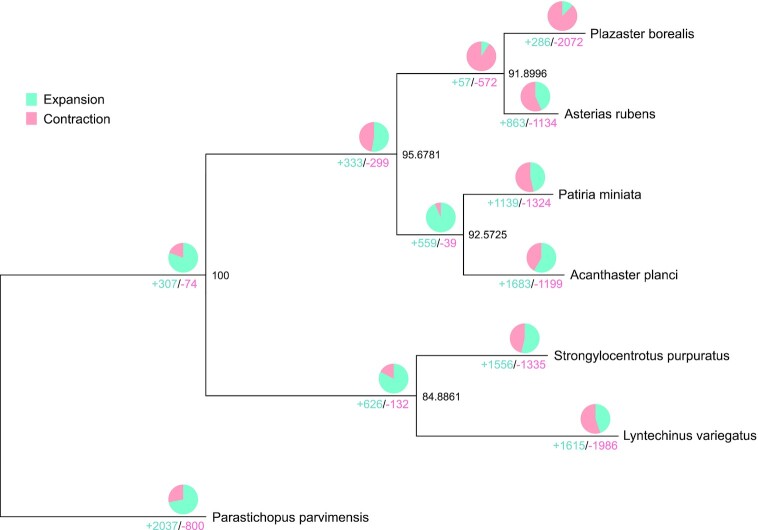
A phylogenetic tree of *P. borealis* and 6 other species. This tree was constructed using protein sequences of 7 species, showing gene family expansion and contraction. The number below the branches represents the number of gene families with either expansion (blue) or contraction (red). The ratio of expanded and contracted gene families is expressed in the pie chart above the branches. The numbers at the node indicate the bootstrap value. The species used in the tree are *P. borealis, A. rubens, A. planci, P. miniata, L. variegatus, P. parvimensis*, and *S. purpuratus*.

To understand the phylogenetic placement of *P. borealis*, a species tree was inferred from sets of multicopy gene trees with the STAG algorithm [75] based on protein sequences from 7 echinoderm genomes: *P. borealis, A. rubens, A. planci, P. miniata, L. variegatus, P. parvimensis*, and *S. purpuratus. P. borealis* was the most closely related to *A. rubens* (Fig. 2), consistent with both previous results [10].

**Figure 3:**
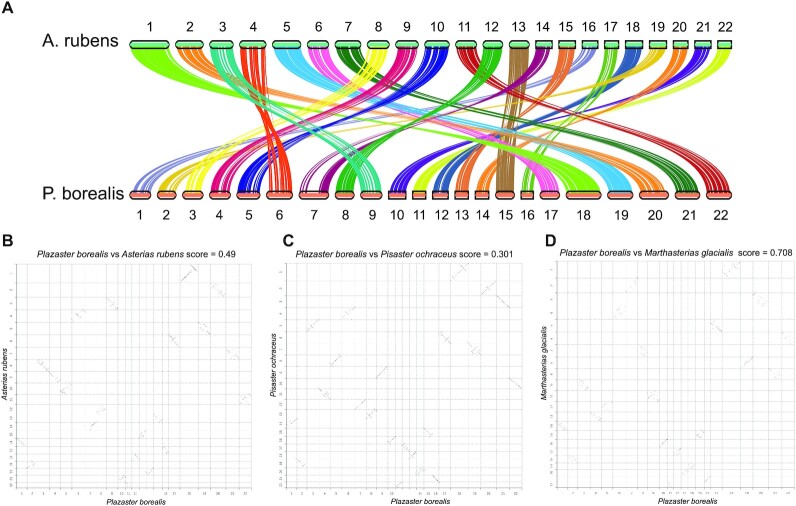
Syntenic relationship of *P. borealis* and species of the order Forcipulatida. (A) Synteny between *A. rubens* and *P. borealis*. The syntenic blocks were calculated with MCscan. (B–D) Syntenic relationship of *P. borealis* between *A. rubens* (B), *Pisaster ochraceus* (C), and *Marthasterias glacialis* (D). Genomic sequences were compared with Chromeister based on inexact *k*-mer matching.

Syntenic relationships as inferred by MCscan [14] results were congruent with the phylogenetic results from the STAG analyses. In the genome of *P. borealis* and *A. rubens*, every chromosome matched each other well enough to suggest that the entire chromosomes seem to be highly conserved, except an additional genomic region detected in chromosome 7 of *P. borealis* (Fig. 3A, B). A similar tendency, using Chromeister [15], was observed with other species of the order Forcipulatida, *P. ochraceus* and *M. glacialis. P. borealis* exhibited more conservation of synteny with *P. ochraceus* than *A. rubens*, which seems to be influenced by the observed genomic region. We also analyzed synteny of *P. borealis* with *A. planci*, the starfish of a different order; however, chromosomes were not matched. These results suggest that genomes within the Forcipulatida order are remarkably conserved in terms of synteny, allowing us to confirm the high quality of our genome assembly.

### Gene family evolution in *P. borealis*

**Figure 4:**
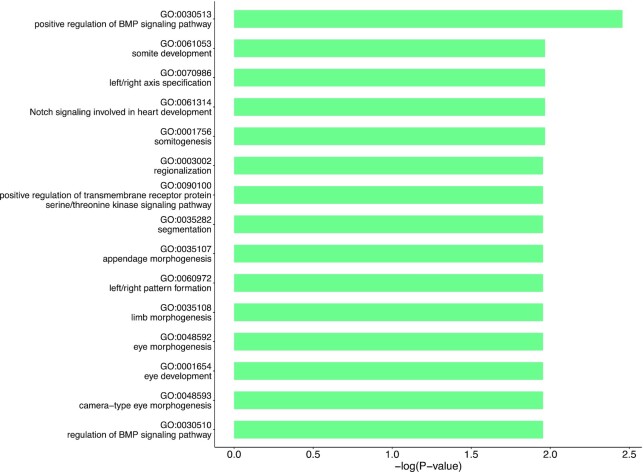
GO enrichment analysis of expanded gene families of *P. borealis*.

Based on the assumption that the unique morphology of *P. borealis* is explained by accelerated evolutionary rate [10], we performed comparative genomic analyses among 7 echinoderm species. Although the genetic mechanism underlying the development of supernumerary arms of starfish is elusive, we hypothesized that genes associated with tissue morphogenesis are increased to produce excessive arms. We tested this hypothesis by performing expansion and contraction analyses of gene families using CAFE5 [16]. Compared with 6 echinoderm species, 286 gene families were expanded, whereas 2,072 gene families were contracted in *P. borealis* (Fig. 2). The significantly expanded genes in the genome of *P. borealis* were significantly enriched in categories of Notch and BMP signaling pathways, body pattern specification, morphogenesis, and eye development (*P*< 0.02) (Fig. 4). Collectively, these expanded gene families are likely to play an enhanced role in forming supernumerary arms of *P. borealis*. Notch and BMP signaling are evolutionally conserved and play multiple roles during animal development, especially in regulating body patterns. The Notch signaling pathway is essential for cell proliferation, cell fate decisions, and induction of differentiation during embryonic and postnatal development [17–19]. Besides regulating cell-fate decisions at an individual cell level, a cell-to-cell signaling mechanism of Notch coordinates the spatiotemporal patterning in a tissue [20]. In *Drosophila melanogaster*, Notch functions as it is required to specify the fate of the cells that will eventually segment the leg and develop the leg joint [21, 22]. The mechanisms of BMP gradient formation have been studied in various animals. BMP2/4 signaling study of sea urchin showed that interaction between BMP2/4 and chordin formed the dorsal-ventral gradient and resulted in dorsal-ventral axis patterning [23]. Furthermore, as the physical characteristic of starfish, their eyes exist at the end of each arm, denoting that arm development is accompanied with eye development. However, contracted gene families of *P. borealis* had no significantly enriched functions, except GTPase regulator activity (GO:0030695, *P* = 0.005647). Gene repertories of *P. borealis* showed differences in the contents of other species’ expanded and contracted genes mainly enriched in terms related to the nerve development (Supplementary Table S3).

**Figure 5:**
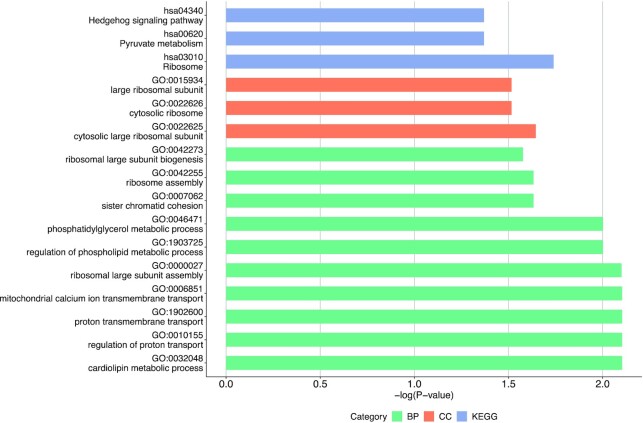
Results of GO enrichment analysis of positively selected genes. BP, GO Term Biological Process (green); CC, GO Term Cellular Component (red); KEGG (blue).

In addition, we identified 607 gene families unique in *P. borealis* consisting of 2,631 genes and 111 one-to-one orthologous genes between *P. borealis* and 6 other species. The gene families unique in *P. borealis* are enriched for the following Gene Ontology (GO) terms: apoptotic cell clearance, positive regulation of epithelial cell proliferation, vascular transport, and activation of JNKK activity (Supplementary Table S4). The enriched term, activation of JNKK activity, is involved in the JNK pathway, which promotes apoptosis by upregulating proapoptotic gene expression [24]. Typically, cell proliferation and death are important to achieve tissue formation, involving changes in cell number, size, shape, and position [25]. Based on these findings, the presence of additional genes of the Notch pathway, BMP pathway, and JNK pathway involved in body pattern specification, cell proliferation, and apoptosis could indicate enhanced tissue shaping to form many arms.

The signaling pathways that underwent gene family expansion in the *P. borealis* lineage, especially the Notch and BMP pathways, also play several key conserved roles in the regeneration of many species. For example, in the study of brittle stars, the inhibition of Notch signaling hindered arm regeneration and downregulated genes related to extracellular matrix (ECM) component, cell proliferation, apoptosis, and innate immunity, which are biological processes associated with regeneration [26]. In addition, previous studies of echinoderm gene expression and other animals showed that Notch and BMP signaling are the principal pathways for tissue regeneration [27, 28].

The studies of the metamorphosis of multiarmed starfishes led to the proposal of the “Five-Plus” hypothesis [6, 29]. It states that 5 primary arms generated concurrently develop in a controlled unit and supernumerary arms are produced in separate and independent pathways. Although these pathways are still uncertain, Hotchkiss [6] suggested 2 possibilities: postgeneration of arms in the incompletely developed starfish or intercalated regeneration of arms in adults. The capacity of regeneration is a remarkable feature of all extant classes of echinoderms [2]. Thus, it is possible that multiarmed starfishes could transform from 5-rayed forms to multirayed forms by growing new arms through regeneration-related mechanisms, thus suggesting that genes in these families may play critical roles in the biosynthesis and metabolism processes of its unique body plan as well as in regeneration processes.

Using *P. borealis* as the foreground branch and 6 other echinoderm species as the background branches, we incorporated the branch-site model in the PAML package to detect positively selected genes. A total of 14 genes were positively selected in *P. borealis* (*P* < 0.05, BEB > 0.95) and significantly enriched in GO terms related to “lipid metabolism,” “transport of proton,” “pyruvate metabolism,” and “Hedgehog signaling pathway” (Fig. 5, Supplementary Table S5). It is worth noting that these positively selected genes also included BMP4, which regulates regeneration and tissue specification (Table 4).

**Table 4:** Genes with accelerated evolution in the *P. borealis*

Gene	H0_lnl	H1_lnl	Likelihood ratio	FDR	No. of positively selected sites*
GPR161	−8,827.28	−8,798.95	56.66761	2.06E-13	5
RPL5	−3,991.54	−3,968.12	46.84587	2.3E-11	1
RSL24D1	−2,215.1	−2,192.93	44.35075	6.59E-11	14
PHB2	−4,815.8	−4,805.98	19.631658	1.61E-05	4
NAA10	−4,703.42	−4,694.3	18.237898	2.92E-05	4
IQCA1	−9,112.13	−9,103.79	16.684644	5.88E-05	2
SLC30A5	−10,574.5	−10,566.6	15.766218	8.6E-05	3
BMP10	−8,017.18	−8,010.17	14.034764	0.000196	4
STOML2	−5,414.16	−5,408.06	12.206464	0.000476	1
ACYP1	−1,855.62	−1,849.54	12.153438	0.000452	3
NIPSNAP3A	−4,951.12	−4,946.47	9.296206	0.001968	1

H0_lnl: log likelihood given H0 (ω does not vary across the branches), H1_lnl: log likelihood given H1, *Number of positively selected sites with a BEB (Bayes Empirical Bayes) of > 0.95.

Regeneration is a high-energy-required process in which starfishes in the regeneration state increase the amount of lipid and energy in the pyloric caeca to use [30]. GPR161 and BMP4, well-known genes to be critical in regeneration, were also detected as positively selected genes. The G-protein coupled receptor Gpr161 negatively regulates the Hedgehog pathway via cAMP signaling, known to participate in the process of tissue regeneration [31, 32]. Additionally, previous studies of planarian regeneration indicate that BMP4 is a key for tissue specification, especially dorsal-ventral polarity, which may explain the distinctive disk of *P. borealis* [33]. Together with those of previous studies, our results further suggest that related genes may have contributed to the regeneration and development of the unique body plan of *P. borealis*, with multiple arms. Therefore, *P. borealis* can be potentially regarded as a valuable model to investigate the mechanisms underlying supernumerary arm development and regeneration. This high-quality genome is a useful and valuable genetic resource for future research, especially in a unique body plan and regeneration biology.

## Conclusion

The first chromosome-level *P. borealis* genome was assembled and annotated. Twenty-two chromosomal scaffolds are constructed with N50 of 24.97 Mb, which showed high conservation with genomes of 3 starfish species of the order Forcipulatida. Furthermore, we identified the accelerated evolution of *P. borealis* in the context of genomics, which may explain its multiarmed morphology and regenerative capacity. The availability of the high-quality genome sequence of *P. borealis* is expected to provide many insights into the unique morphology of multiarmed starfish and their regeneration. Regarding the scientific value of *P. borealis*, the genome and gene inventory resulting from this study will be helpful in future research on these critical topics.

## Methods

### Sampling and genomic DNA extraction

Adult specimens of *P. borealis* were sampled at a depth of 31 m near Ulleung island, Korea (latitude: 37.53390, longitude: 130.93920) (Fig. 1A). *P. borealis* was dissected with scissors to obtain gonad, pyloric caecae, stomach, and epidermis of an arm. Isolated tissues were frozen on dry ice immediately and kept at −80°C until further processing. Then, the frozen tissues were ground into a fine powder with liquid nitrogen using a pestle and mortar for nucleic acid extraction.

High molecular weight (HMW) DNA was obtained from the gonad following a nuclei isolation method [34]. Genomic DNA was obtained from the gonad following a modified cetyltrimethylammonium bromide (CTAB) protocol [35] in the presence of 2% polyvinylpyrrolidone (1% of molecular weight [MW] 10,000 and 1% of MW 40,000) (Sigma-Aldrich, Burlington, MA, USA). DNA concentration was determined using the Quant-iT PicoGreen® assay (Invitrogen, Waltham, MA, USA), and the absorbance at 260 nm and 230nm (A260/A230) was measured in the Synergy HTX Multi-Mode microplate reader (Biotek, Rochester, VT, USA). Their quality was verified by gel electrophoresis.

### High-throughput sequencing of genomic DNA

For Nanopore sequencing, short genomic fragments (<10 kb) were removed using a Short Read Eliminator Kit (Circulomics, Baltimore, MD, USA). The library was prepared using the ONT 1D ligation Sequencing kit (SQK-LSK109, Oxford Nanopore Technologies, Oxford, UK) with the native barcoding expansion kit (EXP-NBD104) in accordance with the manufacturer's protocol. In brief, genomic DNA was repaired using the NEBNext FFPE DNA Repair Mix (New England BioLabs, Ipswich, MA, USA) and NEBNext Ultra II End Repair/dA-Tailing Module. The end-prepped DNA was individually barcoded with ONT native barcode by NEB Blunt/TA Ligase Master Mix (New England BioLabs). Barcoded DNA samples were pooled in equal molar amounts. The DNA was ligated with adapter using the NEBNext Quick Ligation Module (New England BioLabs). After every enzyme reaction, the DNA samples were purified using AMPure XP beads (Beckman Coulter, Brea, CA, USA). The final library was loaded onto a MinION flow cell (FLO-MIN106 and FLO-MIN111, R9.4 and R10.3) (Oxford Nanopore Technologies) and PromethION flowcell (FLO-PRO002) (Oxford Nanopore Technologies). Sequencing was performed on a MinION MK1b and PromethION sequencer (PromethION, RRID:SCR_017987) with MinKNOW software (19.10.1).

We also used an Illumina platform to generate short high-quality sequencing reads. DNA library was prepared using TruSeq DNA PCR-Free (Illumina, San Diego, CA, USA), and we evaluated the distribution of fragment sizes with TapeStation D1000 (Agilent Technologies, Santa Clara, CA, USA). Finally, the DNA library was sequenced in the Illumina NovaSeq 6000 (Illumina NovaSeq 6000 Sequencing System, RRID:SCR_016387) with the length of 150-bp paired-end reads.

Hi-C technology was also employed for chromosome-level genome assembly. Hi-C library construction protocol is as follows. Ground gonad tissue was mixed with 1% formaldehyde for fixing chromatin, and then the nuclei were isolated following a nuclei isolation method [1]. Fixed chromatin was digested with HindII-HF (New England BioLabs), and we filled the 5′ overhangs with nucleotides and biotin-14-dCTP (Invitrogen) and ligated free blunt ends. After ligation, we purified DNA and removed biotin from unligated DNA ends. Fragmentation and size selection were performed to shear the Hi-C DNA. Hi-C library preparation was performed using the ThruPLEX® DNA-seq Kit (Takara Bio USA, Inc, Mountain View, CA, USA). The Hi-C library was evaluated by the distribution of fragment sizes with TapeStation D1000 (Agilent Technologies, Santa Clara, CA, USA) and sequenced in Illumina NovaSeq 6000 (Illumina) with the length of 150-bp paired-end reads. All of the obtained reads were quality controlled by trimming adaptor sequences and low-quality reads using Trimmomatic v0.39 [36] for Illumina reads and Porechop v0.2.4 [37] (-q 7) and NanoFilt [38] (-k 5000) for Nanopore reads.

### Genome size estimation

The quality-controlled Illumina sequencing data were used for the calculation of the genome size. Using the reads, a *k*-mer map was constructed to evaluate genome size, unique sequence ratio, and heterozygosity. For this, jellyfish v2.3.0 (Jellyfish, RRID:SCR_005491) [39] was first used to compute the distribution of the 21-mer frequencies. The final 21-mer count distribution per genome was used within the GenomeScope 2.0 [40].

### Genome assembly and scaffolding with Hi-C data

Multiple approaches were tried but the best assembly was obtained in combination of NextDenovo [41], NextPolish [42], and 3D-DNA [11]. We utilized NextDenovo v2.4.0 to assemble the *P. borealis* genome using only the Nanopore long reads. After the assembly, we applied the Illumina short reads to polish the assembled contigs by operating NextPolish v1.1.0. All software parameter settings were default.

To obtain a chromosome-level genome assembly of *P. borealis*, we employed the Hi-C technology to scaffold assembled contigs. Detailed procedures are as follows. (i) The paired-end Illumina reads were mapped onto the polished assembly using HiC-Pro v3.0.0 (HiC-Pro, RRID:SCR_017643) [43] with default parameters to check the quality of the raw Hi-C reads. (ii) Juicer v1.6 (Juicer, RRID:SCR_017226) [44] and 3D-DNA v180419 [11] were applied to cluster the genomic contig sequences into potential chromosomal groups. (iii) Juicebox v1.13.01 (Juicebox, RRID:SCR_021172) [45] was used to validate the contig orientation and to remove ambiguous fragments with the assistance of manual correction.

### Assessment of the chromosome-level genome assembly

Two routine methods were employed to assess the completeness of our finally assembled genome as follows. (i) BUSCO v5.2.2 (BUSCO, RRID:SCR_015008) [12] assessment: The metazoan_odb10 and eukaryotic_odb10 orthologues were used as the BUSCO reference. (ii) QV score and error rate were estimated with Merqury v1.3 [13].

### RNA extraction and sequencing

Total RNA was isolated using TRIzol Reagent (Invitrogen) from 3 tissues of the same *P. borealis* digestive gland, stomach, and epidermis of the arm following the manufacturer's protocol. Total RNA concentration was determined using the Quant-iT™ RNA Assay Kits (Invitrogen), and absorbance at 260 nm and 280 nm (A260/A280) was measured in the Synergy HTX Multi-Mode microplate reader (Biotek). Their quality was verified by gel electrophoresis. Messenger RNA (mRNA) was isolated using the Magnosphere™ UltraPure mRNA purification kit (Takara) according to the manufacturer's instructions.

The complementary DNA (cDNA) library was prepared using the cDNA-PCR Sequencing Kit (SQK-PCS109, Oxford Nanopore Technologies) with the PCR Barcoding Kit (SQK-PBK004, Oxford Nanopore Technologies) in accordance with the manufacturer's protocol. In brief, reverse transcriptase (RT) and strand-switching primers were provided by ONT with the SQK-PCS109 kit. Following RT, polymerase chain reaction (PCR) amplification was performed using the LongAmpTaq 2X Master Mix (New England Biolabs), and AMpure XP beads (Beckman Coulter) were used for DNA purification. The PCR product was then subjected to ONT adaptor ligation using the SQK-PBK004. The final library was loaded onto the MinION flow cell (FLO-MIN106 and FLO-MIN111, R9.4 and R10.3) (Oxford Nanopore Technologies), and sequencing was performed on a MinION MK1b and MinKNOW software (19.10.1).

We also used an Illumina platform to generate short high-quality sequencing reads. Using Truseq Stranded mRNA Prep kit, we constructed the cDNA library. After evaluating the distribution of fragment sizes with BioAnalyzer 2100 (Agilent Technologies), it was sequenced in the Illumina NovaSeq 6000 (Illumina) with the length of 100-bp paired-end reads.

### Hybrid assembly of transcriptome

To assemble the transcriptome, we selected a hybrid approach to restore more known genes and discover alternatively spliced isoforms, which can be useful in transcriptome analysis of a previously unsequenced organism. Therefore, long reads and short reads from 3 tissues were used for assembly. To ensure the accuracy of subsequent analyses, we trimmed the raw reads to remove adaptor sequences and low-quality reads. Trimmomatic v0.39 (Trimmomatic, RRID:SCR_011848) and Porechop v0.2.4 (Porechop, RRID:SCR_016967) were used to trim reads for Illumina and Nanopore reads, respectively. Subsequently, the clean reads were assembled using rnaSPAdes v3.14.1 (rnaSPAdes, RRID:SCR_016992) [46] with default parameters and open reading frames with at least 100 amino acids extracted from transcripts using TransDecoder (TransDecoder, RRID:SCR_017647) [47].

### Annotation of repetitive elements

Repetitive elements in the final assembly were annotated using the following 2 different strategies: (i) *de novo*annotation: RepeatModeler v2.0.1 (RepeatModeler, RRID:SCR_015027) [48] and LTR_Finder v2.0.1 (LTR_Finder, RRID:SCR_015247) [49] were used to build a local repeat reference. Subsequently, the genome assembly was aligned with this reference to annotate the *de novo* predicted repeat elements using RepeatMasker v4.1.1 (RepeatMasker, RRID:SCR_012954) [50]. (ii) Homology annotation: our genome assembly was searched in the RepBase (RepeatMaskerEdition) [51] using RepeatMasker v4.1.1. Finally, these data from the 2 strategies were integrated to generate a nonredundant data set of repetitive elements in the final *P. borealis* genome assembly.

### Gene prediction and function annotation

Three methods were used to predict the *P. borealis* gene set from the soft masked *P. borealis* genome. (i) *Ab initio* gene prediction: Augustus v3.4.0 (Augustus, RRID:SCR_008417) [52, 53], GeneMark-ET v3.62 [54], Braker v2.1.5 (BRAKER, RRID:SCR_018964) [55–59], and SNAP v2.51.7 [60] were employed to annotate gene models. (ii) Evidence-based gene prediction: Exonerate (Exonerate, RRID:SCR_016088) [61] was utilized to annotate gene models with expressed sequence tag (EST) and protein homology data set. Assembled transcriptome of *P. borealis* was used for the EST data set and protein sequences of *A. rubens* (GCF_902459465.1) from NCBI were used for protein homology data set. (iii) Consensus gene prediction: EVidenceModeler (EVidenceModeler, RRID:SCR_014659) [62] (EVM) combined predicted *ab initio* gene models and evidence-based gene models into weighed consensus gene structures. This predicted gene set was searched in 3 public functional databases, including NCBI Nr (nonredundant protein sequences), Swiss-Prot [63], and Pfam database [64], to identify the potential function and functional domains with BLATP v2.10.0+ [65] and Interproscan5 [66].

### Gene family expansion and contraction

We downloaded the protein sets of 6 echinoderm species, *A. rubens* (GCF_902459465.1), *A. planci* (GCF_001949145.1), *P. miniata* (GCF_015706575.1), *L. variegatus* (Lvar2.2), *P. parvimensis* (Pparv_v1.0), and *S. purpuratus* (GCF_000002235.5) from NBCI and EchinoBase [67] to analyze the phylogenetic tree and identify the one-to-one orthologous proteins within the 7 examined species through OrthoFinder v2.5.2 (OrthoFinder, RRID:SCR_017118) [68]. Species tree from OrthoFinder was used to show the phylogenetic relationship. Regarding the tree, we used CAFE5 (CAFE, RRID:SCR_005983) [16] to detect gene family expansion and contraction in the assembled *P. borealis* genome with default parameters. GO enrichment using EnrichGO (clusterProfiler v4.0.4) [69] was derived with the Fisher exact test and chi-square test and then adjusted using the Benjamini–Hochberg procedure.

### Genes under positive selection

Positively selected genes in the *P. borealis* genome were detected from one-to-one orthologous genes, in which the *P. borealis* was used as the foreground branch, and *A. rubens, A. planci, P. miniata, L. variegatus, P. parvimensis*, and *S. purpuratus* were used as the background branches. To detect positively selected genes, we used BLASTP v2.10.0+ (BLASTP, RRID:SCR_001010) to screen out 115 one-to-one orthologous genes among 7 species. The multiple alignment was performed by the GUIDANCE v2.02 software (-msaProgram CLUSTALW, -seqType aa) [70–72] and PAL2NAL v14 [73] was applied to convert protein sequence alignments into the corresponding codon alignments. The branch-site model A incorporated in the PAML package (v4.9j) [74] was employed to detect positively selected genes. The null model used in the branch-site test (model = 2, NSsites = 2, fix_omega = 1, omega = 1) assumed that the comparison of the substitution rates at nonsynonymous and synonymous sites (Ka/Ks ratio) for all codons in all branches must be ≤ 1, whereas the alternative model (model = 2, NSsites = 2, fix_omega = 0) assumed that the foreground branch included codons evolving at Ka/Ks >1. A maximum likelihood ratio test was used to compare the 2 models. *P*-values were calculated through the chi-square distribution with 1 degree of freedom (*df* = 1). The *P*-values were then adjusted for multiple testing using the false discovery rate (FDR) method. Genes were identified as positively selected when the FDR was <0.05. Furthermore, we required that at least one amino acid site possessed a high probability of being positively selected (Bayes probability >95%). If none of the amino acids passed this cutoff in the positively selected gene, then these genes were identified as false positives and excluded. GO enrichment using EnrichGO (clusterProfiler v4.0.4) [69] was derived with the Fisher exact test and chi-square test and then adjusted using the Benjamini–Hochberg procedure with a cutoff set at *P* < 0.05.

## Data Availability

The final genome assembly and raw data from the Nanopore, Illumina, and Hi-C libraries have been deposited at NCBI under BioProject PRJNA776097. Other supporting data sets are available in the *GigaScience* database GigaDB [76].

## Abbreviations

BLAST: Basic Local Alignment Search Tool; bp: base pairs; BUSCO: Benchmarking Universal Single-Copy Orthologs; FDR: false discovery rate; Gb: Giga base pairs; GC: guanine-cytosine; GO: Gene Ontology; LINE: long interspersed nuclear element; LTR: long terminal repeat; Mb: Mega base pairs; NCBI: National Center for Biotechnology Information; NR: NCBI's nonredundant database; ONT: Oxford Nanopore Technologies; QV: quality value; SINE: short interspersed nuclear elements.

## Additional Files

**Supplementary Fig. S1**. Genome size estimation.

**Supplementary Fig. S2**. *Plazaster borealis* genome assembly completeness. (A) Hi-C interactions among 22 chromosomes. (B) Cumulative length of assembly contained within scaffolds.

**Supplementary Table S1**. Statistics of raw sequencing data.

**Supplementary Table S2**. Statistics of *Plazaster borealis* genome assembly before scaffolding.

**Supplementary Table S3**. GO and KEGG enrichment analysis of expanded and contracted gene families of 7 echinoderm species.

**Supplementary Table S4**. GO and KEGG enrichment analysis of *Plazaster borealis* specific orthologs.

**Supplementary Table S5**. GO and KEGG enrichment analysis of positively selected genes.

giac063_GIGA-D-21-00378_Original_Submission

giac063_GIGA-D-21-00378_Revision_1

giac063_GIGA-D-21-00378_Revision_2

giac063_Response_to_Reviewer_Comments_Original_Submission

giac063_Response_to_Reviewer_Comments_Revision_1

giac063_Reviewer_1_Report_Original_Submission Rute R. da Fonseca -- 12/9/2021 Reviewed

giac063_Reviewer_1_Report_Revision_1 Rute R. da Fonseca -- 3/29/2022 Reviewed

giac063_Reviewer_2_Report_Original_Submission Joseph F. Ryan -- 12/21/2021 Reviewed

giac063_Reviewer_2_Report_Revision_1 Joseph F. Ryan -- 3/28/2022 Reviewed

giac063_Supplemental_Files

## Competing Interests

The authors declare that they have no competing interests.

## Funding

This work was supported by a grant from the National Institute of Biological Resources (NIBR), funded by the Ministry of Environment (MOE) of the Republic of Korea (NIBR201930201) to J. Yu.

## Authors’ Contributions

J.Y., J.P., and S.K. conceived the project; C.B. collected the sample; B.G. performed laboratory experiments; Y.L. and B.K. constructed the assembly; Y.L. annotated the assembly; Y.L. and J.J. performed comparative genome analysis; and Y.L., B.G., and S.J. wrote the manuscript with input from all authors.
