# Supplementary material for: Chromosome-level genome assembly of Plazaster borealis sheds light on the morphogenesis of multiarmed starfish and its regenerative capacity
Source: Gigascience. 2022 Jul 9;11:giac063. doi: 10.1093/gigascience/giac063 (PMC9270726; doi:10.1093/gigascience/giac063)
Supplement: giac063_GIGA-D-21-00378_Original_Submission [file giac063_giga-d-21-00378_original_submission.pdf]

GigaScience

Chromosome-level genome assembly of Plazaster borealis: shed light on the morphogenesis of multi-armed starfish and its regenerative capacity

--Manuscript Draft--

**Manuscript Number:**	GIGA-D-21-00378
**Full Title:**	Chromosome-level genome assembly of Plazaster borealis: shed light on the morphogenesis of multi-armed starfish and its regenerative capacity
**Article Type:**	Data Note
**Funding Information:**	National Institute of Biological Resources (NIBR201930201) — PhD Jaewoong Yu
**Abstract:**	Background: Plazaster borealis has a unique morphology, multiple arms with clear distinction between disk and arms, that does not follow remarkable characteristic of Echinoderm, penta-radial symmetry. Though it seems to be related with accelerated evolutionary rate at the morphological level, the lack of a reference genome has limited further research in-depth. Herein we report the first chromosome-level reference genome of P. borealis .<br>Findings: High coverage of long and short read data was used to assemble de novo 561Mb reference genome of P. borealis and Hi-C sequencing data was used to scaffold into 22 chromosomal scaffolds comprising 92.38% of the genome. The genome showed the presence of 98.8% of complete eukaryote BUSCO and 98.0% of complete metazoan BUSCO, indicating high quality assembly. Through the comparative genome analysis, we identified evolutionary accelerated genes related with morphogenesis and regeneration, suggesting their potential role in shaping body pattern and capacity of regeneration.<br>Conclusion: This first chromosome-level genome assembly of P. borealis provides fundamental insights into echinoderm biology, as well as the genomic mechanism underlying its unique morphology and regeneration.
**Corresponding Author:**	Jaewoong Yu<br>eGnome Inc<br>Seoul, KOREA, REPUBLIC OF
**Corresponding Author Secondary Information:**	
**Corresponding Author's Institution:**	eGnome Inc
**Corresponding Author's Secondary Institution:**	
**First Author:**	Yujung Lee
**First Author Secondary Information:**	
**Order of Authors:**	Yujung Lee
	Bongsang Kim
	Jaehoon Jung
	Bomin Koh
	So Yun Jhang
	Chaeyoung Ban
	Won-Jae Chi
	Soonok Kim
	Jaewoong Yu
**Order of Authors Secondary Information:**	
**Additional Information:**	

Powered by Editorial Manager® and ProduXion Manager® from Aries Systems Corporation

Question	Response
Are you submitting this manuscript to a special series or article collection?	No
**Experimental design and statistics**<br><br>Full details of the experimental design and statistical methods used should be given in the Methods section, as detailed in our Minimum Standards Reporting Checklist. Information essential to interpreting the data presented should be made available in the figure legends.<br><br>Have you included all the information requested in your manuscript?	Yes
**Resources**<br><br>A description of all resources used, including antibodies, cell lines, animals and software tools, with enough information to allow them to be uniquely identified, should be included in the Methods section. Authors are strongly encouraged to cite Research Resource Identifiers (RRIDs) for antibodies, model organisms and tools, where possible.<br><br>Have you included the information requested as detailed in our Minimum Standards Reporting Checklist?	Yes
**Availability of data and materials**<br><br>All datasets and code on which the conclusions of the paper rely must be either included in your submission or deposited in publicly available repositories (where available and ethically appropriate), referencing such data using a unique identifier in the references and in the "Availability of Data and Materials" section of your manuscript.	Yes

Have you have met the above requirement as detailed in our Minimum Standards Reporting Checklist?	

Manuscript
Click here to access/download;Manuscript;Manuscript_ver2.docx

Chromosome-level genome assembly of *Plazaster borealis*: shed light on the morphogenesis of multi-armed starfish and its regenerative capacity

Yujung Lee[1] [0000-0003-2279-3147]; Bongsang Kim[1,2] [0000-0001-7526-8421]; Jaehoon Jung[1,2] [0000-0003-2019-0895]; Bomin Koh[1] [0000-0001-6702-6449]; So Yun Jhang[1,3] [0000-0002-2152-3746]; Chaeyoung Ban[1] [0000-0003-4566-4313]; Won-Jae Chi[4] [0000-0003-2893-7930]; Soonok Kim[4] [0000-0003-1654-3643]; Jaewoong Yu[1,*] [0000-0002-4120-8890];

[1]eGnome, Inc., 26 Beobwon-ro 9-gil, Sonpa-gu, Seoul 05836, Republic of Korea;

[2]Department of Agricultural and Life Sciences and Research Institute of Population Genomics, Seoul National University, Seoul, Republic of Korea;

[3]Interdisciplinary Program in Bioinformatics, Seoul National University, Seoul, 151-742, Republic of Korea;

[4]Microorganism Resources Division, National Institute of Biological Resources, Incheon 22689, Republic of Korea;

*Correspondence address: Jaewoong Yu, eGnome Inc., 26 Beobwon-ro 9-gil, Sonpa-gu, Seoul 05836, Korea. Email: jwyu@egnome.co.kr; Tel.: +82-070-4694-6355

Email addresses/ ORCIDs

Yujung Lee[1]: lyjung711@gmail.com, lyjung7@egnome.co.kr/ 0000-0003-2279-3147

Bongsang Kim[1,2]: babybird93@snu.ac.kr, kimbongsang@egnome.co.kr / 0000-0001-7526-8421

Jaehoon Jung[1,2]: motto@snu.ac.kr, motto@egnome.co.kr / 0000-0003-2019-0895

Bomin Koh[1]: chloekoh@egnome.co.kr / 0000-0001-6702-6449

So Yun Jhang[1,3]: soyun4595@snu.ac.kr, soyun4595@egnome.co.kr / 0000-0002-2152-3746

Chaeyoung Ban[1]: terryban@egnome.co.kr / 0000-0003-4566-4313

Won-Jae Chi3[4]: wjchi76@korea.kr / 0000-0003-2893-7930

Soonok Kim[4]: sokim90@korea.kr / 0000-0003-1654-3643

Jaewoong Yu[1,*]: jwyu@egnome.co.kr / 0000-0002-4120-8890

Abstract

Background: *Plazaster borealis* has a unique morphology, multiple arms with clear distinction between disk and arms, that does not follow remarkable characteristic of Echinoderm, penta-radial symmetry. Though it seems to be related with accelerated evolutionary rate at the morphological level, the lack of a reference genome has limited further research in-depth. Herein we report the first chromosome-level reference genome of *P. borealis*.

Findings: High coverage of long and short read data was used to assemble de novo 561Mb reference genome of *P. borealis* and Hi-C sequencing data was used to scaffold into 22 chromosomal scaffolds comprising 92.38% of the genome. The genome showed the presence of 98.8% of complete eukaryote BUSCO and 98.0% of complete metazoan BUSCO, indicating high quality assembly. Through the comparative genome analysis, we identified evolutionary accelerated genes related with morphogenesis and regeneration, suggesting their potential role in shaping body pattern and capacity of regeneration.

Conclusion: This first chromosome-level genome assembly of *P. borealis* provides fundamental insights into echinoderm biology, as well as the genomic mechanism underlying its unique morphology and regeneration.

Data Description

Context

Echinoderms are various invertebrate marine animals that belong to the phylum Echinodermata. They have three remarkable characteristics: 1) extensive regenerative abilities in both adult and larval forms [1, 2], 2) the water vascular system used for various aspects of animal life [3], 3) extraordinary morphological characteristics, penta-radial symmetry [4, 5].

Penta-radial symmetry was observed in all extant classes of echinoderm. Echinoids (sea urchin) and holothurians (sea cucumber) always have five ambulacral grooves, and crinoids have many arms in multiples of five that branch out from the five primary brachia [4, 5]. Most species of asteroids and ophiuroids are five-armed, but many exceptions are scattered across both time and taxa. Extant asteroids are distinguished by 34 families, including 20 families of only five-armed species, nine of both five-armed and multi-armed species, and five families with exclusively multi-armed species [6]. However, most multi-armed forms have arm numbers that cannot be divided into five, raising questions about the apparent morphology of echinoderm, penta-radial symmetry.

Plazaster borealis, octopus starfish, is a starfish inhabit in the water surrounded by Korea and Japan [7, 8] and belongs to the family *Labidiasteridae*, one of five exclusively multi-armed families [6]. As in figure 1A, it has unique morphology, the number of arms is around 31~40, which is a large number among starfishes, and it shows a clear differentiation between arms and disk [9].

In the previous study of *P. borealis*, Matsuoka investigated the molecular phylogenetic relationship of five species from the order Forcipulatida: *Asterias amurensis*, *Aphelasterias japonica*, *Distolasterias nipon*, *Coscinasterias acutispina*, and *Plazaster borealis* [10]. *P. borealis* was the most closely related with five armed *A. amurensis* and distantly related with multi-armed *C. acutispina*. The result suggested that the unique morphology of *P. borealis* might have differentiated from the starfish with standard morphology: five arms and no distinction of disk and arm. Furthermore, it shows the possibility of an accelerated evolutionary rate at the morphological level in *P. borealis*. However, the absence of reference genome limited advanced research in depth. To understand the genetic basis of the specialized morphology of the starfish, we sequenced the genome of *P. borealis* and carried out

comparative genomic analyses with the high-quality and well-annotated genome sequences of other six echinoderms (*Asterias rubens, Acanthaster planci*, *Patiria miniata*, *Lytechinus variegatus*, *Parastichopus parvimensis*, and *Strongylocentrotus purpuratus*).

Chromosome-level genome assembly of the octopus starfish

We estimated the genome size of *P. borealis* with GenomeScope to be ~497Mb (Supplementary Figure 1). Based on this estimation, a comprehensive sequencing data set was generated for the *P. borealis* genome assembly. From the Nanopore sequencing platform, a total of 57.76 Gb long read was yielded with 116x coverage. Using the Illumina sequencing platform, 142x coverage of Illumina short paired-end read sequencing data and 115x coverage of Hi-C paired-end reads was generated (Supplementary Table 1). Moreover, we sequenced 25.63 Gb of RNA Illumina short paired-end reads and 7.28 Gb of RNA Nanopore long reads to construct transcriptome assembly utilized for annotation.

A total 561Mb of draft *P. borealis* genome was assembled into 179 contigs with N50 of 11Mb (Supplementary Table 2). To obtain chromosomal information, we scaffolded the contigs using 3D-DNA [11]. The total size of the final assembly was 561Mb comprising 22 chromosome-level scaffolds with a contig N50 of 24Mb. These 22 chromosome-level scaffolds comprise 92.48% of the assembly, although the remaining 42 Mb were unanchored and required further investigation (Table 1, Supplementary Figure 2). This number is consistent with chromosome results of other species of the order Forcipulatida, further supporting the accurate chromosome number acquired in the current study.

Completeness of the assembled genome

The genome completeness was evaluated using BUSCO with the eukaryotic_odb10 and metazoan_odb10. Total of 252 (98.8%) core eukaryote genes and 935 (98.0%) core metazoan genes were successfully detected in the genome. Each gene sets consisted as following, S: 97.6%, 97.0%; D: 1.2%, 1.0%; F: 0.8%, 1.2%; and M: 0.4%, 0.8%. (S: single-copy, D: duplicated, F: fragmental, M: missed of eukaryotic_odb10 and metabozan_odb10 data set, respectively). We also estimated the overall assembly quality by comparing the k-mer distribution of the assemblies and the Illumina short read sets using Merqury. The genome assembly of *P. borealis* showed high-quality values (QV > 36) with an error rate of 0.00023 (Table 1). Additionally, the GC content of *P. borealis* was 38.89%, which was very similar to that of *A. rubens* (38.76%) and *P. ochraceus* (39.01%), the species of the order Forcipulatida. The assessment results validated the high quality of our final genome assembly. To our knowledge, this is the first high-quality chromosome level genome assembly for *P. borealis* and the first reference genome of the family *Labidiasteridae*.

Annotation of repeats and genes

Repetitive elements accounted for 51.05% of the whole genome assembly, and detailed percentages of the predominant repetitive element families are summarized in Table 2. We annotated a total of 26,836 genes onto the assembled regions. Compared to other starfish, *P. borealis* has a similar average exon length (213 bp) and exon number per gene (7.19), but it has a shorter intron length (1,261 bp) than *A. rubens*. BUSCO benchmarking value of this gene set was summarized as C: 92.6%, including S: 90.0%, D: 2.6%, F:4.6%, M: 2.8% (S: single-copy, D: duplicated, F: fragmental, M: missed of metabozan_odb10 data set). Following a standard functional annotation, we observed that 24,248 (96.13%) genes were successfully annotated with at least one related functional assignment (Table 3).

Phylogenetic and syntenic relationship

To understand the phylogenetic location of *P. borealis*, we used a BLAST-based hierarchical clustering algorithm for genome-wide phylogenetic analysis based on protein sequences from seven echinoderm genomes. *P. borealis* was the most closely related to *A. rubens* (Figure 2), consistent with both previous results and taxa [10].

Syntenic relationships analyzed by MCscan [12] also proved their relationship. In the genome of *P. borealis* and *A. rubens*, every chromosome matched each other well that entire chromosomes seem to be highly conserved, but an expansion was detected in chromosome 7 of *P. borealis* (Figure 3A, 3B). A similar tendency, using Chromeister [13], was observed with *P. ochraceus* and *M. glacialis*, other species of the order Forcipulatida. Among three genomes, *A. rubens*, *P. ochraceus*, and *M. glacialis*, *P. ochraceus* was the most conserved with *P. borealis* with a score of 0.301, which seems to be influenced by the observed expansion. These results suggest that genomes within the Forcipulatida order are remarkably conserved in terms of synteny and chromosome, supporting the high quality of the assembled genome.

Gene family evolution in *P. borealis*

Based on the assumption that the unique morphology of *P. borealis* is explained by accelerated evolutionary rate [10], we performed comparative genomic analyses among seven echinoderm species. As a result, a total of 24,074 families of homologous genes were detected, including 3,864 gene families that commonly existed in the seven species and 5,382 gene families that existed only in starfishes. In addition, we identified 607 gene families unique in *P. borealis*

consisting of 2,631 genes and 111 one-to-one orthologous genes between *P. borealis* and six other species.

Although the genetic mechanism underlying the development of supernumerary arms of starfish is elusive, it is expected that genes related to tissue morphogenesis are increased to produce excessive arms. To investigate the expanded gene families, we performed expansion and contraction analysis of gene families using CAFE. Compared with six echinoderm species, 286 gene families were expanded, whereas 2,072 gene families were contracted in *P. borealis* (Figure 2). The significantly expanded genes in the genome of *P. borealis* were significantly enriched in categories of Notch and BMP signaling pathway, body pattern specification, morphogenesis, and eye development ($P<0.02$) (Figure 4). Collectively, these expanded gene families are likely to play an enhanced role in forming supernumerary arms of *P. borealis*. It is generally accepted that Notch and BMP signaling are evolutionally conserved and play multiple roles during animal development, especially in regulating body patterns. The Notch signaling pathway is essential for cell proliferation, cell fate decisions, and induction of differentiation during embryonic and postnatal development [14-16]. Besides regulating cell-fate decisions at an individual cell level, cell to cell signaling mechanism of Notch coordinates the spatiotemporal patterning in a tissue [17]. In Drosophila melanogaster, Notch functions as it is required to specify the fate of the cells that will eventually segment leg and develop leg joint [18, 19]. The mechanisms of BMP gradient formation have been studied in various animals. BMP2/4 signaling study of sea urchin showed that interaction between BMP2/4 and chordin formed the dorsal-ventral gradient and resulted in dorsal-ventral axis patterning [20]. Furthermore, as the physical characteristic of starfish, their eyes exist on the end of each arm that denotes the development of arm is accompanied with the development of eye. However, contracted gene families of *P. borealis* had no significantly enriched functions, except GTPase regulator activity (GO:0030695, p-value=0.005647). Gene repertories of *P. borealis* showed

differences in the contents of other species' expanded and contracted genes mainly enriched in terms related to nerve development (Supplementary Table 3).

We also found gene families unique in *P. borealis* are enriched for the following gene ontology (GO) terms: apoptotic cell clearance, positive regulation of epithelial cell proliferation, vascular transport, and activation of JNKK activity (Supplementary Table 4). The enriched term, activation of JNKK activity, is involved in the JNK pathway, which promotes apoptosis by upregulating pro-apoptotic gene expression [21]. Typically, cell proliferation and death are important to achieve shaping tissue which involves changes in cell number, size, shape, and position [22]. Based on these findings, the existent of further genes of the Notch pathway, BMP pathway and JNK pathway involved in body pattern specification, cell proliferation, and apoptosis could indicate enhanced tissue shaping to form many arms.

The signaling pathways detected through expanded gene families, especially the Notch and BMP pathway, also play several key conserved roles in the regeneration of many species. For example, in the study of brittle stars, the inhibition of Notch signaling hindered arm regeneration and downregulated genes related to ECM component, cell proliferation, apoptosis and innate immunity, which are biological processes associated with regeneration [23]. In addition, previous studies of echinoderm gene expression and other animals showed that Notch and BMP signaling is the principal pathway for tissue regeneration [24, 25].

The studies of the metamorphosis of multi-armed starfishes led to propose the 'Five-Plus' hypothesis [6, 26]. It states that five primary arms generated concurrently develop in a controlled unit and supernumerary arms are produced in the separate and independent pathways. Although still these pathways are uncertain, Hotchkiss suggested two possibilities, post-generation of arms in the incompletely developed starfish or intercalated regeneration of arms in the adult [6]. The capacity of regeneration is a remarkable feature of all extant classes of

echinoderms [2]. Thus, it is possible that multi-armed starfishes could transform from five-rayed forms to multi-rayed forms by growing new arms through regeneration related mechanisms. Thus, suggesting that genes in these families may play key roles in the biosynthesis and metabolism processes of its unique body plan as well as in regeneration processes.

Using *P. borealis* as the foreground branch, six other echinoderm species as the background branches, we incorporated the branch-site model in the PAML package to detect positively selected genes. A total of 14 genes were positively selected in *P. borealis* ($p<0.05$, BEB > 0.95), and significantly enriched in GO terms related to “lipid metabolism”, “transport of proton”, “pyruvate metabolism”, and “Hedgehog signaling pathway” (Figure 5, Supplementary Table 5). It is worth noting that these positively selected genes also included BMP4, which regulates regeneration and tissue specification (Table 4).

Regeneration is a high-energy-required process that starfishes in the regeneration state increase the amount of lipid and energy in the pyloric caeca to use [27]. GPR161 and BMP4 known to be critical in regeneration were also detected as positively selected genes. The G-protein coupled receptor Gpr161 negatively regulates the Hedgehog pathway via cAMP signaling, which is known to participate tissue regeneration process [28, 29]. Additionally, previous studies of planarian regeneration indicate that BMP4 is key for tissue specification, especially dorsal-ventral polarity, which may explain the distinctive disk of *P. borealis* [30]. Together with those of previous studies, our results further suggest that related genes may have contributed to the regeneration and development of the unique body plan of *P. borealis*, multiple arms. Therefore, *P. borealis* can be potentially regarded as a valuable model to investigate the mechanisms underlying supernumerary arm development and regeneration. We believe that

this high-quality genome will supply a useful and valuable genetic resource for future research especially in unique body plan and regeneration biology.

Conclusion

The first chromosome-level *P. borealis* genome was assembled and annotated. Twenty-two chromosomal scaffolds are constructed with N50 of 24.97 Mb which showed high conservation with genomes of three starfish species of the order Forcipulatida. Furthermore, in our study, we identified the accelerated evolution of *P. borealis* in the context of genomics, which may explain its multi-armed morphology and regenerative capacity. The availability of the high-quality genome sequence of *P. borealis* is expected to provide many insights into the unique morphology of multi-armed starfish and their regeneration. Regarding the scientific value of *P. borealis,* the genome and gene inventory resulting from this study will be helpful in future research on these critical topics.

Methods

Sampling and genomic DNA extraction

Adult specimens of *P. borealis* were sampled at a depth of 31 meters near Ulleung island, Korea (latitude: 37.53390, longtitude: 130.93920) (Figure 1A). *P. borealis* was dissected with scissors to obtain gonad, pyloric caecae, stomach and epidermis of arm. Isolated tissues were frozen on dry ice immediately and kept at -80℃ until further processing. The frozen tissue was ground into a fine powder with liquid nitrogen using a pestle and mortar for the nucleic acid extraction.

High molecular weight (HMW) DNA was obtained from gonad following a nuclei isolation

method [31]. Genomic DNA was obtained from gonad following modified CTAB protocol [32] in the presence of 2% PVP (1% of MW 10,000 and 1% of MW 40,000) PolyVinylPyrrolidone (Sigma-Aldrich, Burlington, MA, USA). DNA concentration was determined using the Quant-iT PicoGreen® assay (Invitrogen, Waltham, MA, USA) and the absorbance at 260 nm and 230nm (A260/A230) was measured in the Synergy HTX Multi-Mode microplate reader (Biotek, Rochester, VT, USA). Their quality verified by gel electrophoresis.

High-throughput sequencing of genomic DNA

For Nanopore sequencing, short genomic fragments (<10 kb) were removed using a Short Read Eliminator Kit (Circulomics, Baltimore, MD, USA). The library was prepared using the ONT 1D ligation Sequencing kit (SQK-LSK109, Oxford Nanopore Technologies, Oxford, UK) with the native barcoding expansion kit (EXP-NBD104) in accordance with the manufacturer's protocol. In brief, genomic DNA was repaired using the NEBNext FFPE DNA Repair Mix (New England BioLabs, Ipswich, MA, USA) and NEBNext Ultra II End Repair/dA-Tailing Module. The end-prepped DNA was individually barcoded with ONT native barcode by NEB Blunt/TA Ligase Master Mix (New England BioLabs). Barcoded DNA samples were pooled in equal molar amounts. It was ligated with adapter using the NEBNext Quick Ligation Module (New England BioLabs). After every enzyme reaction, the DNA samples were purified using AMPure XP beads (Beckman Coulter, Brea, CA, USA). The final library was loaded onto MinION flow cell (FLO-MIN106 and FLO-MIN111, R9.4 and R10.3) (Oxford Nanopore Technologies) and PromethION flowcell(FLO-PRO002) (Oxford Nanopore Technologies). Sequencing was performed on a MinION MK1b and PromethION sequencer with MinKNOW software (19.10.1).

We also used an Illumina platform to generate short high-quality sequencing reads. DNA library was prepared using TruSeq DNA PCR-Free (Illumina, San Diego, CA, USA) and

evaluated the distribution of fragment sizes with TapeStation D1000 (Agilent Technologies, Santa Clara, CA, USA). Finally, DNA library was sequenced in the Illumina NovaSeq 6000 (Illumina) with the length of 150 bp paired-end reads.

Hi-C technology was also employed for chromosome-level genome assembly. Hi-C library construction protocol is as follows. Ground gonad tissue was mixed with 1% formaldehyde for fixing chromatin then the nuclei was isolated following a nuclei isolation method [1]. Fixed chromatin was digested with HindII-HF (New England BioLabs), the 5’ overhangs filled in with nucleotides and biotin-14-dCTP(Invitrogen) and ligated free blunt ends. After ligation, the DNA purified and removed biotin from un-Ligated DNA ends. Fragmentation and size selection was performed to shear the Hi-C DNA. Hi-C Library preparation is performed using ThruPLEX® DNA-seq Kit (Takara Bio USA, Inc, Mountain View, CA, USA). HI-C library was evaluated the distribution of fragment sizes with TapeStation D1000 (Agilent Technologies, Santa Clara, CA, USA). HI-C library was sequenced in the Illumina NovaSeq 6000 (Illumina) with the length of 150 bp paired-end reads. All of the obtained reads were quality controlled by trimming adaptor sequences and low-quality reads using Trimmomatic v0.39 [33] for Illumina reads and Porechop v0.2.4 [34] (-q 7) and NanoFilt [35] (-k 5000) for Nanopore reads.

Genome size estimation

The quality controlled Illumina sequencing data was used for the calculation of the genome size. Using the reads, a k-mer map was constructed to evaluate genome size, unique sequence ratio, and heterozygosity. For this, jellyfish v2.3.0 [36] was first used to compute the distribution of the 21-mer frequencies. The final 21-mer count distribution per genome was used within the GenomeScope 2.0 [37].

Genome assembly and scaffolding with Hi-C data

Multiple approaches were tried but the best assembly was obtained in combination of NextDenovo [38], NextPolish [39] and 3D-DNA [11]. We utilized NextDenovo v2.4.0 to assemble the *P. borealis* genome using only the Nanopore long reads. After the assembly, we applied the Illumina short reads to polish the assembled contigs by operating NextPolish v1.1.0. All software parameter setting were default.

To obtain a chromosome-level genome assembly of *P. borealis*, we employed the Hi-C technology to scaffold assembled contigs. Detailed procedures are as follows. (i) The paired-end Illumina reads were mapped onto the polished assembly using HiC-Pro v3.0.0 [40] with default parameters to check the quality of the raw Hi-C reads. (ii) Juicer v1.6 [41] and 3D-DNA v180419 [11] were applied to cluster the genomic contig sequences into potential chromosomal groups. (iii) Juicebox v1.13.01 [42] was used to validate the contig orientation and to remove ambiguous fragments with the assistance of manual correction.

Assessment of the chromosome-level genome assembly

Two routine methods were employed to assess the completeness of our finally assembled genome as follows. (i) Bechmarking Universal Single-Copy Orthologues (BUSCO) v5.2.2 [43] assessment: The metazoan_odb10 and eukaryotic_odb10 orthologues were used as the BUSCO reference. (ii) QV score and error rate was estimated with Merqury v1.3 [44].

RNA extraction and sequencing

Total RNA was isolated using TRIzol Reagent(Invitrogen) from three tissues of same *P. borealis*, digestive gland, stomach and epidermis of arm following the manufacturer’s protocol. Total RNA concentration was determined using the Quant-iT™ RNA Assay Kits (Invitrogen) and the absorbance at 260 nm and 280 nm (A260/A280) was measured in the Synergy HTX Multi-Mode microplate reader (Biotek). Their quality verified by gel electrophoresis. mRNA

was isolated using Magnosphere™ UltraPure mRNA purification kit(Takara) according to the manufacturer's instructions.

cDNA library was prepared using cDNA-PCR Sequencing Kit (SQK-PCS109, Oxford Nanopore Technologies) with the PCR Barcoding Kit (SQK-PBK004, Oxford Nanopore Technologies) in accordance with the manufacturer's protocol. In brief, RT and strand-switching primers were provided by ONT with the SQK-PCS109 kit. Following RT, PCR amplification was performed using the LongAmpTaq 2X Master Mix (New England Biolabs) and AMpure XP beads (Beckman Coulter) were used for DNA purification. The PCR product was then subjected to ONT adaptor ligation using the SQK-PBK004. The final library was loaded onto MinION flow cell (FLO-MIN106 and FLO-MIN111, R9.4 and R10.3) (Oxford Nanopore Technologies) and sequencing was performed on a MinION MK1b and MinKNOW software (19.10.1).

We also used an Illumina platform to generate short high-quality sequencing reads. Using Truseq Stranded mRNA Prep kit, we constructed cDNA library. After evaluating the distribution of fragment sizes with BioAnalyzer 2100 (Agilent Technologies, Santa Clara, CA, USA), it was sequenced in the Illumina NovaSeq 6000 (Illumina, San Diego, CA, USA) with the length of 100 bp paired-end reads.

Hybrid assembly of transcriptome

To assemble transcriptome, we selected hybrid approach to restore more known genes and discover alternatively spliced isoforms, which can be useful in transcriptome analysis of previously unsequenced organism. Therefore, long reads and short reads from three tissues were used for assembly. To ensure the accuracy of subsequent analyses, we trimmed the raw reads to remove adaptor sequences and low-quality reads. Trimmomatic v0.39 and Porechop v0.2.4 were used to trim reads for Illumina and Nanopore reads, respectively. Subsequently,

the clean reads were assembled using rnaSPAdes v3.14.1 [45] with default parameters and transcriptomes with at least 100 amino acids were extracted using TransDecoder [46].

Annotation of repetitive elements

Repeatitive elements in the final assembly were annotated using the following two different strategies, (i) de novo annotation: RepeatModeler v2.0.1 [47] and LTR_Finder v2.0.1 [48] were used to build a local repeat reference. Subsequently, the genome assembly was aligned with this reference to annotate the de novo predicted repeat elements using RepeatMasker v4.1.1 [49]. (ii) Homology annotation: Our genome assembly was searched in the RepBase (RepeatMaskerEdition) [50] using RepeatMasker v4.1.1. Finally, these data from the two strategies were integrated to generate a nonredundant data set of repetitive elements in the final *P. borealis* genome assembly.

Gene prediction and function annotation

Three methods were used to predict the *P. borealis* gene set from the soft masked *P. borealis* genome. (i) ab initio gene prediction: Augustus v3.4.0 [51, 52], GeneMark-ET v3.62 [53], Braker v2.1.5 [54-58] and SNAP v2.51.7 [59] were employed to annotate gene models. (ii) Evidence-based gene prediction: Exonerate [60] were utilized to annotate gene models with expressed sequence tag (EST) and protein homology dataset. Assembled transcriptome of *P. borealis* were used for EST dataset and protein sequences of *A. rubens* (GCF_902459465.1) from NCBI were used for protein homology dataset. (iii) Consensus gene prediction: EVidenceModeler [61] (EVM) combined predicted ab initio gene models and evidence based gene models into weighed consensus gene structures. This predicted gene set was searched in three public functional databases, including NCBI Nr (nonredundant protein sequences), Swiss-Prot [62] and Pfam database [63] to identify the potential function and functional domains with BLATP v2.10.0+ [64] and Interproscan5 [65].

Gene family expansion and contraction

We downloaded the protein sets of 6 echinoderm species, *Asterias rubens* (GCF_902459465.1), *Acanthaster planci* (GCF_001949145.1), *Patiria miniata* (GCF_015706575.1), *Lytechinus variegatus* (Lvar2.2), *Parastichopus parvimensis* (Pparv_v1.0), and *Strongylocentrotus purpuratus* (GCF_000002235.5) from NBCI and EchinoBase (http://www.echinobase.org) [66] to analyze phylogenetic tree and identify the one-to-one orthologous proteins within the 7 examined species through OrthoFinder v2.5.2 [67]. Regarding the phylogenetic tree, we used CAFE5 [68] to detect gene family expansion and contraction in the assembled *P. borealis* genome with default parameters. GO enrichment using EnrichGO (clusterProfiler v4.0.4) [69] was derived with the Fisher's exact test and chi-square test and then adjusted using the Benjamini-Hochberg procedure.

Genes under positive selection

Positively selected genes in the *P. borealis* genome were detected from one-to-one orthologous genes, in which the *P. borealis* was used as the foreground branch, and the *A. rubens*, *A. planci*, *P. miniata*, *L. variegatus*, *P. parvimensis* and *S. purpuratus* were used as the background branches. To detect positively selected genes, we used BLASTP v2.10.0+ to screen out 115 one-to-one orthologous genes among 7 species. The multiple alignment was performed by the GUIDANCE v2.02 software [70-72] and PAL2NAL v14 [73] was applied to convert protein sequence alignments into the corresponding codon alignments. The branch-site model incorporated in the PAML package (v4.9j) [74] was employed to detect positively selected genes. The null model used in the branch-site test assumed that the comparison of the substitution rates at nonsynonymous and synonymous sites (Ka/Ks ratio) for all codons in all branches must be <= 1, whereas the alternative model assumed that the foreground branch included codons evolving at Ka/Ks > 1. A maximum likelihood ratio test was used to compare

the two models. P-values were calculated through the chi-square distribution with 1 degree of freedom (df=1). The p-values were then adjusted for multiple testing using the false discovery rate (FDR) method. Genes were identified as positively selected when the FDR < 0.05. Furthermore, we required that at least one amino-acid site possessed a high probability of being positively selected (Bayes probability > 95%). If none of the amino acids passed this cutoff in the positively selected gene, then these genes were identified as false positives and excluded. GO enrichment using EnrichGO (clusterProfiler v4.0.4) [69] was derived with the Fisher's exact test and chi-square test and then adjusted using the Benjamini-Hochberg procedure with a cutoff set at $p < 0.05$.

Data availability

The final genome assembly and raw data from the Nanopore, Illumina and Hi-C libraries have been deposited at NCBI under BioProject PRJNA776097.

Additional Files

Supplementary Figure S1. Genome size estimation

Supplementary Figure S2. *Plazaster borealis* genome assembly completeness. (A) Hi-C interactions among 22 chromosomes. (B) Cumulative length of assembly contained within scaffolds.

Supplementary Table S1. Statistics of raw sequencing data

Supplementary Table S2. Statistics of *Plazaster borealis* genome assembly before scaffolding.

Supplementary Table S3. GO and KEGG enrichment analysis of expanded and contracted gene families of seven echinoderm species.

Supplementary Table S4. GO and KEGG enrichment analysis of *Plazaster borealis* specific orthologs.

Supplementary Table S5. GO and KEGG enrichment analysis of positively selected genes.

Competing Interests

The authors declare that they have no competing interests.

Funding

This work was supported by a grant from the National Institute of Biological Resources (NIBR), funded by the Ministry of Environment (MOE) of the Republic of Korea (NIBR201930201).

Ministry of Environment, National Institute of Biological Resources, NIBR201930201, J Yu;

Authors' Contribution

J.Y., J.P., and S.K. conceived the project; C.B. collected the sample; B.G. performed laboratory experiments; Y.L. and B.K. constructed the assembly; Y.L. annotated the assembly; Y.L. and J.J. performed comparative genome analysis; and Y.L., B.G and S.J. wrote the manuscript with input from all authors.

Acknowledgements

We thank the reviewers for their helpful comments and constructive suggestions on the manuscript. We also appreciate to the NIBR for the support.

References

1. Garcia-Arraras JE and Dolmatov IY. Echinoderms: potential model systems for studies on muscle regeneration. Curr Pharm Des. 2010;16 8:942-55. doi:10.2174/138161210790883426.
2. Carnevali MC. Regeneration in Echinoderms: repair, regrowth, cloning. 2006.
3. Sprinkle J. Patterns and problems in echinoderm evolution. Echinoderm Studies. CRC Press; 2020. p. 1-18.

4. Nichols D. Pentamerism and the Calcite Skeleton in Echinoderms. Nature. 1967;215 5101:665-6. doi:10.1038/215665a0.
5. Stephenson DG. Pentameral Symmetry in Echinoderms. Nature. 1967;216 5119:994-. doi:10.1038/216994a0.
6. Hotchkiss FHC. On the Number of Rays in Starfish1. American Zoologist. 2015;40 3:340-54. doi:10.1093/icb/40.3.340.
7. Sook S. A Systematic Study on the Asteroidea in the East Sea, Korea. Animal Systematics, Evolution and Diversity. 1995;11 2:243-63.
8. Uchida T. Report of the Biological Survey of Mutsu Bay. 11. Starfishes of Mutsu Bay. Scientific Reports of Tohoku Imperial University. 1928.
9. Hayashi R. Contributions to the Classification of the Sea-stars of Japan.: II. Forcipulata, with the Note on the Relationships between the Skeletal Structure and Respiratory Organs of the Sea-stars (With 11 Plates and 115 textfigures). 北海道帝國大學理學部紀要. 1943;8 3:133-281.
10. Matsuoka N, Fukuda K, Yoshida K, Sugawara M and Inamori M. Biochemical systematics of five asteroids of the family Asteriidae based on allozyme variation. Zoological science. 1994;11 2:p343-9.
11. Dudchenko O, Batra SS, Omer AD, Nyquist SK, Hoeger M, Durand NC, et al. De novo assembly of the Aedes aegypti genome using Hi-C yields chromosome-length scaffolds. Science. 2017;356 6333:92-5. doi:10.1126/science.aal3327.
12. Wang Y, Tang H, Debarry JD, Tan X, Li J, Wang X, et al. MCScanX: a toolkit for detection and evolutionary analysis of gene synteny and collinearity. Nucleic Acids Res. 2012;40 7:e49. doi:10.1093/nar/gkr1293.
13. Perez-Wohlfeil E, Diaz-Del-Pino S and Trelles O. Ultra-fast genome comparison for large-scale genomic experiments. Sci Rep. 2019;9 1:10274. doi:10.1038/s41598-019-46773-w.
14. Artavanis-Tsakonas S, Rand MD and Lake RJ. Notch signaling: cell fate control and signal integration in development. Science. 1999;284 5415:770-6. doi:10.1126/science.284.5415.770.
15. Lai EC. Notch signaling: control of cell communication and cell fate. Development. 2004;131 5:965-73. doi:10.1242/dev.01074.
16. Sato C, Zhao G and Ilagan MX. An overview of notch signaling in adult tissue renewal and maintenance. Curr Alzheimer Res. 2012;9 2:227-40. doi:10.2174/156720512799361600.
17. Bocci F, Onuchic JN and Jolly MK. Understanding the Principles of Pattern Formation Driven by Notch Signaling by Integrating Experiments and Theoretical Models. Front Physiol. 2020;11:929. doi:10.3389/fphys.2020.00929.
18. de Celis JF, Tyler DM, de Celis J and Bray SJ. Notch signalling mediates segmentation of the Drosophila leg. Development. 1998;125 23:4617-26.
19. Cordoba S and Estella C. Role of Notch Signaling in Leg Development in Drosophila melanogaster. Adv Exp Med Biol. 2020;1218:103-27. doi:10.1007/978-3-030-34436-8_7.
20. Lapraz F, Besnardeau L and Lepage T. Patterning of the Dorsal-Ventral Axis in Echinoderms: Insights into the Evolution of the BMP-Chordin Signaling Network. PLOS Biology. 2009;7

11:e1000248. doi:10.1371/journal.pbio.1000248.
21. Dhanasekaran DN and Reddy EP. JNK signaling in apoptosis. Oncogene. 2008;27 48:6245-51. doi:10.1038/onc.2008.301.
22. Heisenberg CP and Bellaiche Y. Forces in tissue morphogenesis and patterning. Cell. 2013;153 5:948-62. doi:10.1016/j.cell.2013.05.008.
23. Mashanov V, Akiona J, Khoury M, Ferrier J, Reid R, Machado DJ, et al. Active Notch signaling is required for arm regeneration in a brittle star. PLoS One. 2020;15 5:e0232981. doi:10.1371/journal.pone.0232981.
24. Reinardy HC, Emerson CE, Manley JM and Bodnar AG. Tissue regeneration and biomineralization in sea urchins: role of Notch signaling and presence of stem cell markers. PLoS One. 2015;10 8:e0133860. doi:10.1371/journal.pone.0133860.
25. Shao Y, Wang XB, Zhang JJ, Li ML, Wu SS, Ma XY, et al. Genome and single-cell RNA-sequencing of the earthworm Eisenia andrei identifies cellular mechanisms underlying regeneration. Nat Commun. 2020;11 1:2656. doi:10.1038/s41467-020-16454-8.
26. Frederick HCH. A "Rays-as-Appendages" Model for the Origin of Pentamerism in Echinoderms. Paleobiology. 1998;24 2:200-14.
27. Rubilar T, Villares G, Epherra L, Díaz-de-Vivar ME and Pastor-de-Ward CT. Fission, regeneration, gonad production and lipids storage in the pyloric caeca of the sea star Allostichaster capensis. Journal of Experimental Marine Biology and Ecology. 2011;409 1:247-52. doi:https://doi.org/10.1016/j.jembe.2011.09.004.
28. Warner JF, Miranda EL and McClay DR. Contribution of hedgehog signaling to the establishment of left-right asymmetry in the sea urchin. Dev Biol. 2016;411 2:314-24. doi:10.1016/j.ydbio.2016.02.008.
29. Mukhopadhyay S, Wen X, Ratti N, Loktev A, Rangell L, Scales SJ, et al. The ciliary G-protein-coupled receptor Gpr161 negatively regulates the Sonic hedgehog pathway via cAMP signaling. Cell. 2013;152 1-2:210-23. doi:10.1016/j.cell.2012.12.026.
30. Reddien PW. Constitutive gene expression and the specification of tissue identity in adult planarian biology. Trends Genet. 2011;27 7:277-85. doi:10.1016/j.tig.2011.04.004.
31. Zhang M, Zhang Y, Scheuring CF, Wu CC, Dong JJ and Zhang HB. Preparation of megabase-sized DNA from a variety of organisms using the nuclei method for advanced genomics research. Nat Protoc. 2012;7 3:467-78. doi:10.1038/nprot.2011.455.
32. Porebski S, Bailey LG and Baum BR. Modification of a CTAB DNA extraction protocol for plants containing high polysaccharide and polyphenol components. Plant Molecular Biology Reporter. 1997;15 1:8-15. doi:10.1007/BF02772108.
33. Bolger AM, Lohse M and Usadel B. Trimmomatic: a flexible trimmer for Illumina sequence data. Bioinformatics. 2014;30 15:2114-20. doi:10.1093/bioinformatics/btu170.
34. Porechop. https://github.com/rrwick/Porechop (2017).
35. De Coster W, D'Hert S, Schultz DT, Cruts M and Van Broeckhoven C. NanoPack: visualizing and processing long-read sequencing data. Bioinformatics. 2018;34 15:2666-9.

doi:10.1093/bioinformatics/bty149.
36. Marcais G and Kingsford C. A fast, lock-free approach for efficient parallel counting of occurrences of k-mers. Bioinformatics. 2011;27 6:764-70. doi:10.1093/bioinformatics/btr011.
37. Ranallo-Benavidez TR, Jaron KS and Schatz MC. GenomeScope 2.0 and Smudgeplot for reference-free profiling of polyploid genomes. Nat Commun. 2020;11 1:1432. doi:10.1038/s41467-020-14998-3.
38. NextOmics: NextDeNovo. https://github.com/Nextomics/NextDenovo (2019).
39. Hu J, Fan J, Sun Z and Liu S. NextPolish: a fast and efficient genome polishing tool for long-read assembly. Bioinformatics. 2020;36 7:2253-5. doi:10.1093/bioinformatics/btz891.
40. Servant N, Varoquaux N, Lajoie BR, Viara E, Chen CJ, Vert JP, et al. HiC-Pro: an optimized and flexible pipeline for Hi-C data processing. Genome Biol. 2015;16:259. doi:10.1186/s13059-015-0831-x.
41. Durand NC, Shamim MS, Machol I, Rao SS, Huntley MH, Lander ES, et al. Juicer Provides a One-Click System for Analyzing Loop-Resolution Hi-C Experiments. Cell Syst. 2016;3 1:95-8. doi:10.1016/j.cels.2016.07.002.
42. Durand NC, Robinson JT, Shamim MS, Machol I, Mesirov JP, Lander ES, et al. Juicebox Provides a Visualization System for Hi-C Contact Maps with Unlimited Zoom. Cell Syst. 2016;3 1:99-101. doi:10.1016/j.cels.2015.07.012.
43. Simao FA, Waterhouse RM, Ioannidis P, Kriventseva EV and Zdobnov EM. BUSCO: assessing genome assembly and annotation completeness with single-copy orthologs. Bioinformatics. 2015;31 19:3210-2. doi:10.1093/bioinformatics/btv351.
44. Rhie A, Walenz BP, Koren S and Phillippy AM. Merqury: reference-free quality, completeness, and phasing assessment for genome assemblies. Genome Biol. 2020;21 1:245. doi:10.1186/s13059-020-02134-9.
45. Bushmanova E, Antipov D, Lapidus A and Prjibelski AD. rnaSPAdes: a de novo transcriptome assembler and its application to RNA-Seq data. Gigascience. 2019;8 9 doi:10.1093/gigascience/giz100.
46. TransDecoder. https://github.com/TransDecoder/TransDecoder (2015).
47. Flynn JM, Hubley R, Goubert C, Rosen J, Clark AG, Feschotte C, et al. RepeatModeler2 for automated genomic discovery of transposable element families. Proc Natl Acad Sci U S A. 2020;117 17:9451-7. doi:10.1073/pnas.1921046117.
48. Xu Z and Wang H. LTR_FINDER: an efficient tool for the prediction of full-length LTR retrotransposons. Nucleic Acids Res. 2007;35 Web Server issue:W265-8. doi:10.1093/nar/gkm286.
49. Smit A, Hubley, R & Green, P: RepeatMasker Open-4.0. http://www.repeatmasker.org (2013-2015).
50. Bao W, Kojima KK and Kohany O. Repbase Update, a database of repetitive elements in eukaryotic genomes. Mob DNA. 2015;6:11. doi:10.1186/s13100-015-0041-9.
51. Stanke M, Diekhans M, Baertsch R and Haussler D. Using native and syntenically mapped

cDNA alignments to improve de novo gene finding. Bioinformatics. 2008;24 5:637-44. doi:10.1093/bioinformatics/btn013.

52. Stanke M, Schoffmann O, Morgenstern B and Waack S. Gene prediction in eukaryotes with a generalized hidden Markov model that uses hints from external sources. BMC Bioinformatics. 2006;7:62. doi:10.1186/1471-2105-7-62.

53. Lomsadze A, Burns PD and Borodovsky M. Integration of mapped RNA-Seq reads into automatic training of eukaryotic gene finding algorithm. Nucleic Acids Res. 2014;42 15:e119. doi:10.1093/nar/gku557.

54. Hoff KJ, Lange S, Lomsadze A, Borodovsky M and Stanke M. BRAKER1: Unsupervised RNA-Seq-Based Genome Annotation with GeneMark-ET and AUGUSTUS. Bioinformatics. 2016;32 5:767-9. doi:10.1093/bioinformatics/btv661.

55. Hoff KJ, Lomsadze A, Borodovsky M and Stanke M. Whole-Genome Annotation with BRAKER. Methods Mol Biol. 2019;1962:65-95. doi:10.1007/978-1-4939-9173-0_5.

56. Li H, Handsaker B, Wysoker A, Fennell T, Ruan J, Homer N, et al. The Sequence Alignment/Map format and SAMtools. Bioinformatics. 2009;25 16:2078-9. doi:10.1093/bioinformatics/btp352.

57. Barnett DW, Garrison EK, Quinlan AR, Stromberg MP and Marth GT. BamTools: a C++ API and toolkit for analyzing and managing BAM files. Bioinformatics. 2011;27 12:1691-2. doi:10.1093/bioinformatics/btr174.

58. Buchfink B, Xie C and Huson DH. Fast and sensitive protein alignment using DIAMOND. Nat Methods. 2015;12 1:59-60. doi:10.1038/nmeth.3176.

59. Leskovec J and Sosic R. SNAP: A General Purpose Network Analysis and Graph Mining Library. ACM Trans Intell Syst Technol. 2016;8 1 doi:10.1145/2898361.

60. Slater GS and Birney E. Automated generation of heuristics for biological sequence comparison. BMC Bioinformatics. 2005;6:31. doi:10.1186/1471-2105-6-31.

61. Haas BJ, Salzberg SL, Zhu W, Pertea M, Allen JE, Orvis J, et al. Automated eukaryotic gene structure annotation using EVidenceModeler and the Program to Assemble Spliced Alignments. Genome Biol. 2008;9 1:R7. doi:10.1186/gb-2008-9-1-r7.

62. Bairoch A and Apweiler R. The SWISS-PROT protein sequence database and its supplement TrEMBL in 2000. Nucleic Acids Res. 2000;28 1:45-8. doi:10.1093/nar/28.1.45.

63. Mistry J, Chuguransky S, Williams L, Qureshi M, Salazar Gustavo A, Sonnhammer ELL, et al. Pfam: The protein families database in 2021. Nucleic Acids Research. 2020;49 D1:D412-D9. doi:10.1093/nar/gkaa913.

64. Camacho C, Coulouris G, Avagyan V, Ma N, Papadopoulos J, Bealer K, et al. BLAST+: architecture and applications. BMC Bioinformatics. 2009;10:421. doi:10.1186/1471-2105-10-421.

65. Jones P, Binns D, Chang HY, Fraser M, Li W, McAnulla C, et al. InterProScan 5: genome-scale protein function classification. Bioinformatics. 2014;30 9:1236-40. doi:10.1093/bioinformatics/btu031.

66. Kudtarkar P and Cameron RA. Echinobase: an expanding resource for echinoderm genomic information. Database (Oxford). 2017;2017 doi:10.1093/database/bax074.
67. Emms DM and Kelly S. OrthoFinder: phylogenetic orthology inference for comparative genomics. Genome Biology. 2019;20 1:238. doi:10.1186/s13059-019-1832-y.
68. Mendes FK, Vanderpool D, Fulton B and Hahn MW. CAFE 5 models variation in evolutionary rates among gene families. Bioinformatics. 2020; doi:10.1093/bioinformatics/btaa1022.
69. Wu T, Hu E, Xu S, Chen M, Guo P, Dai Z, et al. clusterProfiler 4.0: A universal enrichment tool for interpreting omics data. Innovation (N Y). 2021;2 3:100141. doi:10.1016/j.xinn.2021.100141.
70. Penn O, Privman E, Ashkenazy H, Landan G, Graur D and Pupko T. GUIDANCE: a web server for assessing alignment confidence scores. Nucleic Acids Res. 2010;38 Web Server issue:W23-8. doi:10.1093/nar/gkq443.
71. Sela I, Ashkenazy H, Katoh K and Pupko T. GUIDANCE2: accurate detection of unreliable alignment regions accounting for the uncertainty of multiple parameters. Nucleic Acids Res. 2015;43 W1:W7-14. doi:10.1093/nar/gkv318.
72. Landan G and Graur D. Local reliability measures from sets of co-optimal multiple sequence alignments. Pac Symp Biocomput. 2008:15-24.
73. Suyama M, Torrents D and Bork P. PAL2NAL: robust conversion of protein sequence alignments into the corresponding codon alignments. Nucleic Acids Res. 2006;34 Web Server issue:W609-12. doi:10.1093/nar/gkl315.
74. Yang Z. PAML 4: phylogenetic analysis by maximum likelihood. Mol Biol Evol. 2007;24 8:1586-91. doi:10.1093/molbev/msm088.

Figures

Figure 1: A. Adult *Plazaster borealis*. Photograph by National Institute of Biological Resources (NIBR, https://www.nibr.go.kr) B. Sampling spot of *P. borealis* studied in this research.

Figure 2: A phylogenetic tree of *P. borealis* and six other species. This tree was constructed using protein sequences of seven species, showing gene family expansion and contraction. The number below the branches represents the number of gene families with either expansion (blue) and contraction (red). The ratio of expanded and contracted gene families was expressed in the pie chart above the branches. The numbers at the node indicate the bootstrap value. The species

used in the tree are *P. borealis*, *Asterias rubens*, *Acanthaster planci*, *Patiria miniata*, *Lytechinus variegatus*, *Parastichopus parvimensis*, and *Strongylocentrotus purpuratus*.

Figure 3: Syntenic relationship of *P. borealis* and species of the order Forcipulatida. A. Synteny between *Asterias rubens* and *P. borealis*. The syntenic blocks were calculated with MCscan. B-D. Syntenic relationship of *P. borealis* between *A. rubens* (B), *Pisaster ochraceus* (C), *Marthasterias glacialis* (D) Genomic sequences were compared with Chromeister based on inexact k-mer matching.

Figure 4: GO enrichment analysis of expanded gene families of *P. borealis.*

Figure 5: Results of GO enrichment analysis of positively selected genes. BP: GO Term Biological Process (green), CC: GO Term Cellular Component (red), KEGG: Kyoto Encyclopedia of Genes and Genomes (blue).

Tables

Table 1: *Plazaster borealis* assembly statistics

Assembly statistics	Value	
Genome size (bp)		561,050,340
Number of scaffolds		801
Number of chromosome-scale scaffolds		22
N50 of scaffolds (bp)		24,975,817
L50 of scaffolds		10
Chromosome-scale scaffolds (bp)		518,884,334
GC content of the genome (%)		38.89
QV score		36.3457
Error rate		0.00023
BUSCO analysis		
Library	Eukaryota	Metazoa
Complete	252 (98.8%)	935 (98.0%)
Complete and single-copy	249 (97.6%)	925 (97.0%)
Complete and duplicated	3 (1.2%)	10 (1.0%)
Fragmented	2 (0.8%)	11 (1.2%)
Missing	1 (0.4%)	8 (0.8%)

Table 2: *Plazaster borealis* repetitive DNA elements

Type	Number of elements	Length occupied (bp)	Percentage of sequence (%)
DNA	10,734	3,597,965	0.64
LINE	42,851	3,472,043	0.62
SINE	60,394	13,931,402	2.48
LTR	8,277	5,145,127	0.92
Satellite	9	2,752	0
Small RNA	20,889	1,464,546	0.26
Simple repeat	162,149	8,016,020	1.43
Unclassified	1,294,477	249,314,223	44.44
Low complexity	25,170	1,365,485	0.24
Total			51.05%

Table 3: *Plazaster borealis* genome annotation statistics

Statistic	Value
Number of predicted genes	26,836
Number of predicted protein-coding genes	25,224
Average gene length	8,948.89
Number of transcripts	26,737
Average transcript length (bp)	1,502.90
Number of exons	192,343
Average exon length (bp)	213.57
Average exon per transcript	7.19
Number of introns	165,606
Average intron length (bp)	1,261.88
Number of genes annotated to Swiss-Prot	18,451
Number of genes annotated to PFAM	18,541
Number of genes annotated to NR	24,229
BUSCO analysis	
Complete (%)	884 (92.6%)
Complete and single-copy (%)	859 (90.0%)
Complete and duplicated (%)	25 (2.6%)
Fragmented (%)	44 (4.6%)
Missing (%)	26 (2.8%)

Table 4: Genes with accelerated evolution in the *P. borealis*.

Gene	H0_lnl	H1_lnl	Likelihood ratio	FDR	# of positively selected sites*
GPR161	-8827.28	-8798.95	56.66761	2.06E-13	5
RPL5	-3991.54	-3968.12	46.84587	2.3E-11	1
RSL24D1	-2215.1	-2192.93	44.35075	6.59E-11	14
PHB2	-4815.8	-4805.98	19.631658	1.61E-05	4
NAA10	-4703.42	-4694.3	18.237898	2.92E-05	4
IQCA1	-9112.13	-9103.79	16.684644	5.88E-05	2
SLC30A5	-10574.5	-10566.6	15.766218	8.6E-05	3
BMP10	-8017.18	-8010.17	14.034764	0.000196	4
STOML2	-5414.16	-5408.06	12.206464	0.000476	1
ACYP1	-1855.62	-1849.54	12.153438	0.000452	3
NIPSNAP3A	-4951.12	-4946.47	9.296206	0.001968	1

H0_lnl: log likelihood given H0 (ω does not vary across the branches), H1_lnl: log likelihood

given H1, *Number of positively selected sites with a BEB of > 0.95.

Figure1

Click here to access/download;Figure;Figure1_Adult_Plazaster_borealis_and_sam

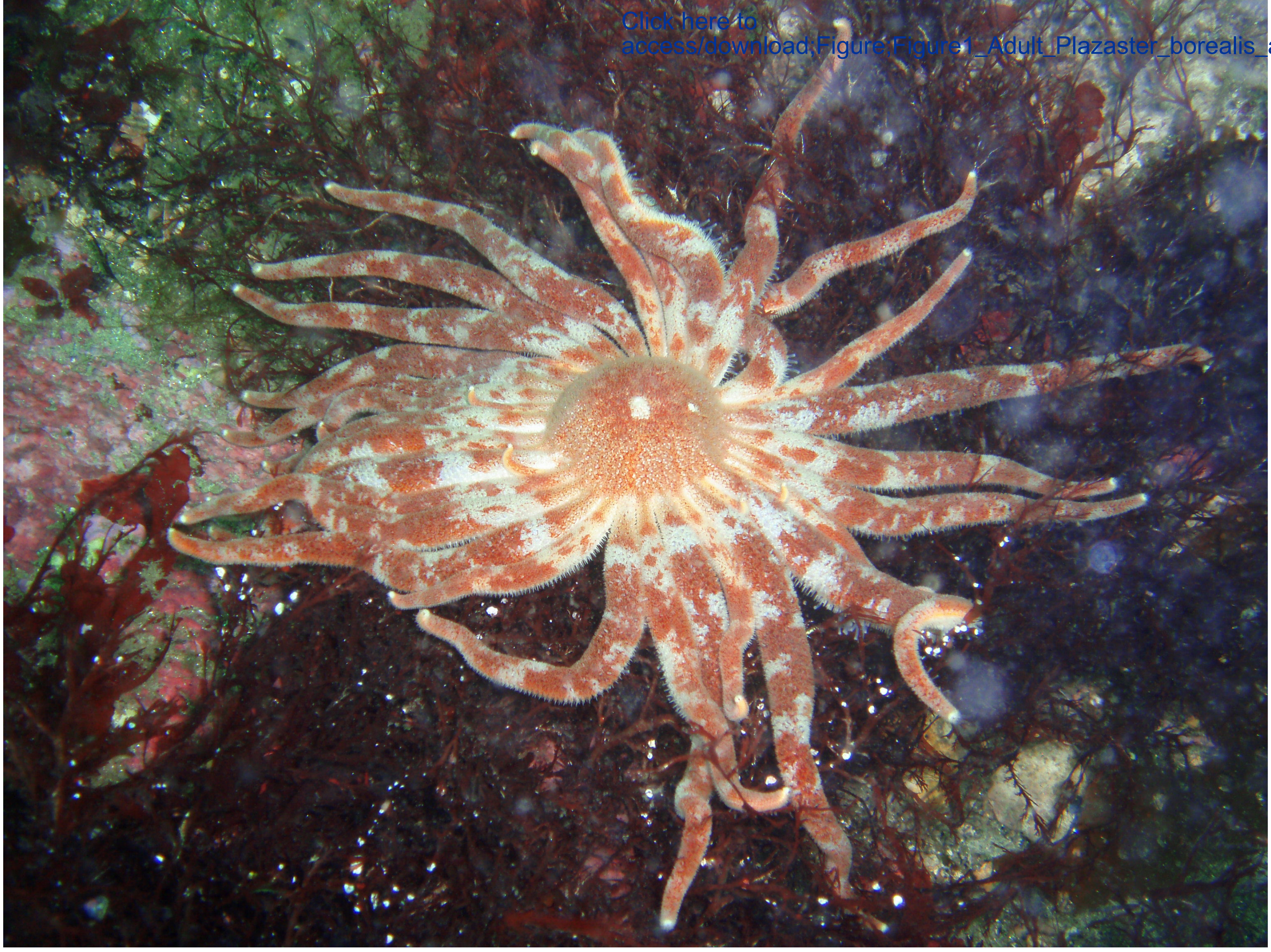

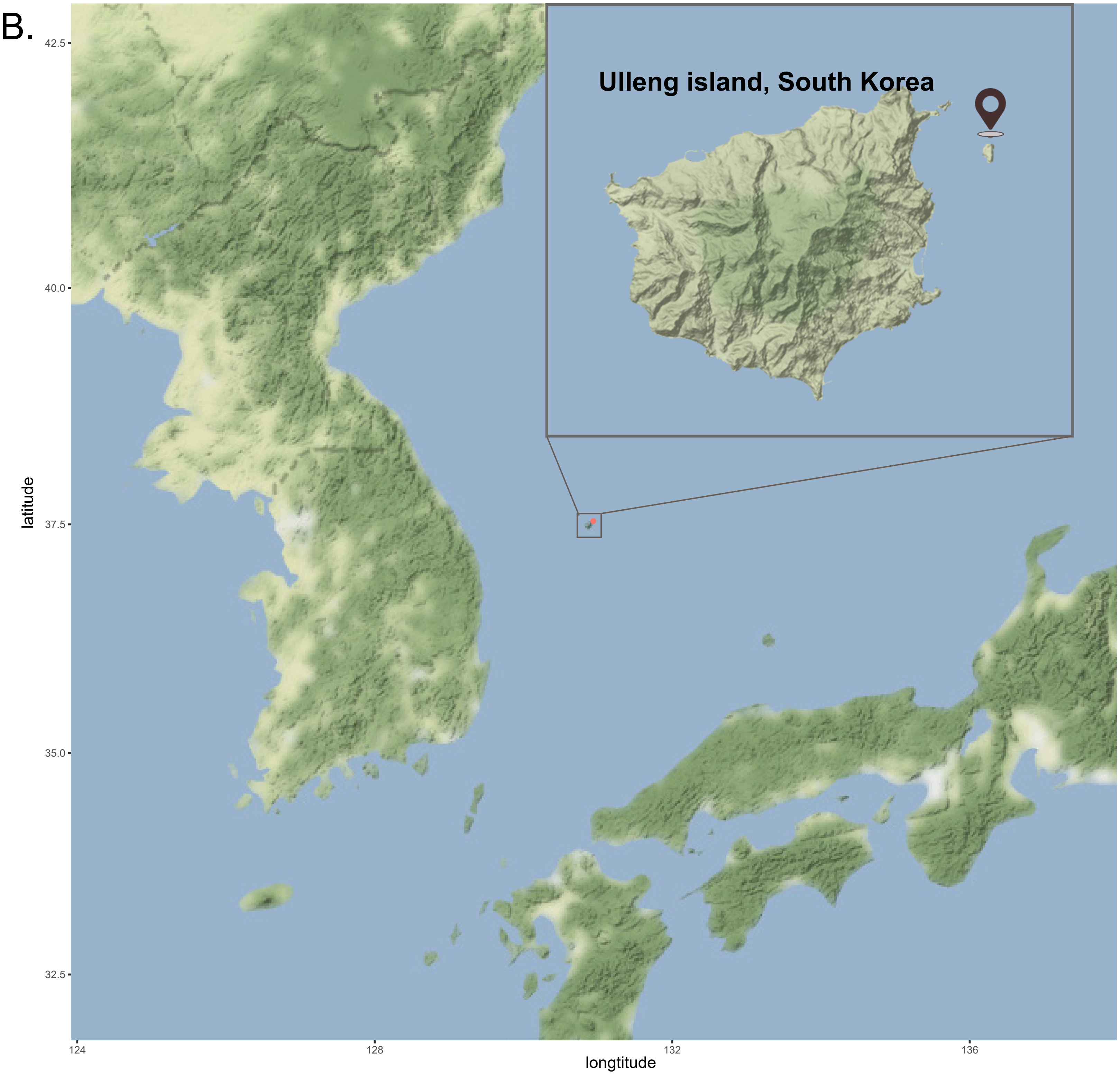

Figure2

Click here to access/download;Figure;Figure2_Phylogeny_tree.pdf

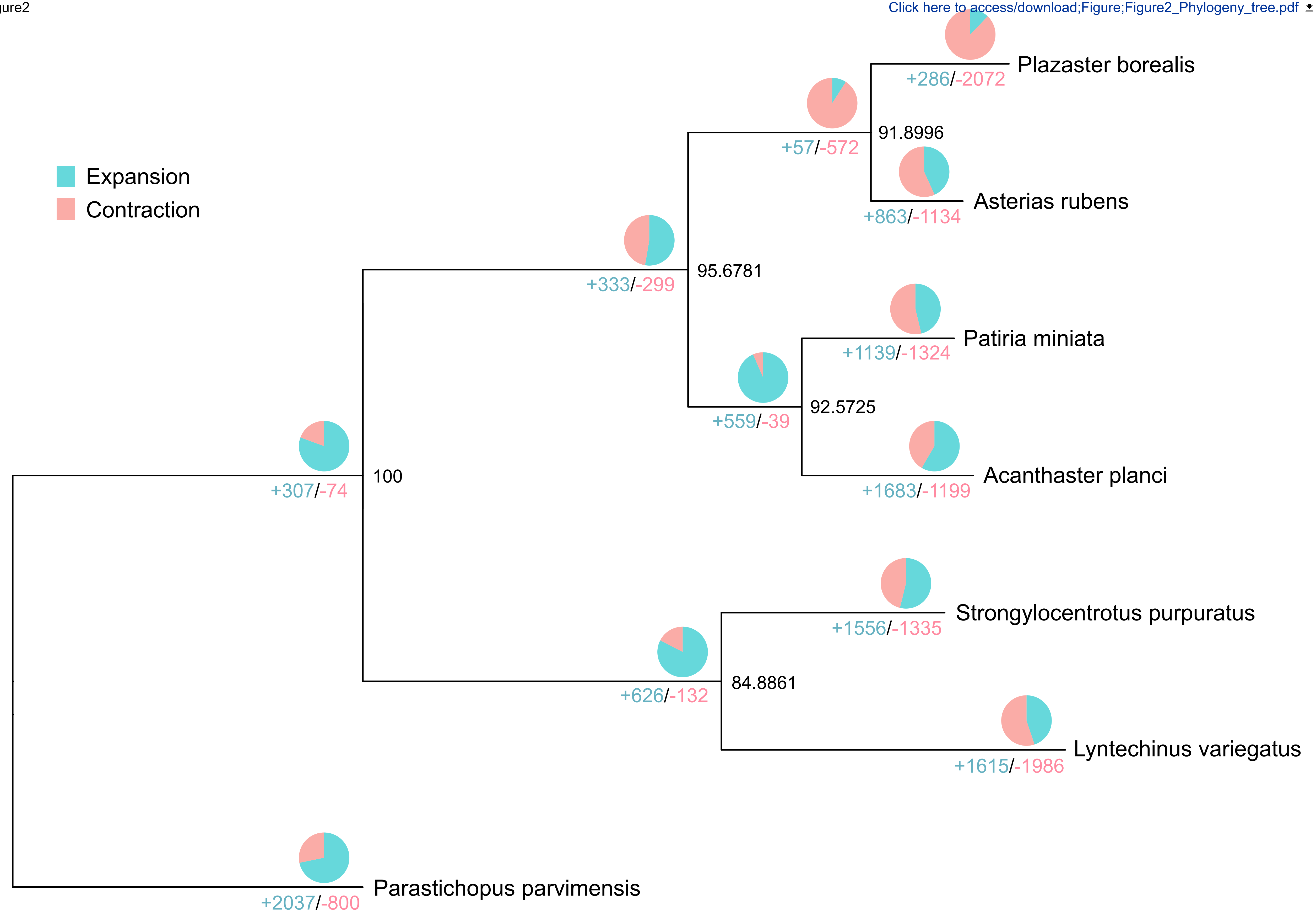

Figure3

Click here to access/download;Figure;Figure3_Syntenic_relationship.p

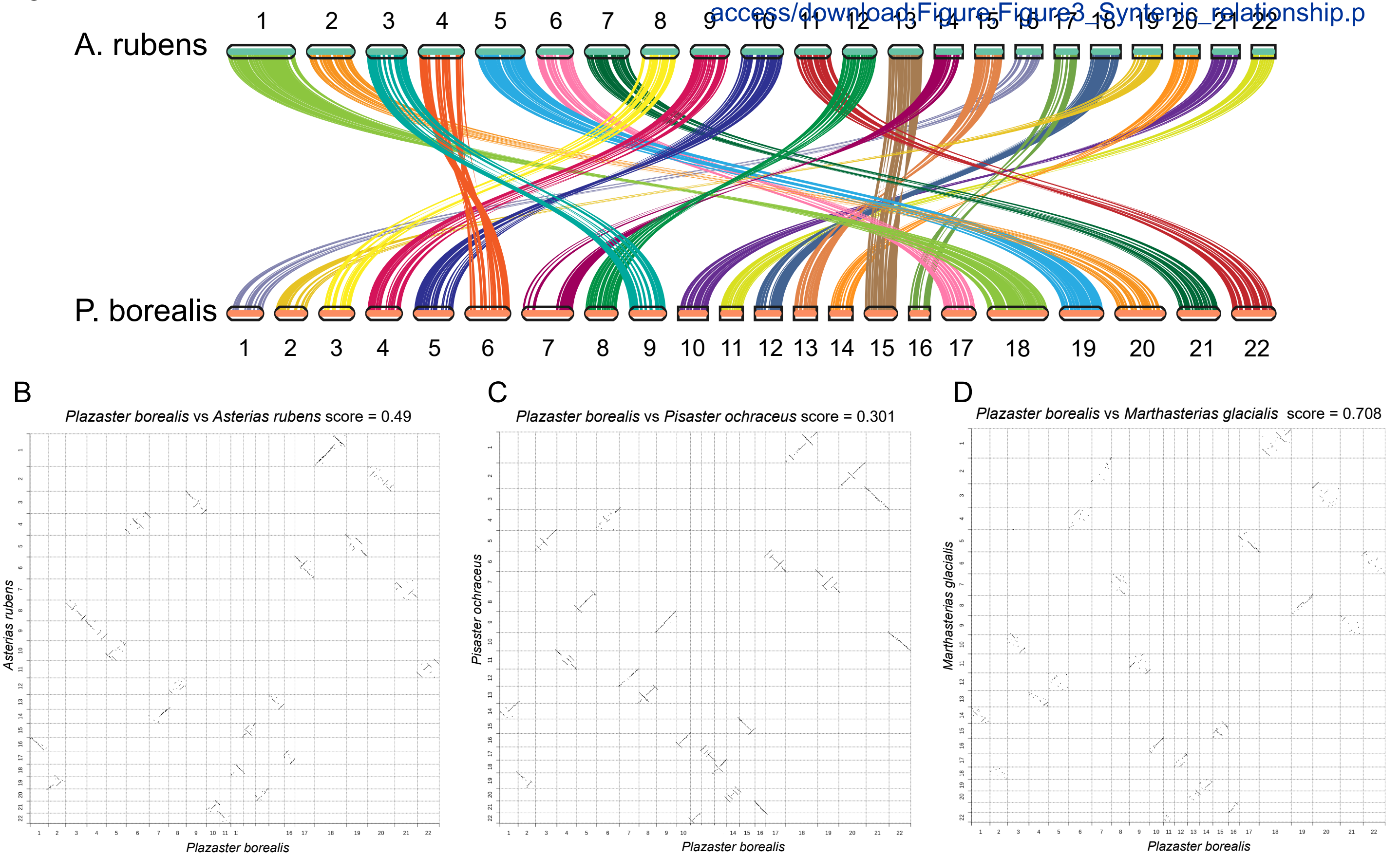

Figure4

Click here to access/download;Figure;Figure4_GO_enrichment_of_exp_gene_Pborealis.pdf

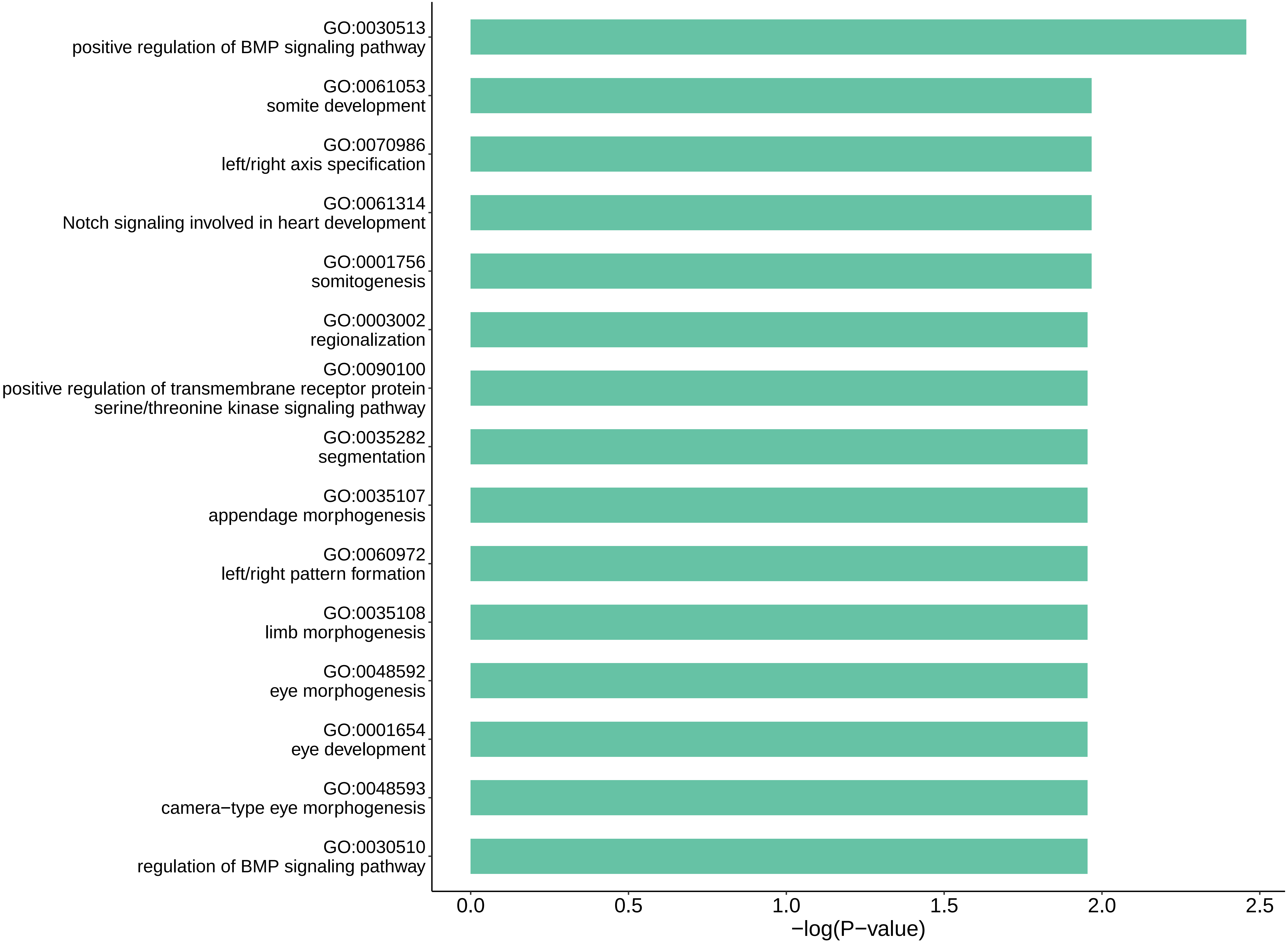

Figure5

Click here to access/download;Figure;Figure5_GO_enrichment_of_positively_selected_genes.pdf

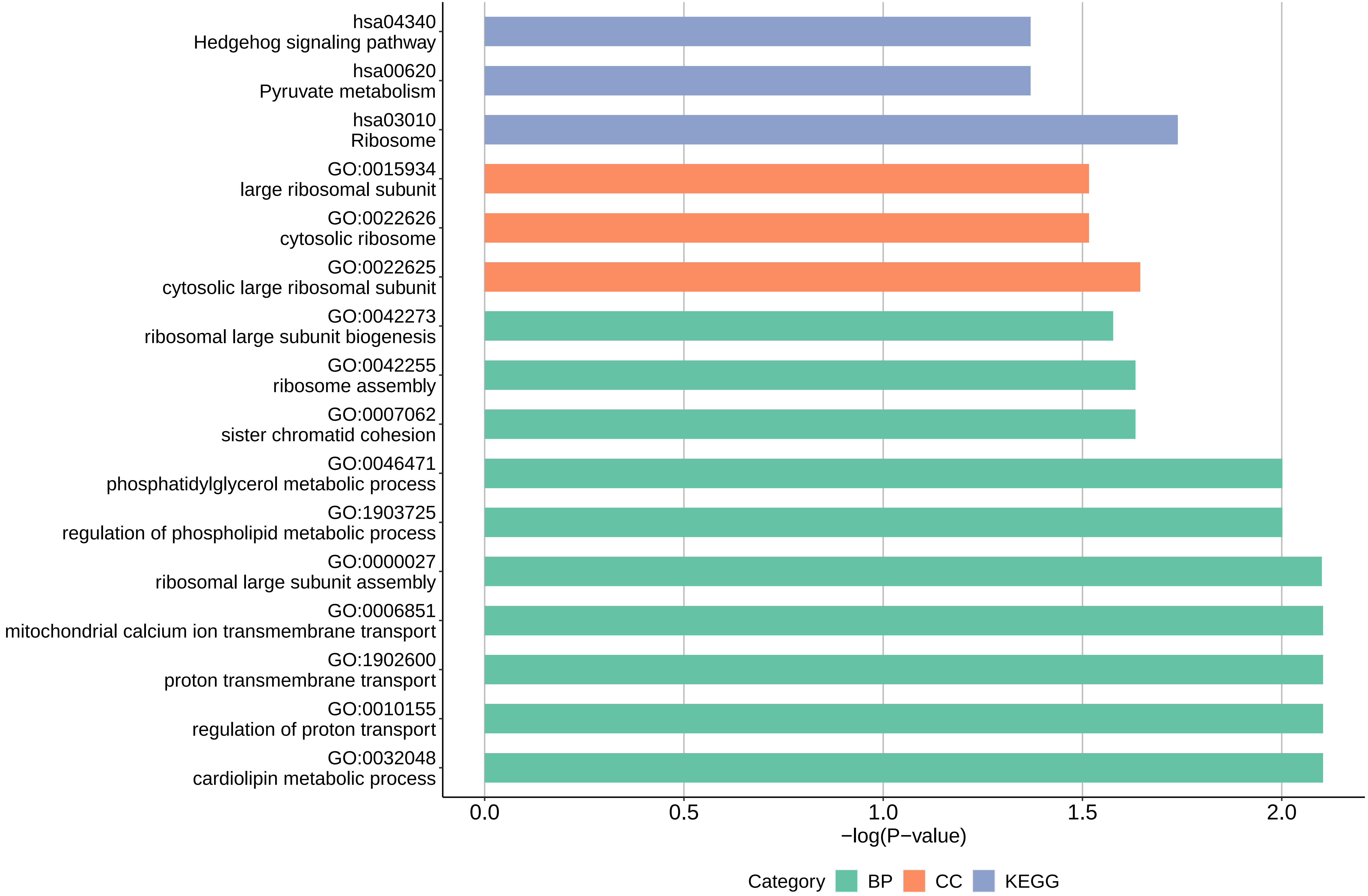

Supplementary Figure1

Click here to access/download
Supplementary Material
Supp_Fig1_Genome_size_estimation.png

Supplementary Figure2

Click here to access/download
Supplementary Material
Supp_Fig2_Plazaster_borealis_genome_assembly_completeness.pdf

Supplementary Table1

Click here to access/download
Supplementary Material
Supp_Table1_Statistics_of_raw_sequencing_data.xlsx

Supplementary Table2

Click here to access/download
Supplementary Material
Supp_Table2_Statistics_of_assembly_before_scaffolding.xlsx

Supplementary Table3

Click here to access/download

Supplementary Material

Supp_Table3_GO_KEGG_enrichment_of_con&exp_of_7_echinoderms.xlsx

Supplementary Table4

Click here to access/download
Supplementary Material
Supp_Table4_GO_KEGG_enrichment_of_Pborealis_specific_ortho.xlsx

Supplementary Table5

Click here to access/download
Supplementary Material
Supp_Table5_GO_KEGG_enrichment_of_positively_selected_genes.xlsx
