# Supplementary material for: Chromosome-level genome assembly of Plazaster borealis sheds light on the morphogenesis of multiarmed starfish and its regenerative capacity
Source: Gigascience. 2022 Jul 9;11:giac063. doi: 10.1093/gigascience/giac063 (PMC9270726; doi:10.1093/gigascience/giac063)
Supplement: giac063_GIGA-D-21-00378_Revision_2 [file giac063_giga-d-21-00378_revision_2.pdf]

GigaScience

Chromosome-level genome assembly of Plazaster borealis: shed light on the morphogenesis of multi-armed starfish and its regenerative capacity

--Manuscript Draft--

Manuscript Number:	GIGA-D-21-00378R2
Full Title:	Chromosome-level genome assembly of Plazaster borealis: shed light on the morphogenesis of multi-armed starfish and its regenerative capacity
Article Type:	Data Note
Funding Information:	National Institute of Biological Resources (NIBR201930201) — PhD Jaewoong Yu
Abstract:	Background: Plazaster borealis has a unique morphology displaying multiple arms with a clear distinction between disk and arms, rather than displaying pentaradial symmetry, a remarkable characteristic of Echinoderms. Herein we report the first chromosome-level reference genome of P. borealis and an essential tool to further investigate the basis of the divergent morphology.<br>Findings: Total 57.76 Gb of a long read and 70.83 Gb of short-read data were generated to assemble de novo 561Mb reference genome of P. borealis, and Hi-C sequencing data (57.47 Gb) was used for scaffolding into 22 chromosomal scaffolds comprising 92.38% of the genome. The genome completeness estimated by BUSCO is of 98.0% using the metazoan set, indicating a high-quality assembly. Through the comparative genome analysis, we identified evolutionary accelerated genes known to be involved in morphogenesis and regeneration, suggesting their potential role in shaping body pattern and capacity of regeneration.<br>Conclusion: This first chromosome-level genome assembly of P. borealis provides fundamental insights into echinoderm biology, as well as the genomic mechanism underlying its unique morphology and regeneration.
Corresponding Author:	Jaewoong Yu<br>eGnome Inc<br>Seoul, KOREA, REPUBLIC OF
Corresponding Author Secondary Information:	
Corresponding Author's Institution:	eGnome Inc
Corresponding Author's Secondary Institution:	
First Author:	Yujung Lee
First Author Secondary Information:	
Order of Authors:	Yujung Lee
	Bongsang Kim
	Jaehoon Jung
	Bomin Koh
	So Yun Jhang
	Chaeyoung Ban
	Won-Jae Chi
	Soonok Kim
	Jaewoong Yu
Order of Authors Secondary Information:	
Response to Reviewers:	Reviewer #2:<br>1.Suggestions and editions of the language.

Powered by Editorial Manager® and ProduXion Manager® from Aries Systems Corporation

	We revised all the suggested sentences in the manuscript.
**Additional Information:**	
**Question**	**Response**
Are you submitting this manuscript to a special series or article collection?	No
**Experimental design and statistics**<br><br>Full details of the experimental design and statistical methods used should be given in the Methods section, as detailed in our Minimum Standards Reporting Checklist. Information essential to interpreting the data presented should be made available in the figure legends.<br><br>Have you included all the information requested in your manuscript?	Yes
**Resources**<br><br>A description of all resources used, including antibodies, cell lines, animals and software tools, with enough information to allow them to be uniquely identified, should be included in the Methods section. Authors are strongly encouraged to cite Research Resource Identifiers (RRIDs) for antibodies, model organisms and tools, where possible.<br><br>Have you included the information requested as detailed in our Minimum Standards Reporting Checklist?	Yes
**Availability of data and materials**<br><br>All datasets and code on which the conclusions of the paper rely must be either included in your submission or deposited in publicly available repositories (where available and ethically appropriate), referencing such data using a unique identifier in the references and in the "Availability of Data and Materials"	Yes

section of your manuscript.<br><br>Have you have met the above requirement as detailed in our Minimum Standards Reporting Checklist?	

Powered by Editorial Manager® and ProduXion Manager® from Aries Systems Corporation

Manuscript

Click here to access/download;Manuscript;378-EditorR1_revised.docx

Chromosome-level genome assembly of *Plazaster borealis* shed light on the morphogenesis of multi-armed starfish and its regenerative capacity

Yujung Lee[1] [0000-0003-2279-3147]; Bongsang Kim[1,2] [0000-0001-7526-8421]; Jaehoon Jung[1,2] [0000-0003-2019-0895]; Bomin Koh[1] [0000-0001-6702-6449]; So Yun Jhang[1,3] [0000-0002-2152-3746]; Chaeyoung Ban[1] [0000-0003-4566-4313]; Won-Jae Chi[4] [0000-0003-2893-7930]; Soonok Kim[4] [0000-0003-1654-3643]; Jaewoong Yu[1,*] [0000-0002-4120-8890];

[1]eGnome, Inc., 26 Beobwon-ro 9-gil, Sonpa-gu, Seoul 05836, Republic of Korea;

[2]Department of Agricultural and Life Sciences and Research Institute of Population Genomics, Seoul National University, Seoul, Republic of Korea;

[3]Interdisciplinary Program in Bioinformatics, Seoul National University, Seoul, 151-742, Republic of Korea;

[4]Microorganism Resources Division, National Institute of Biological Resources, Incheon 22689, Republic of Korea;

*Correspondence address: Jaewoong Yu, eGnome Inc., 26 Beobwon-ro 9-gil, Sonpa-gu, Seoul 05836, Korea. Email: jwyu@egnome.co.kr; Tel.: +82-070-4694-6355

Email addresses/ ORCIDs

Yujung Lee[1]: lyjung711@gmail.com, lyjung7@egnome.co.kr/ 0000-0003-2279-3147

Bongsang Kim[1,2]: babybird93@snu.ac.kr, kimbongsang@egnome.co.kr / 0000-0001-7526-8421

Jaehoon Jung[1,2]: motto@snu.ac.kr, motto@egnome.co.kr / 0000-0003-2019-0895

Bomin Koh[1]: chloekoh@egnome.co.kr / 0000-0001-6702-6449

So Yun Jhang[1,3]: soyun4595@snu.ac.kr, soyun4595@egnome.co.kr / 0000-0002-2152-3746

Chaeyoung Ban[1]: terryban@egnome.co.kr / 0000-0003-4566-4313

Won-Jae Chi[34]: wjchi76@korea.kr / 0000-0003-2893-7930

Soonok Kim[4]: sokim90@korea.kr / 0000-0003-1654-3643

Jaewoong Yu[1,*]: jwyu@egnome.co.kr / 0000-0002-4120-8890

Abstract

Background: *Plazaster borealis* has a unique morphology displaying multiple arms with a clear distinction between disk and arms, rather than displaying pentaradial symmetry, a remarkable characteristic of Echinoderms. Herein we report the first chromosome-level reference genome of *P. borealis* and an essential tool to further investigate the basis of the divergent morphology.

Findings: Total 57.76 Gb of a long read and 70.83 Gb of short-read data were generated to assemble *de novo* 561Mb reference genome of *P. borealis,* and Hi-C sequencing data (57.47 Gb) was used for scaffolding into 22 chromosomal scaffolds comprising 92.38% of the genome. The genome completeness estimated by BUSCO is of 98.0% using the metazoan set, indicating a high-quality assembly. Through the comparative genome analysis, we identified evolutionary accelerated genes known to be involved in morphogenesis and regeneration, suggesting their potential role in shaping body pattern and capacity of regeneration.

Conclusion: This first chromosome-level genome assembly of *P. borealis* provides fundamental insights into echinoderm biology, as well as the genomic mechanism underlying its unique morphology and regeneration.

Data Description

Context

Echinoderms are marine animals characterized by the following three remarkable characteristics: 1) extensive regenerative abilities in both adult and larval forms [1, 2], 2) the water vascular system used for gas, nutrient and waste exchange [3], and 3) extraordinary morphological characteristics including pentaradial symmetry [4, 5].

Pentaradial symmetry has been observed in all extant classes of echinoderm. Echinoids (sea urchin) and holothurians (sea cucumber) always have five ambulacral grooves, and crinoids have many arms in multiples of five that branch out from the five primary brachia [4, 5]. Most species of asteroids and ophiuroids are five-armed, but many exceptions are scattered across the tree of Echinodermata. Extant asteroids are distinguished by 34 families, including 20 families of only five-armed species, nine families of both five-armed and multi-armed species, and five families with exclusively multi-armed species [6]. However, most multi-armed forms have arm numbers that cannot be divided into five, raising questions about the arm development mechanisms that do not follow the pentaradial symmetry.

The octopus starfish, *Plazaster borealis* (NCBI:txid466999; marinespecies.org:taxname:254846), is a starfish that inhabits the water that surround Korea and Japan [7, 8]. It belongs to the family *Labidiasteridae*, one of five exclusively multi-armed families [6]. Figure 1A illustrates a unique morphology of *P. borealis* that the number of arms is around 31~40, which is a large number among multi-armed starfishes, and it shows a clear differentiation between arms and central disks [9].

In the previous study of *P. borealis*, Matsuoka et al. investigated the molecular phylogenetic relationship of five species from the order Forcipulatida: *Asterias amurensis*, *Aphelasterias japonica*, *Distolasterias nipon*, *Coscinasterias acutispina*, and *Plazaster borealis* [10]. *P. borealis* was the most closely related with five armed *A. amurensis* and distantly related with multi-armed *C. acutispina*. The result suggested that the unique morphology of *P. borealis* might have descended from a five-armed starfish, which possibly resulted from accelerated sequence evolution. However, the absence of a reference genome has limited in-depth research. To understand the genetic basis of the specialized morphology of the starfish, we sequenced the genome of *P. borealis* and performed comparative genomic analyses with the high-quality

of well-annotated genome sequences of six other echinoderms (*Asterias rubens*, *Acanthaster planci*, *Patiria miniata*, *Lytechinus variegatus*, *Parastichopus parvimensis*, and *Strongylocentrotus purpuratus*).

Chromosome-level genome assembly of the octopus starfish

We estimated the genome size of *P. borealis* with GenomeScope[40] to be ~497Mb (Supplementary Figure 1). A comprehensive sequencing data set was generated for the *P. borealis* genome assembly based on this estimation. From the Nanopore sequencing platform, a total of 57.76 Gb long read was yielded with 116x coverage. Using the Illumina sequencing platform, 142x coverage of Illumina short paired-end read sequencing data and 115x coverage of Hi-C paired-end reads were generated (Supplementary Table 1). Moreover, we sequenced 25.63 Gb of RNA Illumina short paired-end reads and 7.28 Gb of RNA Nanopore long reads to construct transcriptome assembly utilized for annotation.

A draft genome assembly was generated, consisting of 179 contigs totaling 561Mb with an N50 of 11Mb (Supplementary Table 2). We then scaffolded the contigs using Hi-C data with 3D-DNA to obtain chromosomal information [11]. The total size of the final assembly was 561Mb comprising 22 chromosome-level scaffolds with a contig N50 of 24Mb. These 22 chromosome-level scaffolds comprise 92.48% of the assembly, although the remaining 42 Mb were unanchored and required further investigation (Table 1, Supplementary Figure 2). This number is consistent with chromosome results of other species of the order Forcipulatida, supporting the accurate chromosome number acquired in the current study.

Table 1: *Plazaster borealis* assembly statistics

Assembly statistics	Value
Genome size (bp)	561,050,340
Number of scaffolds	801
Number of chromosome-scale scaffolds	22
N50 of scaffolds (bp)	24,975,817
L50 of scaffolds	10
Chromosome-scale scaffolds (bp)	518,884,334
GC content of the genome (%)	38.89
QV score	36.3457
Error rate	0.00023
BUSCO analysis	
Library	Metazoan_odb10
Complete	935 (98.0%)
Complete and single-copy	925 (97.0%)
Complete and duplicated	10 (1.0%)
Fragmented	11 (1.2%)
Missing	8 (0.8%)

Completeness of the assembled genome

The genome completeness was evaluated using BUSCO [12] with the metazoan dataset called 'metazoan_odb10'. As a result, total of 935 (98.0%) core metazoan genes were successfully detected in the genome, consisting of 97.0% single-copy, 1.0% duplicated, 1.2% fragmental, and 0.8% missing genes from the metazoan dataset. We also estimated the overall assembly quality by comparing the k-mer distribution of the assemblies and the Illumina short-read sets using Merqury [13]. The genome assembly of *P. borealis* showed high-quality values (QV > 36) with an error rate of 0.00023 (Table 1). Additionally, the GC content of *P. borealis* was 38.89%, which was very similar to that of *A. rubens* (38.76%) and *P. ochraceus* (39.01%), the species of the order Forcipulatida. The assessment results validated the high quality of our final genome assembly. To our knowledge, this is the first high-quality chromosome level genome assembly for *P. borealis* and the first reference genome of the family *Labidiasteridae*.

Annotation of repeats and genes

Repetitive elements accounted for 51.05% of the whole genome assembly, and detailed percentages of the predominant repetitive element families are summarized in Table 2. We annotated a total of 26,836 genes onto the assembled regions. Compared with other starfish, *P. borealis* has a similar average exon length (213 bp) and exon number per gene (7.19), but it has a shorter intron length (1,261 bp) than *A. rubens* (eAstRub1.3). BUSCO benchmarking value of this gene set was summarized as 92.6% of complete genes, including 90% single-copy, 2.6% duplicated, 4.6% fragmental, and 2.8% missing genes from the metazoan dataset. Following a standard functional annotation, we observed that 24,248 (96.13%) genes were successfully annotated with at least one related functional assignment (Table 3).

Table 2: *Plazaster borealis* repetitive DNA elements

Type	Number of elements	Length occupied (bp)	Percentage of sequence (%)
DNA	10,734	3,597,965	0.64
LINE	42,851	3,472,043	0.62
SINE	60,394	13,931,402	2.48
LTR	8,277	5,145,127	0.92
Satellite	9	2,752	0
Small RNA	20,889	1,464,546	0.26
Simple repeat	162,149	8,016,020	1.43
Unclassified	1,294,477	249,314,223	44.44
Low complexity	25,170	1,365,485	0.24
Total			51.05%

Table 3: *Plazaster borealis* genome annotation statistics

Statistic	Value
Number of predicted genes	26,836
Number of predicted protein-coding genes	25,224
Average gene length	8,948.89
Number of transcripts	26,737
Average transcript length (bp)	1,502.90
Number of exons	192,343
Average exon length (bp)	213.57
Average exon per transcript	7.19
Number of introns	165,606
Average intron length (bp)	1,261.88
Number of genes annotated to Swiss-Prot	18,451
Number of genes annotated to PFAM	18,541
Number of genes annotated to NR	24,229
BUSCO analysis	
Complete (%)	884 (92.6%)
Complete and single-copy (%)	859 (90.0%)
Complete and duplicated (%)	25 (2.6%)
Fragmented (%)	44 (4.6%)
Missing (%)	26 (2.8%)

Phylogenetic and syntenic relationship

To understand the phylogenetic placement of *P. borealis*, species tree was inferred from sets of multi-copy gene trees with STAG algorithm [75] based on protein sequences from seven echinoderm genomes: *Asterias rubens*, *Acanthaster planci*, *Patiria miniata*, *Lytechinus variegatus*, *Parastichopus parvimensis*, and *Strongylocentrotus purpuratus*. *P. borealis* was the most closely related to *A. rubens* (Figure 2), consistent with both previous results [10].

Syntenic relationships as inferred by MCscan [14] results were congruent with the phylogenetic results from the STAG analyses. In the genome of *P. borealis* and *A. rubens*, every chromosome matched each other well enough to suggest that the entire chromosomes seem to be highly

conserved, except an additional genomic region detected in chromosome 7 of *P. borealis* (Figure 3A, 3B). A similar tendency, using Chromeister [15], was observed with other species of the order Forcipulatida, *P. ochraceus* and *M. glacialis*. *P. borealis* exhibited more conservation of synteny with *P. ochraceus* than A. rubens, which seems to be influenced by the observed genomic region. We also analyzed synteny of *P. borealis* with *A. planci*, the starfish of a different order; however, chromosomes were not matched. These results suggest that genomes within the Forcipulatida order are remarkably conserved in terms of synteny, allowing us to confirm the high quality of our genome assembly.

Gene family evolution in *P. borealis*

Based on the assumption that the unique morphology of *P. borealis* is explained by accelerated evolutionary rate [10], we performed comparative genomic analyses among seven echinoderm species. Although the genetic mechanism underlying the development of supernumerary arms of starfish is elusive, we hypothesized that genes associated with tissue morphogenesis are increased to produce excessive arms. We tested this hypothesis by performing expansion and contraction analyses of gene families using CAFE5 [16]. Compared with six echinoderm species, 286 gene families were expanded, whereas 2,072 gene families were contracted in *P. borealis* (Figure 2). The significantly expanded genes in the genome of *P. borealis* were significantly enriched in categories of Notch and BMP signaling pathway, body pattern specification, morphogenesis, and eye development (P-value<0.02) (Figure 4). Collectively, these expanded gene families are likely to play an enhanced role in forming supernumerary arms of *P. borealis*. Notch and BMP signaling are evolutionally conserved and play multiple roles during animal development, especially in regulating body patterns. The Notch signaling pathway is essential for cell proliferation, cell fate decisions, and induction of differentiation

Table 4: Genes with accelerated evolution in the *P. borealis*.

Gene	H0_lnl	H1_lnl	Likelihood ratio	FDR	# of positively selected sites*
GPR161	-8827.28	-8798.95	56.66761	2.06E-13	5
RPL5	-3991.54	-3968.12	46.84587	2.3E-11	1
RSL24D1	-2215.1	-2192.93	44.35075	6.59E-11	14
PHB2	-4815.8	-4805.98	19.631658	1.61E-05	4
NAA10	-4703.42	-4694.3	18.237898	2.92E-05	4
IQCA1	-9112.13	-9103.79	16.684644	5.88E-05	2
SLC30A5	-10574.5	-10566.6	15.766218	8.6E-05	3
BMP10	-8017.18	-8010.17	14.034764	0.000196	4
STOML2	-5414.16	-5408.06	12.206464	0.000476	1
ACYP1	-1855.62	-1849.54	12.153438	0.000452	3
NIPSNAP3A	-4951.12	-4946.47	9.296206	0.001968	1

H0_lnl: log likelihood given H0 (ω does not vary across the branches), H1_lnl: log likelihood given H1, *Number of positively selected sites with a BEB of > 0.95.

during embryonic and postnatal development [17-19]. Besides regulating cell-fate decisions at an individual cell level, a cell-to-cell signaling mechanism of Notch coordinates the spatiotemporal patterning in a tissue [20]. In *Drosophila melanogaster*, Notch functions as it is required to specify the fate of the cells that will eventually segment leg and develop leg joint [21, 22]. The mechanisms of BMP gradient formation have been studied in various animals. BMP2/4 signaling study of sea urchin showed that interaction between BMP2/4 and chordin formed the dorsal-ventral gradient and resulted in dorsal-ventral axis patterning [23]. Furthermore, as the physical characteristic of starfish, their eyes exist at the end of each arm denoting that the arm development is accompanied with the eye development. However, contracted gene families of *P. borealis* had no significantly enriched functions, except GTPase regulator activity (GO:0030695, P-value=0.005647). Gene repertories of *P. borealis* showed

differences in the contents of other species' expanded and contracted genes mainly enriched in terms related to the nerve development (Supplementary Table 3).

In addition, we identified 607 gene families unique in *P. borealis* consisting of 2,631 genes and 111 one-to-one orthologous genes between *P. borealis* and six other species. The gene families unique in *P. borealis* are enriched for the following gene ontology (GO) terms: apoptotic cell clearance, positive regulation of epithelial cell proliferation, vascular transport, and activation of JNKK activity (Supplementary Table 4). The enriched term, activation of JNKK activity, is involved in the JNK pathway, which promotes apoptosis by upregulating pro-apoptotic gene expression [24]. Typically, cell proliferation and death are important to achieve tissue formation, involving changes in cell number, size, shape, and position [25]. Based on these findings, the presence of additional genes of the Notch pathway, BMP pathway, and JNK pathway involved in body pattern specification, cell proliferation, and apoptosis could indicate enhanced tissue shaping to form many arms.

The signaling pathways that underwent gene family expansion in the *P. borealis* lineage, especially the Notch and BMP pathways, also play several key conserved roles in the regeneration of many species. For example, in the study of brittle stars, the inhibition of Notch signaling hindered arm regeneration and downregulated genes related to ECM component, cell proliferation, apoptosis, and innate immunity, which are biological processes associated with regeneration [26]. In addition, previous studies of echinoderm gene expression and other animals showed that Notch and BMP signaling are the principal pathways for tissue regeneration [27, 28].

The studies of the metamorphosis of multi-armed starfishes led to the proposal of the 'Five-Plus' hypothesis [6, 29]. It states that five primary arms generated concurrently develop in a controlled unit and supernumerary arms are produced in the separate and independent pathways.

Although these pathways are still uncertain, Hotchkiss suggested two possibilities: post-generation of arms in the incompletely developed starfish or intercalated regeneration of arms in adults [6]. The capacity of regeneration is a remarkable feature of all extant classes of echinoderms [2]. Thus, it is possible that multi-armed starfishes could transform from five-rayed forms to multi-rayed forms by growing new arms through regeneration-related mechanisms. Thus, suggesting that genes in these families may play critical roles in the biosynthesis and metabolism processes of its unique body plan as well as in regeneration processes.

Using *P. borealis* as the foreground branch and six other echinoderm species as the background branches, we incorporated the branch-site model in the PAML package to detect positively selected genes. A total of 14 genes were positively selected in *P. borealis* (P-value < 0.05, BEB > 0.95) and significantly enriched in GO terms related to “lipid metabolism,” “transport of proton,” “pyruvate metabolism,” and “Hedgehog signaling pathway” (Figure 5, Supplementary Table 5). It is worth noting that these positively selected genes also included BMP4, which regulates regeneration and tissue specification (Table 4).

Regeneration is a high-energy-required process in which starfishes in the regeneration state increase the amount of lipid and energy in the pyloric caeca to use [30]. GPR161 and BMP4, well-known genes to be critical in regeneration, were also detected as positively selected genes. The G-protein coupled receptor Gpr161 negatively regulates the Hedgehog pathway via cAMP signaling, known to participate in the process of tissue regeneration[31, 32]. Additionally, previous studies of planarian regeneration indicate that BMP4 is a key for tissue specification, especially dorsal-ventral polarity, which may explain the distinctive disk of *P. borealis* [33]. Together with those of previous studies, our results further suggest that related genes may have contributed to the regeneration and development of the unique body plan of *P. borealis*,

multiple arms. Therefore, *P. borealis* can be potentially regarded as a valuable model to investigate the mechanisms underlying supernumerary arm development and regeneration. This high-quality genome is useful and valuable genetic resource for future research, especially in a unique body plan and regeneration biology.

Conclusion

The first chromosome-level *P. borealis* genome was assembled and annotated. Twenty-two chromosomal scaffolds are constructed with N50 of 24.97 Mb, which showed high conservation wit-h genomes of three starfish species of the order Forcipulatida. Furthermore, we identified the accelerated evolution of *P. borealis* in the context of genomics, which may explain its multi-armed morphology and regenerative capacity. The availability of the high-quality genome sequence of *P. borealis* is expected to provide many insights into the unique morphology of multi-armed starfish and their regeneration. Regarding the scientific value of *P. borealis*, the genome and gene inventory resulting from this study will be helpful in future research on these critical topics.

Methods

Sampling and genomic DNA extraction

Adult specimens of *P. borealis* were sampled at a depth of 31 meters near Ulleung island, Korea (latitude: 37.53390, longitude: 130.93920) (Figure 1A). *P. borealis* was dissected with scissors to obtain gonad, pyloric caecae, stomach, and epidermis of an arm. Isolated tissues were frozen on dry ice immediately and kept at -80°Cuntil further processing. Then, the frozen tissues were ground into a fine powder with liquid nitrogen using a pestle and mortar for the nucleic acid

extraction.

High molecular weight (HMW) DNA was obtained from gonad following a nuclei isolation method [34]. Genomic DNA was obtained from gonad following modified CTAB protocol [35] in the presence of 2% PVP (1% of MW 10,000 and 1% of MW 40,000) PolyVinylPyrrolidone (Sigma-Aldrich, Burlington, MA, USA). DNA concentration was determined using the Quant-iT PicoGreen® assay (Invitrogen, Waltham, MA, USA) and the absorbance at 260 nm and 230nm (A260/A230) was measured in the Synergy HTX Multi-Mode microplate reader (Biotek, Rochester, VT, USA). Their quality verified by gel electrophoresis.

High-throughput sequencing of genomic DNA

For Nanopore sequencing, short genomic fragments (<10 kb) were removed using a Short Read Eliminator Kit (Circulomics, Baltimore, MD, USA). The library was prepared using the ONT 1D ligation Sequencing kit (SQK-LSK109, Oxford Nanopore Technologies, Oxford, UK) with the native barcoding expansion kit (EXP-NBD104) in accordance with the manufacturer's protocol. In brief, genomic DNA was repaired using the NEBNext FFPE DNA Repair Mix (New England BioLabs, Ipswich, MA, USA) and NEBNext Ultra II End Repair/dA-Tailing Module. The end-prepped DNA was individually barcoded with ONT native barcode by NEB Blunt/TA Ligase Master Mix (New England BioLabs). Barcoded DNA samples were pooled in equal molar amounts. It was ligated with adapter using the NEBNext Quick Ligation Module (New England BioLabs). After every enzyme reaction, the DNA samples were purified using AMPure XP beads (Beckman Coulter, Brea, CA, USA). The final library was loaded onto MinION flow cell (FLO-MIN106 and FLO-MIN111, R9.4 and R10.3) (Oxford Nanopore Technologies) and PromethION flowcell(FLO-PRO002) (Oxford Nanopore Technologies). Sequencing was performed on a MinION MK1b and PromethION sequencer (PromethION, RRID:SCR_017987) with MinKNOW software (19.10.1).

We also used an Illumina platform to generate short high-quality sequencing reads. DNA library was prepared using TruSeq DNA PCR-Free (Illumina, San Diego, CA, USA) and evaluated the distribution of fragment sizes with TapeStation D1000 (Agilent Technologies, Santa Clara, CA, USA). Finally, DNA library was sequenced in the Illumina NovaSeq 6000 (Illumina) (Illumina NovaSeq 6000 Sequencing System, RRID:SCR_016387) with the length of 150 bp paired-end reads.

Hi-C technology was also employed for chromosome-level genome assembly. Hi-C library construction protocol is as follows. Ground gonad tissue was mixed with 1% formaldehyde for fixing chromatin then the nuclei was isolated following a nuclei isolation method [1]. Fixed chromatin was digested with HindII-HF (New England BioLabs), the 5' overhangs filled in with nucleotides and biotin-14-dCTP(Invitrogen) and ligated free blunt ends. After ligation, the DNA purified and removed biotin from un-Ligated DNA ends. Fragmentation and size selection was performed to shear the Hi-C DNA. Hi-C Library preparation is performed using ThruPLEX® DNA-seq Kit (Takara Bio USA, Inc, Mountain View, CA, USA). HI-C library was evaluated the distribution of fragment sizes with TapeStation D1000 (Agilent Technologies, Santa Clara, CA, USA). HI-C library was sequenced in the Illumina NovaSeq 6000 (Illumina) with the length of 150 bp paired-end reads. All of the obtained reads were quality controlled by trimming adaptor sequences and low-quality reads using Trimmomatic v0.39 [36] for Illumina reads and Porechop v0.2.4 [37] (-q 7) and NanoFilt [38] (-k 5000) for Nanopore reads.

Genome size estimation

The quality controlled Illumina sequencing data was used for the calculation of the genome size. Using the reads, a k-mer map was constructed to evaluate genome size, unique sequence ratio, and heterozygosity. For this, jellyfish v2.3.0 (Jellyfish, RRID:SCR_005491) [39] was first used to compute the distribution of the 21-mer frequencies. The final 21-mer count

distribution per genome was used within the GenomeScope 2.0 [40].

Genome assembly and scaffolding with Hi-C data

Multiple approaches were tried but the best assembly was obtained in combination of NextDenovo [41], NextPolish [42] and 3D-DNA [11]. We utilized NextDenovo v2.4.0 to assemble the *P. borealis* genome using only the Nanopore long reads. After the assembly, we applied the Illumina short reads to polish the assembled contigs by operating NextPolish v1.1.0. All software parameter setting were default.

To obtain a chromosome-level genome assembly of *P. borealis*, we employed the Hi-C technology to scaffold assembled contigs. Detailed procedures are as follows. (i) The paired-end Illumina reads were mapped onto the polished assembly using HiC-Pro v3.0.0 (HiC-Pro, RRID:SCR_017643) [43] with default parameters to check the quality of the raw Hi-C reads. (ii) Juicer v1.6 (Juicer, RRID:SCR_017226) [44] and 3D-DNA v180419 [11] were applied to cluster the genomic contig sequences into potential chromosomal groups. (iii) Juicebox v1.13.01 (Juicebox, RRID:SCR_021172) [45] was used to validate the contig orientation and to remove ambiguous fragments with the assistance of manual correction.

Assessment of the chromosome-level genome assembly

Two routine methods were employed to assess the completeness of our finally assembled genome as follows. (i) Bechmarking Universal Single-Copy Orthologues (BUSCO) v5.2.2 (BUSCO, RRID:SCR_015008) [12] assessment: The metazoan_odb10 and eukaryotic_odb10 orthologues were used as the BUSCO reference. (ii) QV score and error rate was estimated with Merqury v1.3 [13].

RNA extraction and sequencing

Total RNA was isolated using TRIzol Reagent(Invitrogen) from three tissues of same *P.*

borealis, digestive gland, stomach and epidermis of arm following the manufacturer's protocol. Total RNA concentration was determined using the Quant-iT™ RNA Assay Kits (Invitrogen) and the absorbance at 260 nm and 280 nm (A260/A280) was measured in the Synergy HTX Multi-Mode microplate reader (Biotek). Their quality verified by gel electrophoresis. mRNA was isolated using Magnosphere™ UltraPure mRNA purification kit(Takara) according to the manufacturer's instructions.

cDNA library was prepared using cDNA-PCR Sequencing Kit (SQK-PCS109, Oxford Nanopore Technologies) with the PCR Barcoding Kit (SQK-PBK004, Oxford Nanopore Technologies) in accordance with the manufacturer's protocol. In brief, RT and strand-switching primers were provided by ONT with the SQK-PCS109 kit. Following RT, PCR amplification was performed using the LongAmpTaq 2X Master Mix (New England Biolabs) and AMpure XP beads (Beckman Coulter) were used for DNA purification. The PCR product was then subjected to ONT adaptor ligation using the SQK-PBK004. The final library was loaded onto MinION flow cell (FLO-MIN106 and FLO-MIN111, R9.4 and R10.3) (Oxford Nanopore Technologies) and sequencing was performed on a MinION MK1b and MinKNOW software (19.10.1).

We also used an Illumina platform to generate short high-quality sequencing reads. Using Truseq Stranded mRNA Prep kit, we constructed cDNA library. After evaluating the distribution of fragment sizes with BioAnalyzer 2100 (Agilent Technologies, Santa Clara, CA, USA), it was sequenced in the Illumina NovaSeq 6000 (Illumina, San Diego, CA, USA) with the length of 100 bp paired-end reads.

Hybrid assembly of transcriptome

To assemble transcriptome, we selected hybrid approach to restore more known genes and discover alternatively spliced isoforms, which can be useful in transcriptome analysis of

previously unsequenced organism. Therefore, long reads and short reads from three tissues were used for assembly. To ensure the accuracy of subsequent analyses, we trimmed the raw reads to remove adaptor sequences and low-quality reads. Trimmomatic v0.39 (Trimmomatic, RRID:SCR_011848) and Porechop v0.2.4 (Porechop, RRID:SCR_016967) were used to trim reads for Illumina and Nanopore reads, respectively. Subsequently, the clean reads were assembled using rnaSPAdes v3.14.1 (rnaSPAdes, RRID:SCR_016992) [46] with default parameters and open reading frames with at least 100 amino acids were extracted from transcripts using TransDecoder (TransDecoder, RRID:SCR_017647) [47].

Annotation of repetitive elements

Repeatitive elements in the final assembly were annotated using the following two different strategies, (i) de novo annotation: RepeatModeler v2.0.1 (RepeatModeler, RRID:SCR_015027) [48] and LTR_Finder v2.0.1 (LTR_Finder, RRID:SCR_015247) [49] were used to build a local repeat reference. Subsequently, the genome assembly was aligned with this reference to annotate the de novo predicted repeat elements using RepeatMasker v4.1.1 (RepeatMasker, RRID:SCR_012954) [50]. (ii) Homology annotation: Our genome assembly was searched in the RepBase (RepeatMaskerEdition) [51] using RepeatMasker v4.1.1. Finally, these data from the two strategies were integrated to generate a nonredundant data set of repetitive elements in the final *P. borealis* genome assembly.

Gene prediction and function annotation

Three methods were used to predict the *P. borealis* gene set from the soft masked *P. borealis* genome. (i) ab initio gene prediction: Augustus v3.4.0 (Augustus, RRID:SCR_008417) [52, 53], GeneMark-ET v3.62 [54], Braker v2.1.5 (BRAKER, RRID:SCR_018964) [55-59] and SNAP v2.51.7 [60] were employed to annotate gene models. (ii) Evidence-based gene prediction: Exonerate (Exonerate, RRID:SCR_016088) [61] were utilized to annotate gene

models with expressed sequence tag (EST) and protein homology dataset. Assembled transcriptome of *P. borealis* were used for EST dataset and protein sequences of *A. rubens* (GCF_902459465.1) from NCBI were used for protein homology dataset. (iii) Consensus gene prediction: EVidenceModeler (EVidenceModeler, RRID:SCR_014659) [62] (EVM) combined predicted ab initio gene models and evidence based gene models into weighed consensus gene structures. This predicted gene set was searched in three public functional databases, including NCBI Nr (nonredundant protein sequences), Swiss-Prot [63] and Pfam database [64] to identify the potential function and functional domains with BLATP v2.10.0+ [65] and Interproscan5 [66].

Gene family expansion and contraction

We downloaded the protein sets of 6 echinoderm species, *Asterias rubens* (GCF_902459465.1), *Acanthaster planci* (GCF_001949145.1), *Patiria miniata* (GCF_015706575.1), *Lytechinus variegatus* (Lvar2.2), *Parastichopus parvimensis* (Pparv_v1.0), and *Strongylocentrotus purpuratus* (GCF_000002235.5) from NBCI and EchinoBase [67] to analyze phylogenetic tree and identify the one-to-one orthologous proteins within the 7 examined species through OrthoFinder v2.5.2 (OrthoFinder, RRID:SCR_017118) [68]. Species tree from OrthoFinder was used to show phylogenetic relationship. Regarding the tree, we used CAFE5 (CAFE, RRID:SCR_005983) [16] to detect gene family expansion and contraction in the assembled *P. borealis* genome with default parameters. GO enrichment using EnrichGO (clusterProfiler v4.0.4) [69] was derived with the Fisher's exact test and chi-square test and then adjusted using the Benjamini-Hochberg procedure.

Genes under positive selection

Positively selected genes in the *P. borealis* genome were detected from one-to-one orthologous genes, in which the *P. borealis* was used as the foreground branch, and the *A. rubens*, *A. planci*,

P. miniata, *L. variegatus*, *P. parvimensis* and *S. purpuratus* were used as the background branches. To detect positively selected genes, we used BLASTP v2.10.0+ (BLASTP, RRID:SCR_001010) to screen out 115 one-to-one orthologous genes among 7 species. The multiple alignment was performed by the GUIDANCE v2.02 software (--msaProgram CLUSTALW, --seqType aa) [70-72] and PAL2NAL v14 [73] was applied to convert protein sequence alignments into the corresponding codon alignments. The branch-site model A incorporated in the PAML package (v4.9j) [74] was employed to detect positively selected genes. The null model used in the branch-site test (model = 2, NSsites =2, fix_omega = 1, omega = 1) assumed that the comparison of the substitution rates at nonsynonymous and synonymous sites (Ka/Ks ratio) for all codons in all branches must be <= 1, whereas the alternative model (model = 2, NSsites =2, fix_omega = 0) assumed that the foreground branch included codons evolving at Ka/Ks > 1. A maximum likelihood ratio test was used to compare the two models. P-values were calculated through the chi-square distribution with 1 degree of freedom (df=1). The P-values were then adjusted for multiple testing using the false discovery rate (FDR) method. Genes were identified as positively selected when the FDR < 0.05. Furthermore, we required that at least one amino-acid site possessed a high probability of being positively selected (Bayes probability > 95%). If none of the amino acids passed this cutoff in the positively selected gene, then these genes were identified as false positives and excluded. GO enrichment using EnrichGO (clusterProfiler v4.0.4) [69] was derived with the Fisher's exact test and chi-square test and then adjusted using the Benjamini-Hochberg procedure with a cutoff set at P-value < 0.05.

Data availability

The final genome assembly and raw data from the Nanopore, Illumina and Hi-C libraries have been deposited at NCBI under BioProject PRJNA776097. Other supporting datasets are available in the *GigaScience* database GigaDB [76].

Abbreviations

BUSCO: Benchmarking Universal Single-Copy Orthologs; BLAST: Basic Local Alignment Search Tool; bp: base pairs; Gb: Giga base pairs; Mb: Mega base pairs; GC: guanine-cytosine; QV: Quality Value; LTR: long terminal repeat; LINE: Long Interspersed Nuclear Elements; SINE: Short Interspersed Nuclear Elements; NR: NCBI's non-redundant database; FDR: False Discovery Rate; GO: Gene Ontology; Bayes empirical Bayes; ONT: Oxford Nanopore Technologies; NCBI: National Center for Biotechnology Information;

Additional Files

Supplementary Figure S1. Genome size estimation

Supplementary Figure S2. *Plazaster borealis* genome assembly completeness. (A) Hi-C interactions among 22 chromosomes. (B) Cumulative length of assembly contained within scaffolds.

Supplementary Table S1. Statistics of raw sequencing data

Supplementary Table S2. Statistics of *Plazaster borealis* genome assembly before scaffolding.

Supplementary Table S3. GO and KEGG enrichment analysis of expanded and contracted gene families of seven echinoderm species.

Supplementary Table S4. GO and KEGG enrichment analysis of *Plazaster borealis* specific orthologs.

Supplementary Table S5. GO and KEGG enrichment analysis of positively selected genes.

Competing Interests

The authors declare that they have no competing interests.

Funding

This work was supported by a grant from the National Institute of Biological Resources (NIBR), funded by the Ministry of Environment (MOE) of the Republic of Korea (NIBR201930201).

Ministry of Environment, National Institute of Biological Resources, NIBR201930201, J Yu;

Authors' Contribution

J.Y., J.P., and S.K. conceived the project; C.B. collected the sample; B.G. performed laboratory experiments; Y.L. and B.K. constructed the assembly; Y.L. annotated the assembly; Y.L. and J.J. performed comparative genome analysis; and Y.L., B.G and S.J. wrote the manuscript with input from all authors.

Acknowledgements

We thank the reviewers for their helpful comments and constructive suggestions on the manuscript. We also appreciate to the NIBR for the support.

References

1. Garcia-Arraras JE and Dolmatov IY. Echinoderms: potential model systems for studies on muscle regeneration. Curr Pharm Des. 2010;16 8:942-55. doi:10.2174/138161210790883426.
2. Carnevali, M. C. (2006). Regeneration in Echinoderms: repair, regrowth, cloning. Invertebrate Survival Journal, 3(1), 64-76.
3. Sprinkle J. Patterns and problems in echinoderm evolution. Echinoderm Studies. CRC Press;

2020. p. 1-18.

4. Nichols D. Pentamerism and the Calcite Skeleton in Echinoderms. Nature. 1967;215 5101:665-6. doi:10.1038/215665a0.

5. Stephenson DG. Pentameral Symmetry in Echinoderms. Nature. 1967;216 5119:994-. doi:10.1038/216994a0.

6. Hotchkiss FHC. On the Number of Rays in Starfish1. American Zoologist. 2015;40 3:340-54. doi:10.1093/icb/40.3.340.

7. Sook S. A Systematic Study on the Asteroidea in the East Sea, Korea. Animal Systematics, Evolution and Diversity. 1995;11 2:243-63.

8. Uchida T. Report of the Biological Survey of Mutsu Bay. 11. Starfishes of Mutsu Bay. Scientific Reports of Tohoku Imperial University. 1928.

9. Hayashi R. Contributions to the Classification of the Sea-stars of Japan.: II. Forcipulata, with the Note on the Relationships between the Skeletal Structure and Respiratory Organs of the Sea-stars (With 11 Plates and 115 textfigures). 北海道帝國大學理學部紀要. 1943;8 3:133-281.

10. Matsuoka N, Fukuda K, Yoshida K, Sugawara M and Inamori M. Biochemical systematics of five asteroids of the family Asteriidae based on allozyme variation. Zoological science. 1994;11 2:p343-9.

11. Dudchenko O, Batra SS, Omer AD, Nyquist SK, Hoeger M, Durand NC, et al. De novo assembly of the Aedes aegypti genome using Hi-C yields chromosome-length scaffolds. Science. 2017;356 6333:92-5. doi:10.1126/science.aal3327.

12. Simao FA, Waterhouse RM, Ioannidis P, Kriventseva EV and Zdobnov EM. BUSCO: assessing genome assembly and annotation completeness with single-copy orthologs. Bioinformatics. 2015;31 19:3210-2. doi:10.1093/bioinformatics/btv351.

13. Rhie A, Walenz BP, Koren S and Phillippy AM. Merqury: reference-free quality, completeness, and phasing assessment for genome assemblies. Genome Biol. 2020;21 1:245. doi:10.1186/s13059-020-02134-9.

14. Wang Y, Tang H, Debarry JD, Tan X, Li J, Wang X, et al. MCScanX: a toolkit for detection and evolutionary analysis of gene synteny and collinearity. Nucleic Acids Res. 2012;40 7:e49. doi:10.1093/nar/gkr1293.

15. Perez-Wohlfeil E, Diaz-Del-Pino S and Trelles O. Ultra-fast genome comparison for large-scale genomic experiments. Sci Rep. 2019;9 1:10274. doi:10.1038/s41598-019-46773-w.

16. Mendes FK, Vanderpool D, Fulton B and Hahn MW. CAFE 5 models variation in evolutionary rates among gene families. Bioinformatics. 2020; doi:10.1093/bioinformatics/btaa1022.

17. Artavanis-Tsakonas S, Rand MD and Lake RJ. Notch signaling: cell fate control and signal integration in development. Science. 1999;284 5415:770-6. doi:10.1126/science.284.5415.770.

18. Lai EC. Notch signaling: control of cell communication and cell fate. Development. 2004;131 5:965-73. doi:10.1242/dev.01074.

19. Sato C, Zhao G and Ilagan MX. An overview of notch signaling in adult tissue renewal and maintenance. Curr Alzheimer Res. 2012;9 2:227-40. doi:10.2174/156720512799361600.

20. Bocci F, Onuchic JN and Jolly MK. Understanding the Principles of Pattern Formation Driven by Notch Signaling by Integrating Experiments and Theoretical Models. Front Physiol. 2020;11:929. doi:10.3389/fphys.2020.00929.
21. de Celis JF, Tyler DM, de Celis J and Bray SJ. Notch signalling mediates segmentation of the Drosophila leg. Development. 1998;125 23:4617-26.
22. Cordoba S and Estella C. Role of Notch Signaling in Leg Development in Drosophila melanogaster. Adv Exp Med Biol. 2020;1218:103-27. doi:10.1007/978-3-030-34436-8_7.
23. Lapraz F, Besnardeau L and Lepage T. Patterning of the Dorsal-Ventral Axis in Echinoderms: Insights into the Evolution of the BMP-Chordin Signaling Network. PLOS Biology. 2009;7 11:e1000248. doi:10.1371/journal.pbio.1000248.
24. Dhanasekaran DN and Reddy EP. JNK signaling in apoptosis. Oncogene. 2008;27 48:6245-51. doi:10.1038/onc.2008.301.
25. Heisenberg CP and Bellaiche Y. Forces in tissue morphogenesis and patterning. Cell. 2013;153 5:948-62. doi:10.1016/j.cell.2013.05.008.
26. Mashanov V, Akiona J, Khoury M, Ferrier J, Reid R, Machado DJ, et al. Active Notch signaling is required for arm regeneration in a brittle star. PLoS One. 2020;15 5:e0232981. doi:10.1371/journal.pone.0232981.
27. Reinardy HC, Emerson CE, Manley JM and Bodnar AG. Tissue regeneration and biomineralization in sea urchins: role of Notch signaling and presence of stem cell markers. PLoS One. 2015;10 8:e0133860. doi:10.1371/journal.pone.0133860.
28. Shao Y, Wang XB, Zhang JJ, Li ML, Wu SS, Ma XY, et al. Genome and single-cell RNA-sequencing of the earthworm Eisenia andrei identifies cellular mechanisms underlying regeneration. Nat Commun. 2020;11 1:2656. doi:10.1038/s41467-020-16454-8.
29. Frederick HCH. A "Rays-as-Appendages" Model for the Origin of Pentamerism in Echinoderms. Paleobiology. 1998;24 2:200-14.
30. Rubilar T, Villares G, Epherra L, Díaz-de-Vivar ME and Pastor-de-Ward CT. Fission, regeneration, gonad production and lipids storage in the pyloric caeca of the sea star Allostichaster capensis. Journal of Experimental Marine Biology and Ecology. 2011;409 1:247-52. doi:https://doi.org/10.1016/j.jembe.2011.09.004.
31. Warner JF, Miranda EL and McClay DR. Contribution of hedgehog signaling to the establishment of left-right asymmetry in the sea urchin. Dev Biol. 2016;411 2:314-24. doi:10.1016/j.ydbio.2016.02.008.
32. Mukhopadhyay S, Wen X, Ratti N, Loktev A, Rangell L, Scales SJ, et al. The ciliary G-protein-coupled receptor Gpr161 negatively regulates the Sonic hedgehog pathway via cAMP signaling. Cell. 2013;152 1-2:210-23. doi:10.1016/j.cell.2012.12.026.
33. Reddien PW. Constitutive gene expression and the specification of tissue identity in adult planarian biology. Trends Genet. 2011;27 7:277-85. doi:10.1016/j.tig.2011.04.004.
34. Zhang M, Zhang Y, Scheuring CF, Wu CC, Dong JJ and Zhang HB. Preparation of megabase-sized DNA from a variety of organisms using the nuclei method for advanced genomics

research. Nat Protoc. 2012;7 3:467-78. doi:10.1038/nprot.2011.455.

35. Porebski S, Bailey LG and Baum BR. Modification of a CTAB DNA extraction protocol for plants containing high polysaccharide and polyphenol components. Plant Molecular Biology Reporter. 1997;15 1:8-15. doi:10.1007/BF02772108.

36. Bolger AM, Lohse M and Usadel B. Trimmomatic: a flexible trimmer for Illumina sequence data. Bioinformatics. 2014;30 15:2114-20. doi:10.1093/bioinformatics/btu170.

37. Porechop. https://github.com/rrwick/Porechop (2017).

38. De Coster W, D'Hert S, Schultz DT, Cruts M and Van Broeckhoven C. NanoPack: visualizing and processing long-read sequencing data. Bioinformatics. 2018;34 15:2666-9. doi:10.1093/bioinformatics/bty149.

39. Marcais G and Kingsford C. A fast, lock-free approach for efficient parallel counting of occurrences of k-mers. Bioinformatics. 2011;27 6:764-70. doi:10.1093/bioinformatics/btr011.

40. Ranallo-Benavidez TR, Jaron KS and Schatz MC. GenomeScope 2.0 and Smudgeplot for reference-free profiling of polyploid genomes. Nat Commun. 2020;11 1:1432. doi:10.1038/s41467-020-14998-3.

41. NextOmics: NextDeNovo. https://github.com/Nextomics/NextDenovo (2019).

42. Hu J, Fan J, Sun Z and Liu S. NextPolish: a fast and efficient genome polishing tool for long-read assembly. Bioinformatics. 2020;36 7:2253-5. doi:10.1093/bioinformatics/btz891.

43. Servant N, Varoquaux N, Lajoie BR, Viara E, Chen CJ, Vert JP, et al. HiC-Pro: an optimized and flexible pipeline for Hi-C data processing. Genome Biol. 2015;16:259. doi:10.1186/s13059-015-0831-x.

44. Durand NC, Shamim MS, Machol I, Rao SS, Huntley MH, Lander ES, et al. Juicer Provides a One-Click System for Analyzing Loop-Resolution Hi-C Experiments. Cell Syst. 2016;3 1:95-8. doi:10.1016/j.cels.2016.07.002.

45. Durand NC, Robinson JT, Shamim MS, Machol I, Mesirov JP, Lander ES, et al. Juicebox Provides a Visualization System for Hi-C Contact Maps with Unlimited Zoom. Cell Syst. 2016;3 1:99-101. doi:10.1016/j.cels.2015.07.012.

46. Bushmanova E, Antipov D, Lapidus A and Prjibelski AD. rnaSPAdes: a de novo transcriptome assembler and its application to RNA-Seq data. Gigascience. 2019;8 9 doi:10.1093/gigascience/giz100.

47. TransDecoder. https://github.com/TransDecoder/TransDecoder (2015).

48. Flynn JM, Hubley R, Goubert C, Rosen J, Clark AG, Feschotte C, et al. RepeatModeler2 for automated genomic discovery of transposable element families. Proc Natl Acad Sci U S A. 2020;117 17:9451-7. doi:10.1073/pnas.1921046117.

49. Xu Z and Wang H. LTR_FINDER: an efficient tool for the prediction of full-length LTR retrotransposons. Nucleic Acids Res. 2007;35 Web Server issue:W265-8. doi:10.1093/nar/gkm286.

50. Smit A, Hubley, R & Green, P: RepeatMasker Open-4.0. http://www.repeatmasker.org (2013-2015).

51. Bao W, Kojima KK and Kohany O. Repbase Update, a database of repetitive elements in eukaryotic genomes. Mob DNA. 2015;6:11. doi:10.1186/s13100-015-0041-9.
52. Stanke M, Diekhans M, Baertsch R and Haussler D. Using native and syntenically mapped cDNA alignments to improve de novo gene finding. Bioinformatics. 2008;24 5:637-44. doi:10.1093/bioinformatics/btn013.
53. Stanke M, Schoffmann O, Morgenstern B and Waack S. Gene prediction in eukaryotes with a generalized hidden Markov model that uses hints from external sources. BMC Bioinformatics. 2006;7:62. doi:10.1186/1471-2105-7-62.
54. Lomsadze A, Burns PD and Borodovsky M. Integration of mapped RNA-Seq reads into automatic training of eukaryotic gene finding algorithm. Nucleic Acids Res. 2014;42 15:e119. doi:10.1093/nar/gku557.
55. Hoff KJ, Lange S, Lomsadze A, Borodovsky M and Stanke M. BRAKER1: Unsupervised RNA-Seq-Based Genome Annotation with GeneMark-ET and AUGUSTUS. Bioinformatics. 2016;32 5:767-9. doi:10.1093/bioinformatics/btv661.
56. Hoff KJ, Lomsadze A, Borodovsky M and Stanke M. Whole-Genome Annotation with BRAKER. Methods Mol Biol. 2019;1962:65-95. doi:10.1007/978-1-4939-9173-0_5.
57. Li H, Handsaker B, Wysoker A, Fennell T, Ruan J, Homer N, et al. The Sequence Alignment/Map format and SAMtools. Bioinformatics. 2009;25 16:2078-9. doi:10.1093/bioinformatics/btp352.
58. Barnett DW, Garrison EK, Quinlan AR, Stromberg MP and Marth GT. BamTools: a C++ API and toolkit for analyzing and managing BAM files. Bioinformatics. 2011;27 12:1691-2. doi:10.1093/bioinformatics/btr174.
59. Buchfink B, Xie C and Huson DH. Fast and sensitive protein alignment using DIAMOND. Nat Methods. 2015;12 1:59-60. doi:10.1038/nmeth.3176.
60. Leskovec J and Sosic R. SNAP: A General Purpose Network Analysis and Graph Mining Library. ACM Trans Intell Syst Technol. 2016;8 1 doi:10.1145/2898361.
61. Slater GS and Birney E. Automated generation of heuristics for biological sequence comparison. BMC Bioinformatics. 2005;6:31. doi:10.1186/1471-2105-6-31.
62. Haas BJ, Salzberg SL, Zhu W, Pertea M, Allen JE, Orvis J, et al. Automated eukaryotic gene structure annotation using EVidenceModeler and the Program to Assemble Spliced Alignments. Genome Biol. 2008;9 1:R7. doi:10.1186/gb-2008-9-1-r7.
63. Bairoch A and Apweiler R. The SWISS-PROT protein sequence database and its supplement TrEMBL in 2000. Nucleic Acids Res. 2000;28 1:45-8. doi:10.1093/nar/28.1.45.
64. Mistry J, Chuguransky S, Williams L, Qureshi M, Salazar Gustavo A, Sonnhammer ELL, et al. Pfam: The protein families database in 2021. Nucleic Acids Research. 2020;49 D1:D412-D9. doi:10.1093/nar/gkaa913.
65. Camacho C, Coulouris G, Avagyan V, Ma N, Papadopoulos J, Bealer K, et al. BLAST+: architecture and applications. BMC Bioinformatics. 2009;10:421. doi:10.1186/1471-2105-10-421.

66. Jones P, Binns D, Chang HY, Fraser M, Li W, McAnulla C, et al. InterProScan 5: genome-scale protein function classification. Bioinformatics. 2014;30 9:1236-40. doi:10.1093/bioinformatics/btu031.
67. Kudtarkar P and Cameron RA. Echinobase: an expanding resource for echinoderm genomic information. Database (Oxford). 2017;2017 doi:10.1093/database/bax074. http://www.echinobase.org
68. Emms DM and Kelly S. OrthoFinder: phylogenetic orthology inference for comparative genomics. Genome Biology. 2019;20 1:238. doi:10.1186/s13059-019-1832-y.
69. Wu T, Hu E, Xu S, Chen M, Guo P, Dai Z, et al. clusterProfiler 4.0: A universal enrichment tool for interpreting omics data. Innovation (N Y). 2021;2 3:100141. doi:10.1016/j.xinn.2021.100141.
70. Penn O, Privman E, Ashkenazy H, Landan G, Graur D and Pupko T. GUIDANCE: a web server for assessing alignment confidence scores. Nucleic Acids Res. 2010;38 Web Server issue:W23-8. doi:10.1093/nar/gkq443.
71. Sela I, Ashkenazy H, Katoh K and Pupko T. GUIDANCE2: accurate detection of unreliable alignment regions accounting for the uncertainty of multiple parameters. Nucleic Acids Res. 2015;43 W1:W7-14. doi:10.1093/nar/gkv318.
72. Landan G and Graur D. Local reliability measures from sets of co-optimal multiple sequence alignments. Pac Symp Biocomput. 2008:15-24.
73. Suyama M, Torrents D and Bork P. PAL2NAL: robust conversion of protein sequence alignments into the corresponding codon alignments. Nucleic Acids Res. 2006;34 Web Server issue:W609-12. doi:10.1093/nar/gkl315.
74. Yang Z. PAML 4: phylogenetic analysis by maximum likelihood. Mol Biol Evol. 2007;24 8:1586-91. doi:10.1093/molbev/msm088.
75. Emms D.M. and Kelly S. STAG: Species Tree Inference from All Genes. bioRixv. 2018. doi: https://doi.org/10.1101/267914
76. Lee Y; Kim B; Jung J; Koh B; Jhang SY; Ban C; Chi W; Kim S; Yu J (2022): Supporting data for "Chromosome-level genome assembly of Plazaster borealis: shed light on the morphogenesis of multi-armed starfish and its regenerative capacity" GigaScience Database. http://dx.doi.org/10.5524/102219
77. National Institute of Biological Resources. https://www.nibr.go.kr

Figures

Figure 1: A. Adult *Plazaster borealis*. Photograph by National Institute of Biological Resources [77] B. Sampling spot of *P. borealis* studied in this research.

Figure 2: A phylogenetic tree of *P. borealis* and six other species. This tree was constructed using protein sequences of seven species, showing gene family expansion and contraction. The number below the branches represents the number of gene families with either expansion (blue) and contraction (red). The ratio of expanded and contracted gene families was expressed in the pie chart above the branches. The numbers at the node indicate the bootstrap value. The species used in the tree are *P. borealis*, *Asterias rubens*, *Acanthaster planci*, *Patiria miniata*, *Lytechinus variegatus*, *Parastichopus parvimensis*, and *Strongylocentrotus purpuratus*.

Figure 3: Syntenic relationship of *P. borealis* and species of the order Forcipulatida. A. Synteny between *Asterias rubens* and *P. borealis.* The syntenic blocks were calculated with MCscan. B-D. Syntenic relationship of *P. borealis* between *A. rubens* (B), *Pisaster ochraceus* (C), *Marthasterias glacialis* (D) Genomic sequences were compared with Chromeister based on inexact k-mer matching.

Figure 4: GO enrichment analysis of expanded gene families of *P. borealis.*

Figure 5: Results of GO enrichment analysis of positively selected genes. BP: GO Term Biological Process (green), CC: GO Term Cellular Component (red), KEGG: Kyoto Encyclopedia of Genes and Genomes (blue).

Figure1

Click here to access/download;Figure;Figure1_Adult_Plazaster_borealis_and_sam

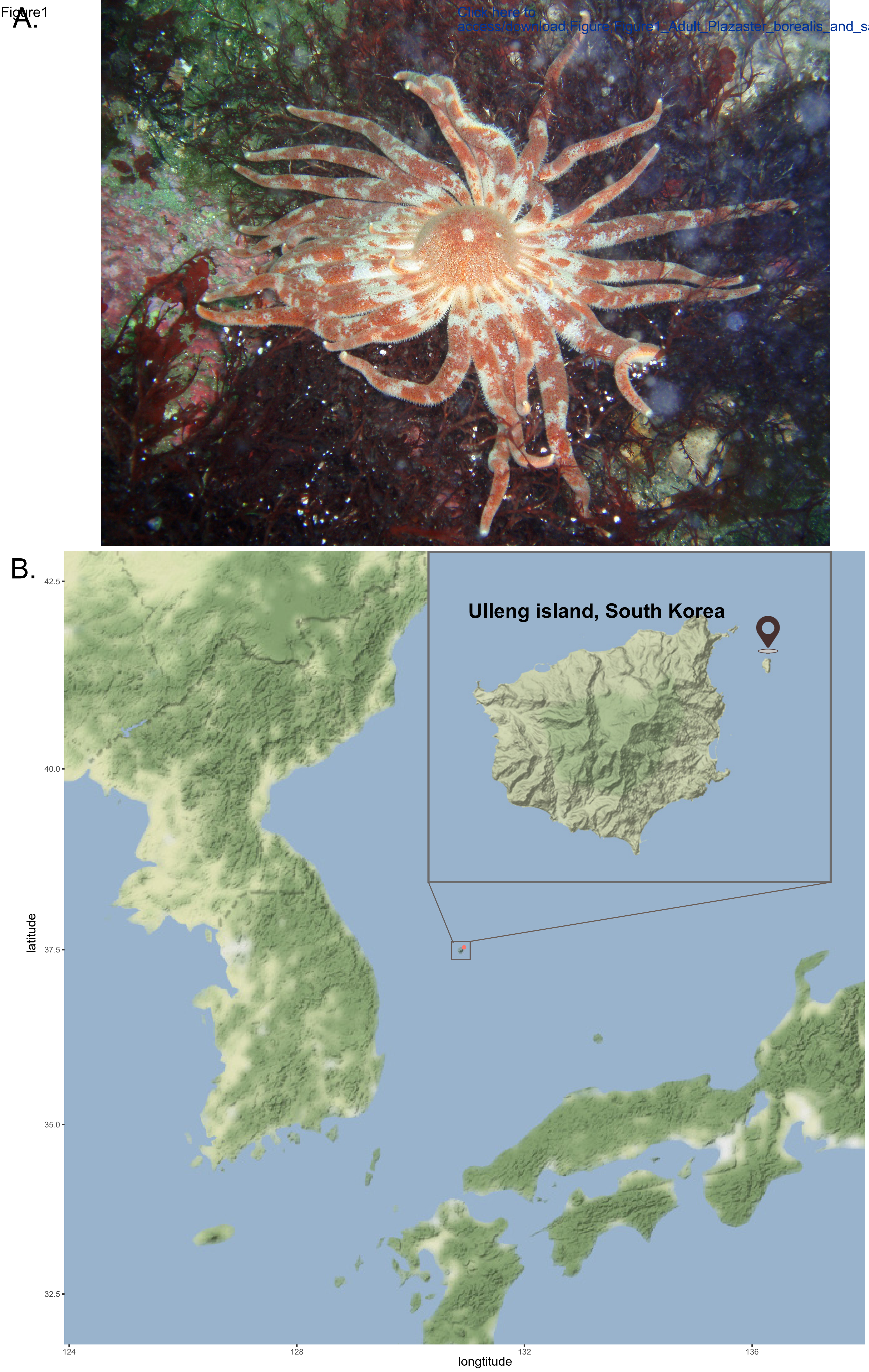

Figure2

Click here to access/download;Figure;Figure2_Phylogeny_tree.pdf

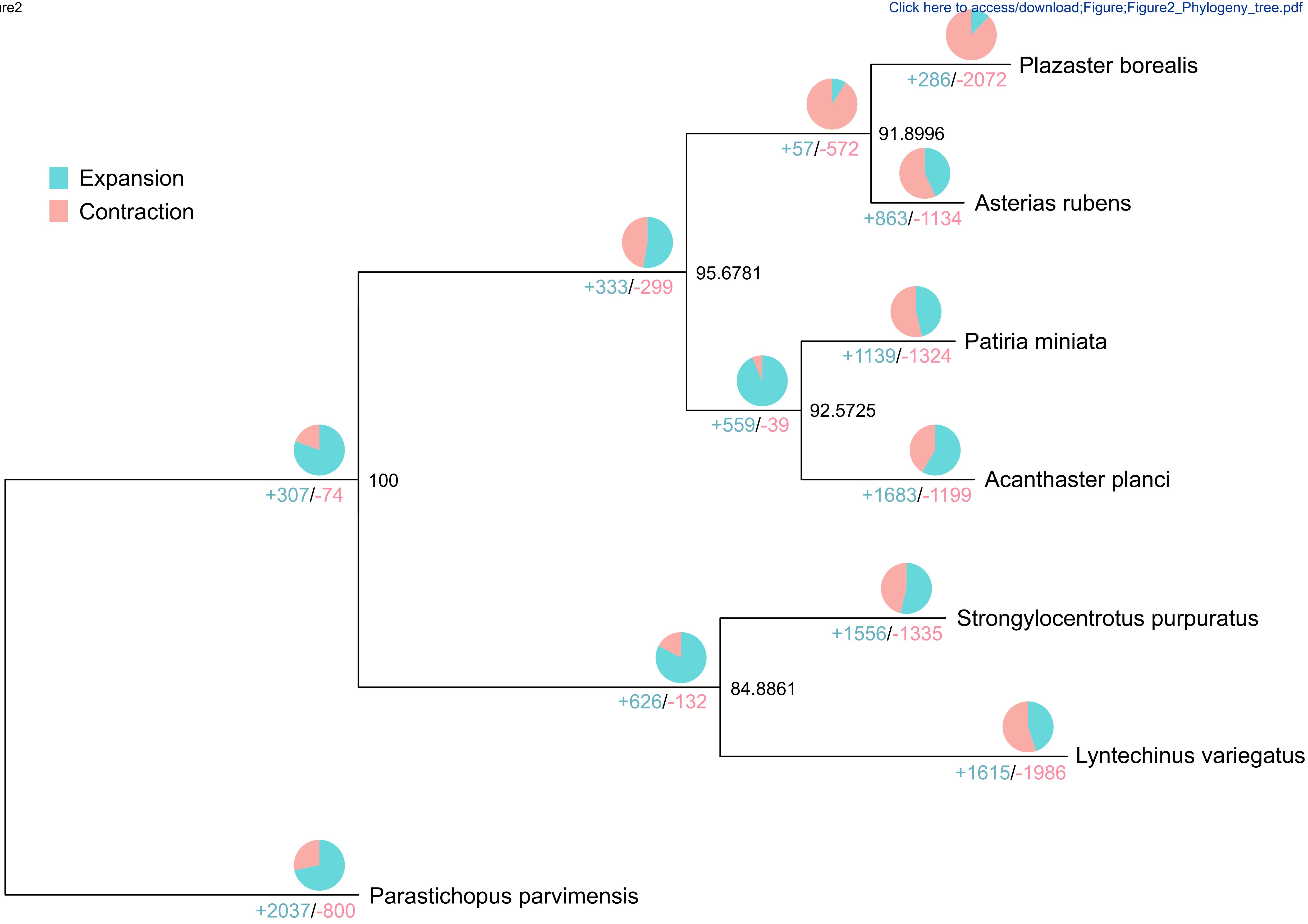

Figure3

Click here to access/download;Figure;Figure3_Syntenic_relationship.p

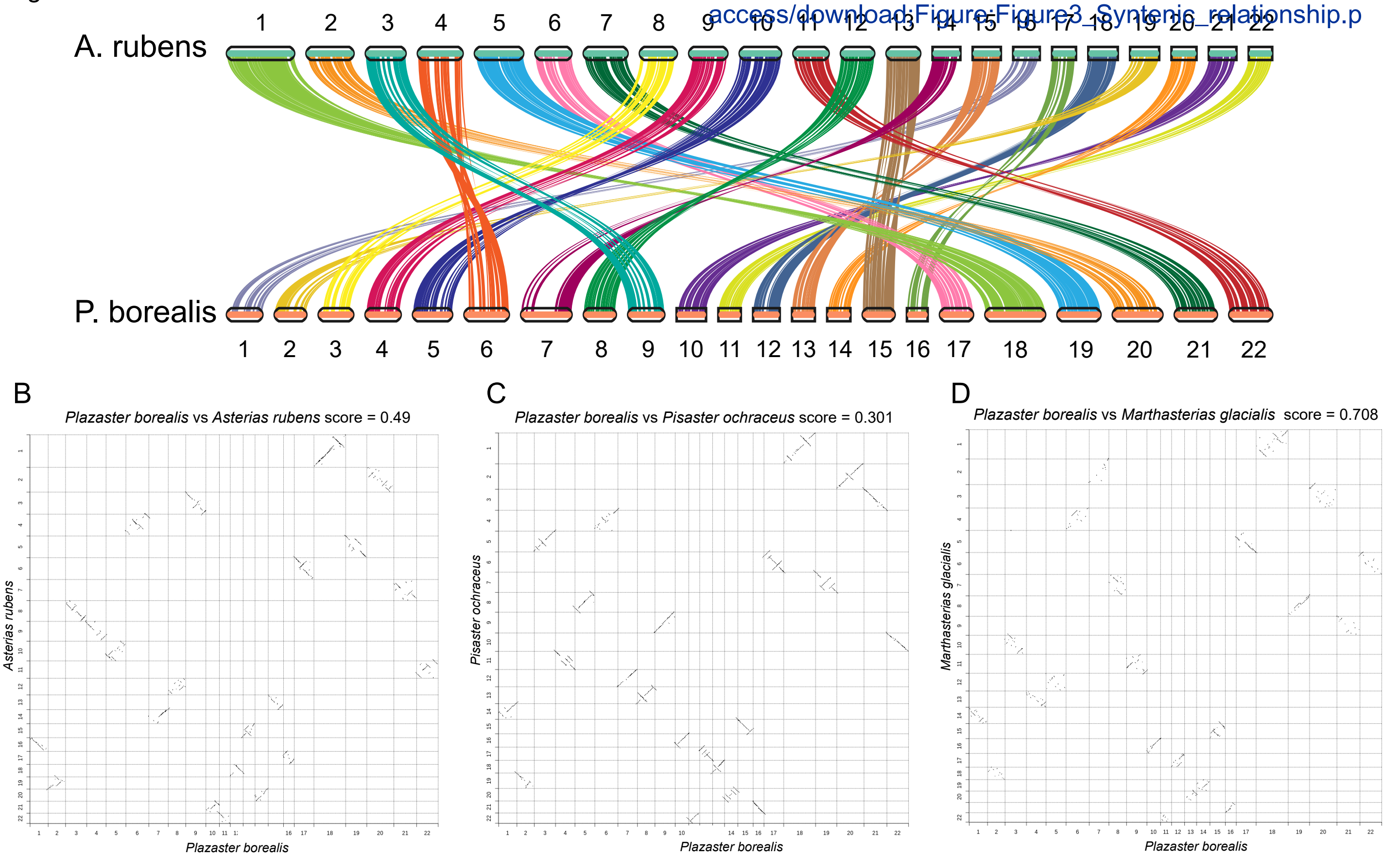

Figure4

Click here to access/download;Figure;Figure4_GO_enrichment_of_exp_gene_Pborealis.pdf

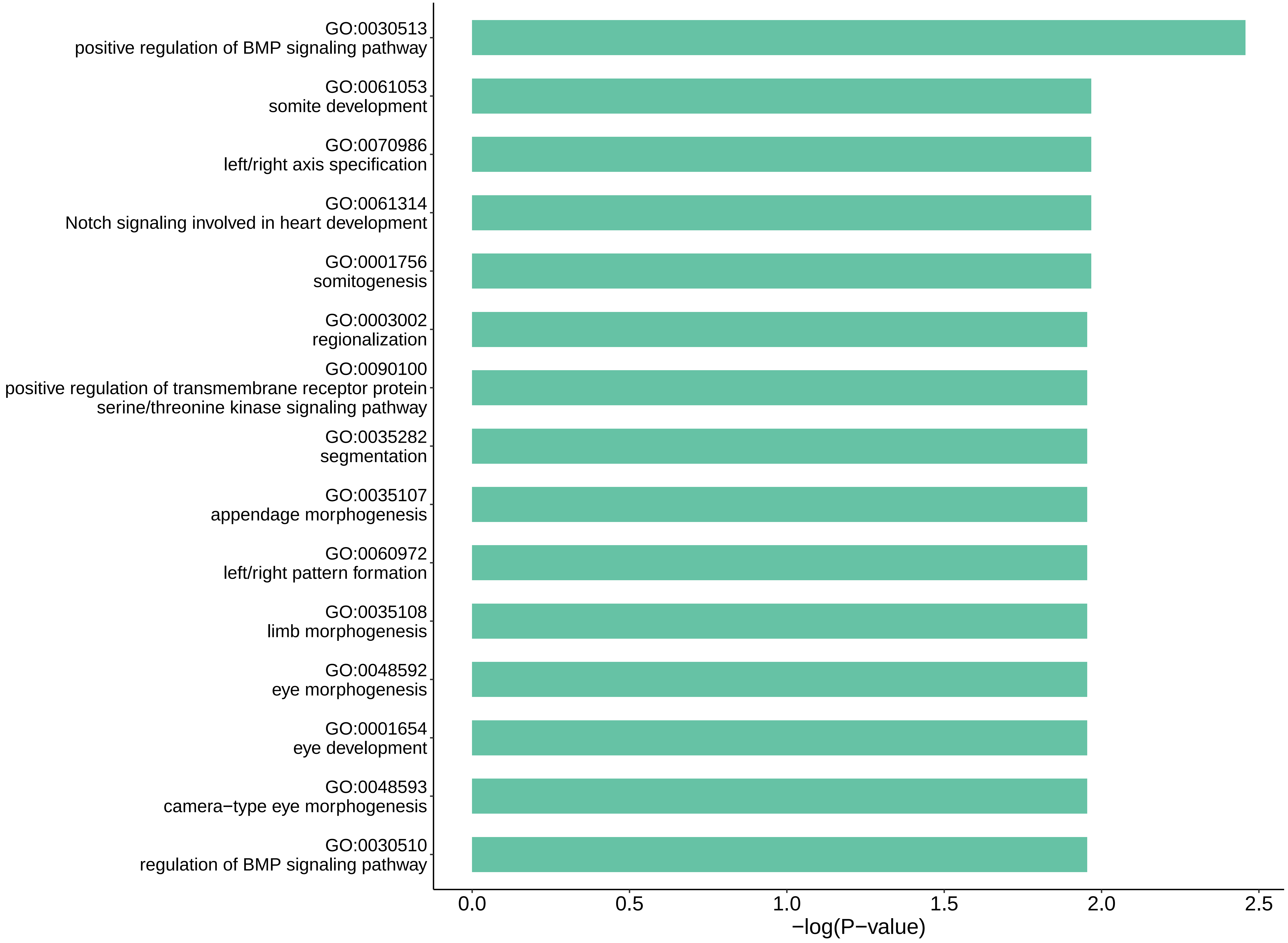

Click here to access/download;Figure;Figure5_GO_enrichment_of_positively_selected_genes.pdf

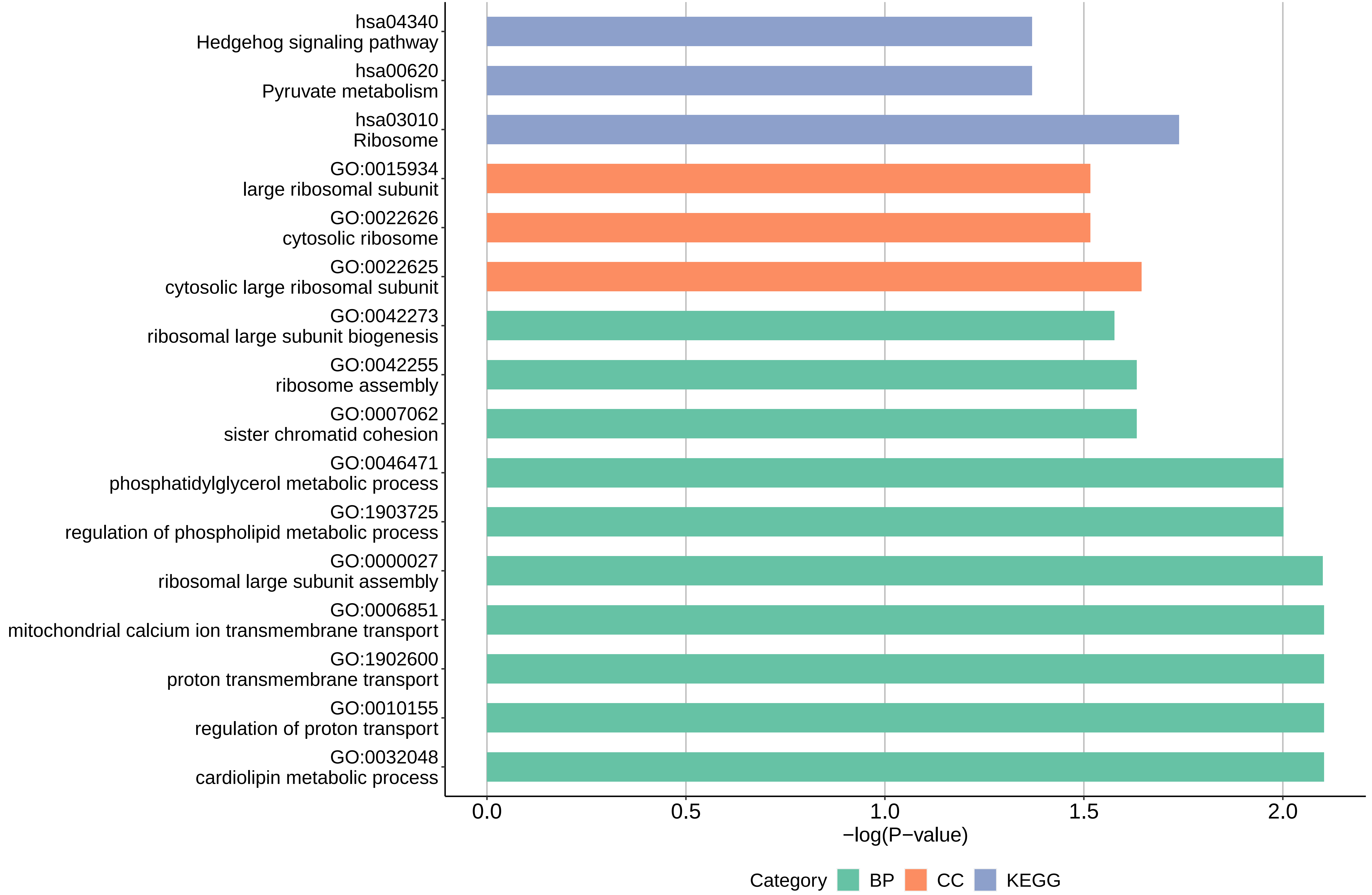

Supp-Fig1

Click here to access/download
Supplementary Material
Supp_Fig1_Genome_size_estimation.png

Supp-Fig2

Click here to access/download
Supplementary Material
Supp_Fig2_Plazaster_borealis_genome_assembly_completeness.pdf

Click here to access/download
Supplementary Material
Supp_Table1_Statistics_of_raw_sequencing_data.xlsx

Click here to access/download
Supplementary Material
Supp_Table2_Statistics_of_assembly_before_scaffolding
.xlsx

Click here to access/download
Supplementary Material
Supp_Table3_GO_KEGG_enrichment_of_con&exp_of_7_echinoderms.xlsx

Click here to access/download
Supplementary Material
Supp_Table4_GO_KEGG_enrichment_of_Pborealis_specific_ortho.xlsx

Supp-Tab5

Click here to access/download
Supplementary Material
Supp_Table5_GO_KEGG_enrichment_of_positively_selected_genes.xlsx
