# Supplementary figures and images for: Chromosome-level genome assembly of Plazaster borealis sheds light on the morphogenesis of multiarmed starfish and its regenerative capacity
Source: Gigascience. 2022 Jul 9;11:giac063. doi: 10.1093/gigascience/giac063 (PMC9270726; doi:10.1093/gigascience/giac063)

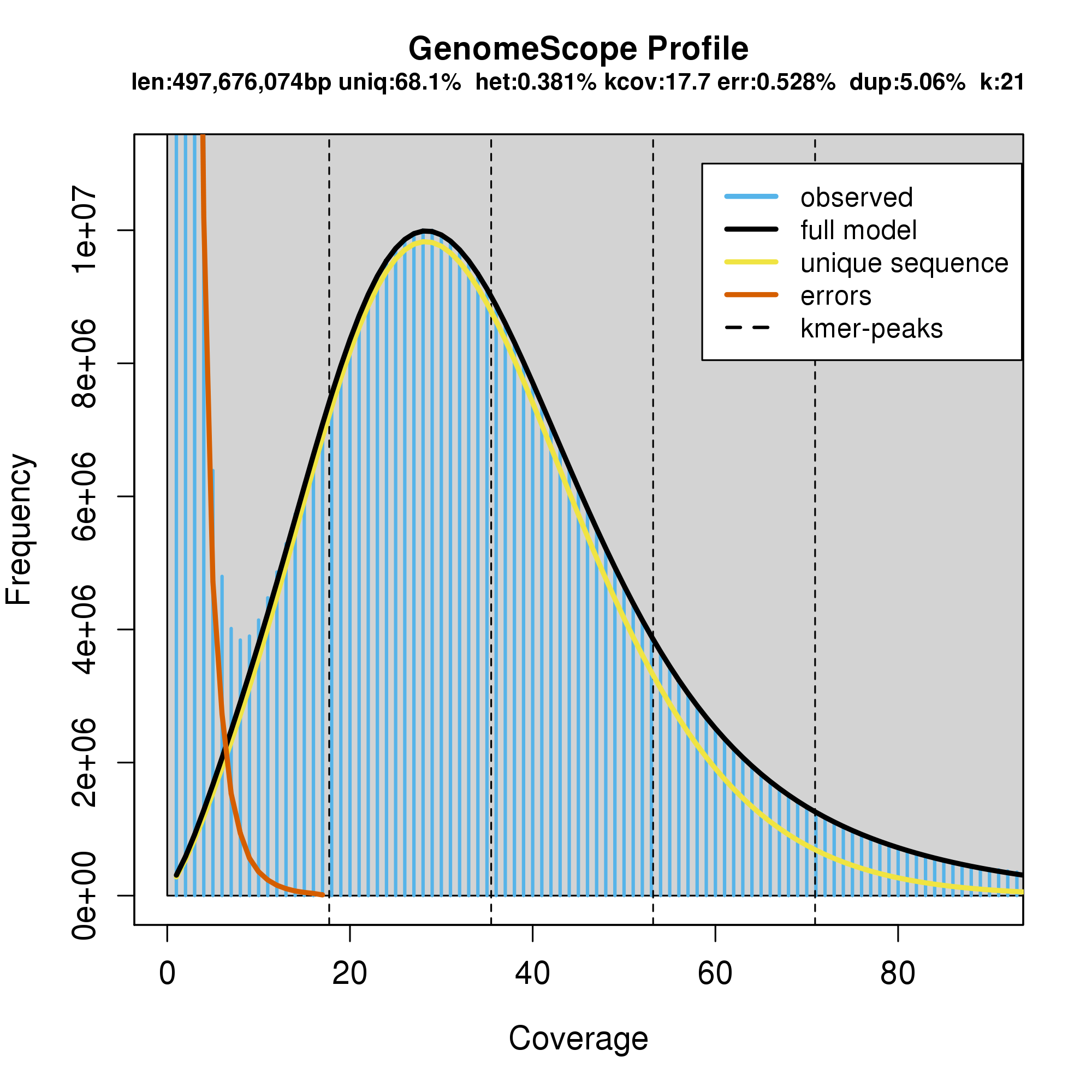

Supplement: giac063_Supplemental_Files [file giac063_supplemental_files.zip › Supp_Fig1_Genome_size_estimation.png]

**A**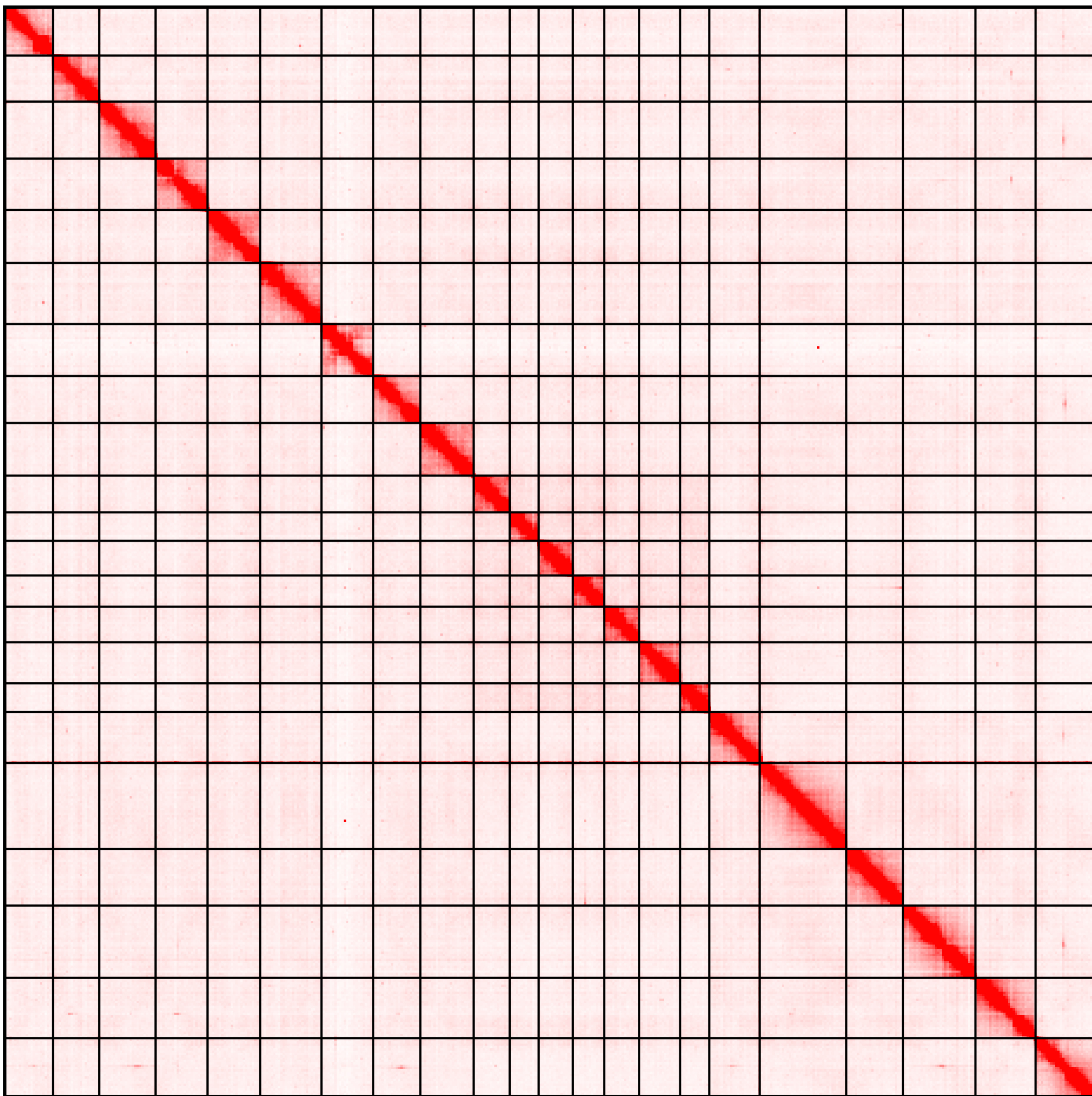**B**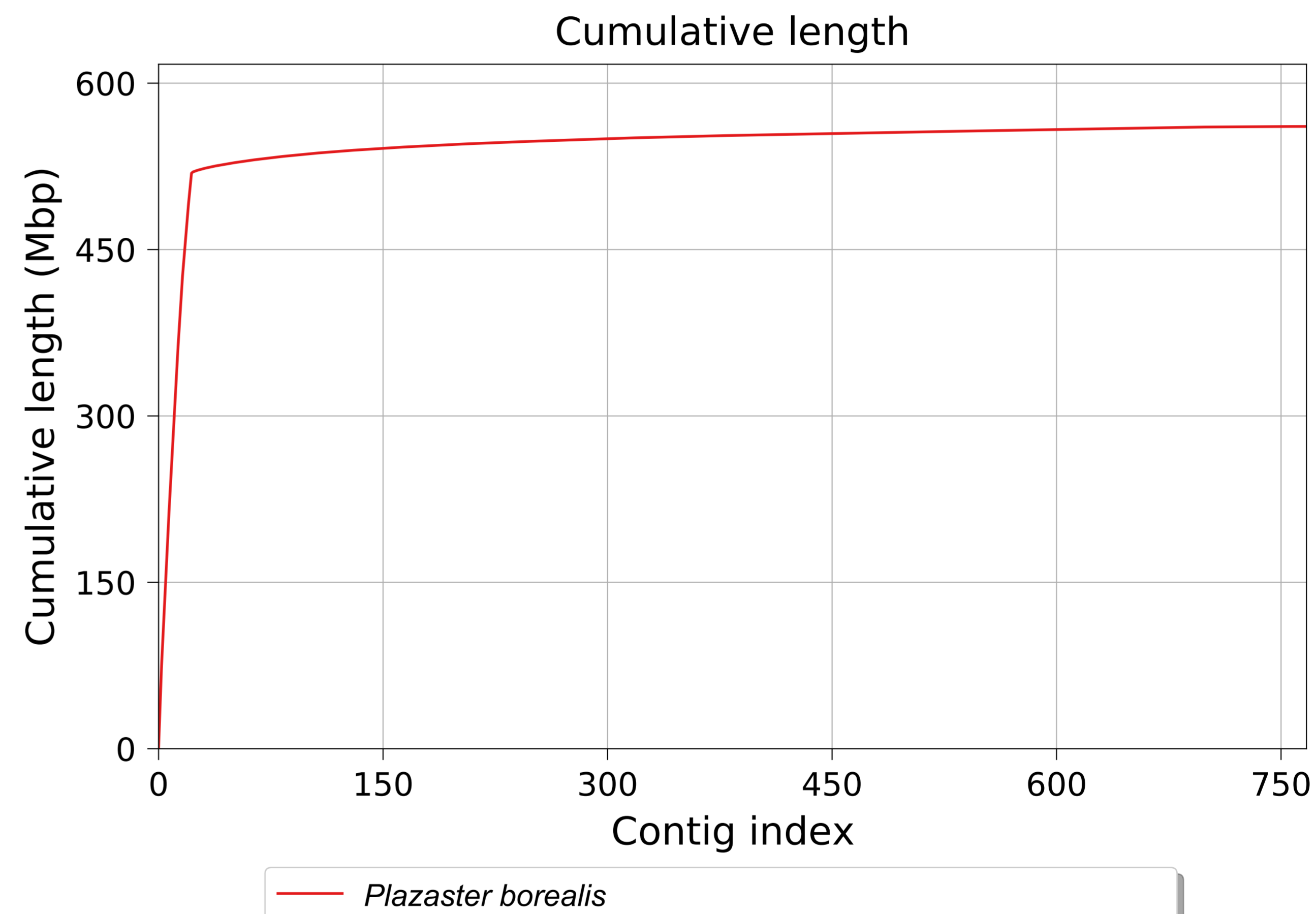

Supplement: giac063_Supplemental_Files [file giac063_supplemental_files.zip › Supp_Fig2_Plazaster_borealis_genome_assembly_completeness.pdf]
