# Supplementary material for: Chromosome-level genome assembly of Plazaster borealis sheds light on the morphogenesis of multiarmed starfish and its regenerative capacity
Source: Gigascience. 2022 Jul 9;11:giac063. doi: 10.1093/gigascience/giac063 (PMC9270726; doi:10.1093/gigascience/giac063)
Supplement: giac063_Response_to_Reviewer_Comments_Original_Submission [file giac063_response_to_reviewer_comments_original_submission.pdf]

# Author's Response To Reviewer Comments

Close

Reviewer #1:
1. Suggestions and editions of the language.

We revised all the suggested sentences in the manuscript.

2. How did you measure significance here: "The significantly expanded genes in the genome of P. borealis were significantly enriched in categories of Notch and BMP signaling pathway, body pattern specification, morphogenesis, and eye development (P<0.02) (Figure 4).

CAFE5 implements a birth-death model for evolutionary inferences about gene family evolution. Its main task is the maximum-likelihood estimation of a global or local gene family evolutionary rates for a given data set. From the output 'model_branch_probabilities.txt', we could get the probabilities calculated for each clade and significant family. The gene families satisfying cut off 0.05 were used as significantly expanded or reduced genes.
The 'P-value < 0.02' used to get significantly enriched GO terms was determined to filter out comprehensive GO terms.

3. Did you do correction for multiple testing? both for the GO term analysis and the PAML analysis.

Yes. We described it in the manuscript line 372-379.

4. Which model of PAML was used? Model A? Please describe the null model and the one that allowed for positive selection.

We used branch site A model with null model (model = 2, NSsites =2, fix_omega = 1, omega = 1) and alternative model (model = 2, NSsites =2, fix_omega = 0). We supplemented the details in the manuscript. (line 365-368)

Reviewer #2:
1. Suggestions and editions of the language.

We revised all the suggested sentences in the manuscript.

2. Page 7: "Each gene sets consisted as following, S: 97.6%, 97.0%; D: 1.2%, 1.0%; F: 0.8%, 1.2%; and M: 0.4%, 0.8%. (S: single-copy, D: duplicated, F: fragmental, M: missed of eukaryotic_odb10 and metabozan_odb10 data set, respectively)"
>> This is awkwardly written. It would be best to write these out in sentences, but at the very least instead of providing a key just use the term. For example: "Each gene sets consisted as following: single-copy 97.6%..."
>> Also the second number is not explained. Is it needed? If so, explain it. Or just relegate the details to a supplemental table?

The second number was the BUSCO values based on the eukaryotic_odb10 gene set. The value was added to emphasize the genome completeness but we agree that it is redundant value. So we revised the manuscript (line 94-97).

3. Page 8: "To understand the phylogenetic location of P. borealis, we used a BLAST-based hierarchical clustering algorithm for genome-wide phylogenetic analysis based on protein sequences from seven echinoderm genomes."
>> The phylogeny is not acceptable. There is no description of how orthologs were called, there is no details of the program used to generate alignment or phylogeny. Hierarchical clustering is not an acceptable phylogenetic method. I recommend using single-copy orthologs from OrthoFinder or

Orthomcl, aligning them with MAFFT, and using a maximum-likelihood algorithm to generate the tree. IQTREE or RAxML with automatic model determination would work.

We used 'species tree' calculated from the OrthoFinder2 to show the phylogenetic relationship of P. borealis among the 6 echinoderm species. The OrthoFinder2 infers phylogenetic relationship of the species in a way that is not much different from your recommendation: 1) orthogroup inference, 2) inference of gene trees for each orthogroup, and 3) analysis of these gene trees to infer the rooted species tree.
After identifying orthogroups, the OrthoFinder2 uses these orthogroups to infer gene trees for all orthogroups. The inferred gene trees were analyzed to identify the species tree using STAG algorithm. STAG was developed to leverage the vast amount of phylogenetic information already available in the complete set of orthogroup gene trees inferred by OrthoFinder. It was also developed to be robust to high levels of gene duplication and loss that can hamper methods that rely on sets of single-copy orthologs. The method subsequently identifies all gene duplication events in the complete set of gene trees and analyzes this information in the context of the species tree.
We revised ambiguous sentences about the phylogenetic relationship in the manuscript. (line 118-122, 358-359)

4. Page 8: "Syntenic relationships analyzed by MCscan [12] also proved their relationship."
>> However, the synteny scores between P. borealis and Pisaster ochraceus show more conservation than between P. borealis and A. rubens, suggesting that the synteny scores do not support that relationship. It is problematic that P. glacialis and P. ochraceus are not included in the phylogeny but are included in the synteny. Adding both to the phylogeny would help with the interpretation of the result.

M. glacialis and P. ochraceus were not able to be included in the phylogeny due to the absence of protein sequence data. The synteny score estimated with Chromeister indicates the similarity between genomes. With 0 indicating the exact same sequences and 1 indicating absolutely no similarity.
Among 6 echinoderm species analyzed in the phylogenetic tree, A. rubens showed the closest relationship with P. borealis and synteny analysis also supported this relationship. The syntenic relationship with other starfish in Forcipulatida order, M. glacialis and P. ochraceus, showed that Forcipulatida order tends to have considerably conserved genome. Furthermore, they revealed high quality of constructed genome of P. borealis. We revised related contents in the manuscript. (line 123-129)

5. Page 8: " These results suggest that genomes within the Forcipulatida order are remarkably conserved in terms of synteny and chromosome, supporting the high quality of the assembled genome."
>> There were no comparisons reported of non forcipulatid genomes, so this statement is problematic.

We checked the syntenic relationship of P. borealis and A. planci which is non-Forcipulatida order starfish. The chromosomes of two species were not matched. We stated it in the manuscript. (line 129-131)

6. We indicated in the manuscript that the other supporting data could be available in the GigaScience Database. While submitting the manuscript, we uploaded all the protein, transcript, and annotation file on the GigaDB FTP. The DOI of the dataset will be given a DOI on acceptance for the publication.

Close
