# Supplementary material for: Chromosome-level genome assembly of Plazaster borealis sheds light on the morphogenesis of multiarmed starfish and its regenerative capacity
Source: Gigascience. 2022 Jul 9;11:giac063. doi: 10.1093/gigascience/giac063 (PMC9270726; doi:10.1093/gigascience/giac063)
Supplement: giac063_Response_to_Reviewer_Comments_Revision_1 [file giac063_response_to_reviewer_comments_revision_1.pdf]

Author's Response To Reviewer Comments

Close

Reviewer #2:
1. Suggestions and editions of the language.

We revised all the suggested sentences in the manuscript.

Close
