# Supplementary material for: Chromosome-level genome assembly of Plazaster borealis sheds light on the morphogenesis of multiarmed starfish and its regenerative capacity
Source: Gigascience. 2022 Jul 9;11:giac063. doi: 10.1093/gigascience/giac063 (PMC9270726; doi:10.1093/gigascience/giac063)
Supplement: giac063_Reviewer_1_Report_Original_Submission — Rute R. da Fonseca -- 12/9/2021 Reviewed [file giac063_reviewer_1_report_original_submission.pdf]

**Reviewer Report**

**Title: Chromosome-level genome assembly of Plazaster borealis: shed light on the morphogenesis of multi-armed starfish and its regenerative capacity**

**Version: Original Submission** **Date:** 12/9/2021

**Reviewer name: Rute R. da Fonseca**

**Reviewer Comments to Author:**

This paper describe the genome of a multi-armed starfish that has an unusual morphology. The authors did not add line numbers to the paper, making the marking quite tedious. Maybe this can be changed if there is another round of reviews. I present some suggestion for improving the clarity of the text, and ask for more information regarding the data.

Answers to the basic questions asked by the journal

1) Are the methods appropriate to the aims of the study, are they well described, and are necessary controls included?

Better description of the methods and statistical tests is needed

2) Are the conclusions adequately supported by the data shown?

The authors should be careful when stating that they found the basis for the morphological uniqueness and regeneration capability of these starfish.

3) Please indicate the quality of language in the manuscript. Does it require a heavy editing for language and clarity?

I provide some suggestions and edits to the language.

4) Are you able to assess all statistics in the manuscript, including the appropriateness of statistical tests used?

I need more information from the authors as specified below.

Suggestion for Title: "Chromosome-level genome assembly of Plazaster borealis sheds light on the morphogenesis of multi-armed starfish and its regenerative capacity"

Suggestions for Abstract:

Background: Plazaster borealis (Uchida, 1928) has a unique morphology displaying multiple arms with clear distinction between disk and arms, rather than the remarkable pentaradial symmetry characteristic of Echinoderms. Herein we report the first chromosome-level reference genome of P. borealis, and essential tool to further investigate the base for this divergent morphology.

Findings: Please state the amount of data (Gb) generated rather than saying "high coverage"

Please rephrase: "The genome completeness estimated by BUSCO is of 98.8% using the eukaryote gene set and of 98.0% using the metazoan set, indicative of a high-quality assembly."

Conclusion: Please be more conservative in your final statement: "as well as suggesting the genomic mechanisms underlying its unique morphology and regeneration capability".

Suggestions for Context:

"Echinoderms are invertebrates"

"2) a water vascular system used for locomotion, food, waste etc (mention exactly what this is used for

in echinoderms [3],"
"Penta-radial symmetry has been observed"
"raising questions about the apparent morphology of echinoderm" - what do you mean? Raising questions about the genetic basis for the non-penta-radial symmetry?
I suggest you rephrase: "The octopus starfish, Plazaster borealis, is a starfish that inhabits the waters that surround Korea and Japan. It belongs…"
"As can be seen in figure 1A, it has an unique morphology including around 31~40 arms, a large number among starfishes. Also shows a clear differentiation between arms and disk."
Again I suggest you to edit this same sentence you had in the abstract. The accelerated evolutionary rate you are mentioned is tested here at the sequence level, not the morphological level that is why you need a genome: "This possibly resulted from accelerated sequence evolution, something that can only be assessed with a reference genome".
Suggestions for the Completeness of the assembled genome:
BUSCO needs a reference.
"each gene set"
Suggestions for Annotation of repeats and genes:
A. rubens needs a reference.
Suggestions for Phylogenetic and syntenic relationship:
"phylogenetic placement" not location
"consistent with both previous results" not taxa, I guess
"Syntenic relationships analyzed by MCscan [12] confirmed their relationship. The chromosomes of P. borealis mostly matched those of A. rubens, except for chromosome 7 of P. borealis (Figure 3A, 3B) that shows a large unmatched region." (could be a deletion in others, how do you know it is an expansion)
"These results suggest that genomes within the Forcipulatida order are remarkably conserved in terms of synteny, and that allows us to confirm the high quality of our genome assembly"
Suggestions for Gene family evolution in P. borealis:
CAFE is missing a reference.
How did you measure significance here: "The significantly expanded genes in the genome of P. borealis were significantly enriched in categories of Notch and BMP signaling pathway, body pattern specification, morphogenesis, and eye development (P<0.02) (Figure 4)." Also here p-value is denoted "P" and below is "p-value"and in the figures is "P-value". Choose a single notation.
Did you do correction for multiple testing? Both for the GO term analysis and the PAML analysis?
Drosophila melanogaster should be in italic.
Which model of PAML was used? Model A? Please describe the null model and the one that allowed for positive selection.

**Level of Interest**

Please indicate how interesting you found the manuscript: Choose an item.

**Quality of Written English**

Please indicate the quality of language in the manuscript: Choose an item.

**Declaration of Competing Interests**

Please complete a declaration of competing interests, considering the following questions:

- Have you in the past five years received reimbursements, fees, funding, or salary from an organisation that may in any way gain or lose financially from the publication of this manuscript, either now or in the future?
- Do you hold any stocks or shares in an organisation that may in any way gain or lose financially from the publication of this manuscript, either now or in the future?
- Do you hold or are you currently applying for any patents relating to the content of the manuscript?
- Have you received reimbursements, fees, funding, or salary from an organization that holds or has applied for patents relating to the content of the manuscript?
- Do you have any other financial competing interests?
- Do you have any non-financial competing interests in relation to this paper?

If you can answer no to all of the above, write 'I declare that I have no competing interests' below. If your reply is yes to any, please give details below.

I declare that I have no competing interests

I agree to the open peer review policy of the journal. I understand that my name will be included on my report to the authors and, if the manuscript is accepted for publication, my named report including any attachments I upload will be posted on the website along with the authors' responses. I agree for my report to be made available under an Open Access Creative Commons CC-BY license (http://creativecommons.org/licenses/by/4.0/). I understand that any comments which I do not wish to be included in my named report can be included as confidential comments to the editors, which will not be published.

Choose an item.

To further support our reviewers, we have joined with Publons, where you can gain additional credit to further highlight your hard work (see: https://publons.com/journal/530/gigascience). On publication of this paper, your review will be automatically added to Publons, you can then choose whether or not to claim your Publons credit. I understand this statement.

Yes Choose an item.
