# Supplementary material for: Chromosome-level genome assembly of Plazaster borealis sheds light on the morphogenesis of multiarmed starfish and its regenerative capacity
Source: Gigascience. 2022 Jul 9;11:giac063. doi: 10.1093/gigascience/giac063 (PMC9270726; doi:10.1093/gigascience/giac063)
Supplement: giac063_Reviewer_2_Report_Original_Submission — Joseph F. Ryan -- 12/21/2021 Reviewed [file giac063_reviewer_2_report_original_submission.pdf]

Reviewer Report

Title: Chromosome-level genome assembly of Plazaster borealis: shed light on the morphogenesis of multi-armed starfish and its regenerative capacity

Version: Original Submission **Date:** 12/21/2021

Reviewer name: Joseph F. Ryan

Reviewer Comments to Author:

This is an exciting animal and a high quality genome!
However, the paper is not strong and there are issues with almost every sentence.
One major issue I have noticed is that the descriptions of the analyses do not include command lines or software versions and thus are not repeatable. It is very important that all analyses include these information and every effort is made (including providing scripts etc.) to make sure all analyses can be repeated with a reasonable amount of effort and without having to contact the authors. I highly recommend providing a document like the file named "Zhou_etal_supplementary_text-2nd_revised.docx" that is included in the supplement of the following paper:
https://doi.org/10.1093/molbev/msx302
Also, the gene models (nucleotide, protein, and GFF files) should be made available and included in the Data Availability section.
Below are some detailed edits for the first 8 pages.
Page 4: "Echinoderms are various invertebrate marine animals that belong to the phylum Echinodermata."
>> This is not necessary/redundant. Would make sense to start with the 2nd sentence. "Echinoderms are marine animals characterized by the following three remarkable characteristics..."
Page 4: sentence starting with "They have three remarkable characteristics"
>> possible grammar issue: Needs an "and" before the third characteristic.
>> possible grammar issue: "extraordinary morphological characteristics, penta-radial symmetry" is not correct. Maybe "extraordinary morphological characteristics including pentaradial symmetry."
Page 4 (and beyond): "penta-radial"
>> should be "pentaradial"
Page 5: "was observed in all extant classes"
>> possible grammar issue: might be better as "has been observed in all extant classes"
Page 5: "are scattered across both time and taxa"
>> I don't understand "time" here. Might be better as "scattered across the tree of Echinodermata"
Page 5: "raising questions about the apparent morphology of echinoderm, penta-radial symmetry"
>> possible grammar issue: I don't understand this.
Page 5: "shows a clear differentiation between arms and disk"
>> "arms and central disk" would be helpful for non-starfish biologists
Page 5: "differentiated from the starfish with standard morphology: five arms and no distinction of arms and central disk"

>> possible grammar issue: might be better as "descended from a five-armed starfish ."

Page 5: "the absence of reference genome limited advanced research in depth"

>> possible grammar issue: might be better as "the absence of a reference genome has limited in depth research."

Page 6: "sequences of other six echinoderms"

>> possible grammar issue: might be better as: "sequences of six other echinoderms"

Page 6: "A total 561Mb of draft P. borealis genome was assembled into 179 contigs with N50 of 11Mb"

>> possible grammar issue: might be better as "A draft genome assembly was generated that consisted of 179 contigs totaling 561Mb with an N50 of 11Mb"

Page 6: "we scaffolded the contigs using 3D-DNA"

>> there is no mention of Hi-C data here so this is confusing.

Page 7: "Each gene sets consisted as following, S: 97.6%, 97.0%; D: 1.2%, 1.0%; F: 0.8%, 1.2%; and M: 0.4%, 0.8%. (S: single-copy, D: duplicated, F: fragmental, M: missed of eukaryotic_odb10 and metabozan_odb10 data set, respectively)"

>> This is awkwardly written. It would be best to write these out in sentences, but at the very least instead of providing a key just use the term. For example: "Each gene sets consisted as following: single-copy 97.6%..."

>> Also the second number is not explained. Is it needed? If so, explain it. Or just relegate the details to a supplemental table?

Page 8: "To understand the phylogenetic location of P. borealis, we used a BLAST-based hierarchical clustering algorithm for genome-wide phylogenetic analysis based on protein sequences from seven echinoderm genomes."

>> The phylogeny is not acceptable. There is no description of how orthologs were called, there is no details of the program used to generate alignment or phylogeny. Hierarchical clustering is not an acceptable phylogenetic method. I recommend using single-copy orthologs from OrthoFinder or Orthomcl, aligning them with MAFFT, and using a maximum-likelihood algorithm to generate the tree. IQTREE or RAxML with automatic model determination would work.

Page 8: "Syntenic relationships analyzed by MCscan [12] also proved their relationship."

>> there is no explanation for how syntenic relationships prove the relationship. It might be more accurate to say: "syntenic relationships analyzed by MCscan [12] were consistent with this relationship."

>> However, the synteny scores between P. borealis and Pisaster ochraceus show more conservation than between P. borealis and A. rubens, suggesting that the synteny scores do not support that relationship. It is problematic that P. glacialis and P. ochraceus are not included in the phylogeny but are included in the synteny. Adding both to the phylogeny would help with the interpretation of the result.

Page 8: "P. ochraceus was the most conserved with P. borealis"

>> possible grammar issue: might be better as "P. ochraceus exhibited the highest level of conservation of synteny with P. borealis"

Page 8: " These results suggest that genomes within the Forcipulatida order are remarkably conserved in terms of synteny and chromosome, supporting the high quality of the assembled genome."

>> There were no comparisons reported of non forcipulatid genomes, so this statement is problematic.

Page 8: "Based on the assumption that the unique morphology of P. borealis is explained by accelerated evolutionary rate [10], we performed comparative genomic analyses among seven echinoderm species."

>> The rest of this paragraph does not address this first sentence. I would remove it or address evolutionary rate directly.

Level of Interest

Please indicate how interesting you found the manuscript: Choose an item.

Quality of Written English

Please indicate the quality of language in the manuscript: Choose an item.

Declaration of Competing Interests

Please complete a declaration of competing interests, considering the following questions:

- Have you in the past five years received reimbursements, fees, funding, or salary from an organisation that may in any way gain or lose financially from the publication of this manuscript, either now or in the future?
- Do you hold any stocks or shares in an organisation that may in any way gain or lose financially from the publication of this manuscript, either now or in the future?
- Do you hold or are you currently applying for any patents relating to the content of the manuscript?
- Have you received reimbursements, fees, funding, or salary from an organization that holds or has applied for patents relating to the content of the manuscript?
- Do you have any other financial competing interests?
- Do you have any non-financial competing interests in relation to this paper?

If you can answer no to all of the above, write 'I declare that I have no competing interests' below. If your reply is yes to any, please give details below.

I declare that I have no competing interests

I agree to the open peer review policy of the journal. I understand that my name will be included on my report to the authors and, if the manuscript is accepted for publication, my named report including any attachments I upload will be posted on the website along with the authors' responses. I agree for my report to be made available under an Open Access Creative Commons CC-BY license (http://creativecommons.org/licenses/by/4.0/). I understand that any comments which I do not wish to be included in my named report can be included as confidential comments to the editors, which will not be published.

Choose an item.

To further support our reviewers, we have joined with Publons, where you can gain additional credit to further highlight your hard work (see: https://publons.com/journal/530/gigascience). On publication of this paper, your review will be automatically added to Publons, you can then choose whether or not to claim your Publons credit. I understand this statement.

Yes Choose an item.
