# Supplementary material for: Chromosome-level genome assembly of Plazaster borealis sheds light on the morphogenesis of multiarmed starfish and its regenerative capacity
Source: Gigascience. 2022 Jul 9;11:giac063. doi: 10.1093/gigascience/giac063 (PMC9270726; doi:10.1093/gigascience/giac063)
Supplement: giac063_Reviewer_2_Report_Revision_1 — Joseph F. Ryan -- 3/28/2022 Reviewed [file giac063_reviewer_2_report_revision_1.pdf]

Reviewer Report

Title: Chromosome-level genome assembly of Plazaster borealis: shed light on the morphogenesis of multi-armed starfish and its regenerative capacity

Version: Revision 1 **Date:** 3/28/2022

Reviewer name: Joseph F. Ryan

Reviewer Comments to Author:

This revision is substantially improved. My recommendation assumes that all data is made available (including raw data, genome assembly, gene models, transcriptome assemblies) in GigaDB and/or another stable repository. I'm including some suggestions for individual sentences in the manuscript below.

1> First sentence. "Plazaster borealis has a unique morphology displaying multiple arms with a clear distinction between disk and arms, rather than remarkable characteristic of Echinoderms"
-- It is not clear what remarkable characteristic is being referred to here.
2> Page 10 LINE 123: "Syntenic relationships analyzed by MCscan [14] also proved their relationship"
-- "proved their relationship" is problematic (grammatically and in meaning). A more succinct replacement might be "Syntenic relationships as inferred by MCscan [14] results were congruent with the phylogenetic results from the STAG analyses."
3> Page 11 LINE 139: "it is expected that genes associated..."
-- This would be stronger and more accurate if written as "we hypothesized that genes associated..." and if that change were made then "To investigate the expanded gene families, we performed expansion and contraction analysis of gene families using CAFE5" (on LINE 140) would be better written as "We tested this hypothesis by performing expansion and contraction analyses of gene families using CAFE5."
4> LINE 147: "It is generally accepted that Notch and BMP signaling are evolutionally conserved and play multiple roles during animal development, especially in regulating body patterns."
-- It would be better to remove "It is generally accepted that" and start the sentence with "Notch and BMP signaling are..."
5> LINE 175: "The signaling pathways detected through expanded gene families"
-- would be better as "The signaling pathways that underwent gene family expansion in the P. borealis lineage"
6> LINE 204: GRAMMAR: "known to participate tissue regeneration process"
-- "known to participate in the process of tissue regeneration"
7> LINE 211: "We believe that this high-quality genome will supply a useful..."
-- I believe it too! I would be more certain here. Maybe: "This high-quality genome is a useful..."
8> LINE 329: "transcriptomes with at least 100 amino acids were extracted using TransDecoder"
-- I think the authors meant: "Open reading frames with at least 100 amino acids were extracted from transcripts using TransDecoder"

Level of Interest

Please indicate how interesting you found the manuscript: Choose an item.

Quality of Written English

Please indicate the quality of language in the manuscript: Choose an item.

Declaration of Competing Interests

Please complete a declaration of competing interests, considering the following questions:

- Have you in the past five years received reimbursements, fees, funding, or salary from an organisation that may in any way gain or lose financially from the publication of this manuscript, either now or in the future?
- Do you hold any stocks or shares in an organisation that may in any way gain or lose financially from the publication of this manuscript, either now or in the future?
- Do you hold or are you currently applying for any patents relating to the content of the manuscript?
- Have you received reimbursements, fees, funding, or salary from an organization that holds or has applied for patents relating to the content of the manuscript?
- Do you have any other financial competing interests?
- Do you have any non-financial competing interests in relation to this paper?

If you can answer no to all of the above, write 'I declare that I have no competing interests' below. If your reply is yes to any, please give details below.

I declare that I have no competing interests.

I agree to the open peer review policy of the journal. I understand that my name will be included on my report to the authors and, if the manuscript is accepted for publication, my named report including any attachments I upload will be posted on the website along with the authors' responses. I agree for my report to be made available under an Open Access Creative Commons CC-BY license (http://creativecommons.org/licenses/by/4.0/). I understand that any comments which I do not wish to be included in my named report can be included as confidential comments to the editors, which will not be published.

Choose an item.

To further support our reviewers, we have joined with Publons, where you can gain additional credit to further highlight your hard work (see: https://publons.com/journal/530/gigascience). On publication of this paper, your review will be automatically added to Publons, you can then choose whether or not to claim your Publons credit. I understand this statement.

Yes Choose an item.
